# Ototopical drops containing a novel antibacterial synthetic peptide: Safety and efficacy in adults with chronic suppurative otitis media

**DOI:** 10.1371/journal.pone.0231573

**Published:** 2020-04-14

**Authors:** Nanno F. A. W. Peek, Marja J. Nell, Ronald Brand, Thekla Jansen-Werkhoven, Ewoud J. van Hoogdalem, Ruud Verrijk, Marcel J. Vonk, Amon R. Wafelman, A. Rob P. M. Valentijn, Johan H. M. Frijns, Pieter S. Hiemstra, Jan W. Drijfhout, Peter H. Nibbering, Jan J. Grote

**Affiliations:** 1 Department of Ear Nose and Throat, Leiden University Medical Centre (LUMC), Leiden, The Netherlands; 2 Octoplus Technologies BV/Dr Reddy’s research and development BV, Leiden, The Netherlands; 3 Department of Biomedical Data Sciences, Leiden University Medical Centre, Leiden, The Netherlands; 4 Department of Pulmonology, Leiden University Medical Centre, Leiden, The Netherlands; 5 Clinical Pharmacology and Toxicology, Leiden University Medical Centre, Leiden, The Netherlands; 6 Immunohematology and Blood Transfusion, Leiden University Medical Centre, Leiden, The Netherlands; 7 Department of Infectious Diseases, Leiden University Medical Centre, Leiden, The Netherlands; University of Connecticut Health Center, UNITED STATES

## Abstract

**Objective:**

Chronic suppurative otitis media (CSOM) is a chronic infectious disease with worldwide prevalence that causes hearing loss and decreased quality of life. As current (antibiotic) treatments often unsuccessful and antibiotic resistance is emerging, alternative agents and/or strategies are urgently needed. We considered the synthetic antimicrobial and anti-biofilm peptide P60.4Ac to be an interesting candidate because it also displays anti-inflammatory activities including lipopolysaccharide-neutralizing activity. The aim of the present study was to investigate the safety and efficacy of ototopical drops containing P60.4Ac in adults with CSOM without cholesteatoma.

**Methods:**

We conducted a range-finding study in 16 subjects followed by a randomized, double blinded, placebo-controlled, multicentre phase II^a^ study in 34 subjects. P60.4Ac-containing ototopical drops or placebo drops were applied twice a day for 2 weeks and adverse events (AEs) and medication use were recorded. Laboratory tests, swabs from the middle ear and throat for bacterial cultures, and audiometry were performed at intervals up to 10 weeks after therapy. Response to treatment was assessed by blinded symptom scoring on otoscopy.

**Results:**

Application of P60.4Ac-containing ototopical drops (0.25–2.0 mg of peptide/ml) in the ear canal of patients suffering from CSOM was found to be safe and well-tolerated. The optimal dose (0.5 mg of peptide/ml) was selected for the subsequent phase II^a^ study. Safety evaluation revealed only a few AEs that were unlikely related to study treatment and all, except one, were of mild to moderate intensity. In addition to this excellent safety profile, P60.4Ac ototopical drops resulted in a treatment success in 47% of cases versus 6% in the placebo group.

**Conclusion:**

The efficacy/safety balance assessed in the present study provides a compelling justification for continued clinical development of P60.4Ac in therapy-resistant CSOM.

## Introduction

Otitis media is one of the most common bacterial infections. Acute otitis media is highly prevalent among infants and young children [1,2], whereas chronic suppurative otitis media (CSOM) is reported in patients of all ages. Although CSOM is a multifactorial disease, microbial virulence factors including toxins and cell wall constituents such as lipopolysaccharide (LPS), peptidoglycan (PG) and lipoteichoic acid (LTA), are thought to be important in its pathogenesis. Whereas bacterial toxins may directly damage the mucosal cells [3,4], they also trigger an inflammatory reaction that may injure the mucosa of the middle ear [5,6]. This mucosal injury results in an impairment of the mucociliary clearance system and inability to restore healthy equilibrium. Global estimates revealed that the chronic otitis media incidence rate worldwide amounts to 0.476%, i.e. approximately 31 million cases annually [7]. More importantly, CSOM causes hearing loss and results in decreased quality of life. In rare cases CSOM may even lead to life-threatening complications, such as brain abscesses [1].

Systemic and/or local antibiotics and/or anti-inflammatory agents (group III steroids) are often prescribed to patients with CSOM. Unfortunately, the efficacy of the first-choice antibiotics is increasingly jeopardized by the emergence of resistant bacterial strains [8]. Furthermore, the contribution of biofilms to the lack of therapeutic success in middle ear infections has been demonstrated in otitis media with effusion and direct biopsy specimens of the middle ear mucosa [9]. These effects are linked to impairment of the defence mechanisms mediated by the endogenous immune systems and by antibiotics. For these reasons, it is clear that innovative therapies in CSOM are urgently needed.

Antimicrobial peptides (AMPs) such as the human cathelicidin LL-37 are interesting candidates for such novel treatments as they are highly effective against an array of bacteria regardless of whether the bacteria are multiplying, are resistant to antibiotics or are residing within a biofilm [10]. Interestingly, LL-37 strongly binds to LPS, PG and LTA and neutralizes their pro-inflammatory activity [11]. LL-37 also impacts cellular processes, including proliferation, immune induction, wound healing, cytokine release and leukocyte chemotaxis [12,13]. Although the recruitment of inflammatory cells to sites of microbial invasion by LL-37 may be beneficial during acute infection, it may be more desirable during chronic infections to suppress inflammation. For treatment of CSOM, the LL-37-derived synthetic peptide P60.4Ac with a very low chemotactic activity towards human neutrophils—as compared to LL-37—was developed [14]. *In vitro* this peptide displayed antibacterial and antibiofilm and bacterial cell wall constituents-neutralizing activities similar to LL-37. In addition, no signs of ototoxicity, skin irritation or lasting eye irritation were observed upon exposure to P60.4Ac. Moreover, repeated intravenous administration of the peptide to male and female rats at 8 mg/kg/day on 5 consecutive days did not result in clear peptide-related changes in clinical signs, body weight, haematology, clinical biochemistry and macroscopic findings [unpublished data]. In view of these facts, the primary objective of the present study was to assess whether P60.4Ac in ototopical drops can be safely applied to the inflamed mucosa of the middle ear of adults with therapy-resistant CSOM. The secondary objective of this study was to assess the efficacy of P60.4Ac in ototopical drops in improving the mucosal aspect of the middle ear of adults with CSOM without cholesteatoma as compared to placebo. Our approach comprised a limited range-finding study and subsequently a double blind, placebo-controlled, phase II^a^ study of P60.4Ac in patients with CSOM without cholesteatoma who are resistant to current treatments.

## Materials and methods

### P60.4Ac synthesis and preparation of peptide-containing ototopical drops

N-terminal acetylated and C-terminal-amidated peptide P60.4Ac (Ac-IGKEF KRIVERIKRFLRELVRPLR-NH2; Mw 3094 Da [14]) was produced and analysed according to GMP standards in the peptide laboratory of the Interdivisional GMP facility of the LUMC. The purity of the peptide was >95%, as determined by reverse-phase high performance liquid chromatography. The purified peptide was lyophilized and then stored at -20°C. In the department of Clinical Pharmacy of the LUMC ototopical drops containing various concentrations (0.25 to 2.0 mg/ml) of the peptide were prepared from a single batch (03D040002) in high-density polyethylene eardrop bottles. The reconstitution fluid comprised of 50 mM sodium acetate buffer (pH 5.7) supplemented with 1 mg/ml EDTA, 0.2% (wt/v) benzalkonium chloride and 7% (v/v) PEG 10,000 in isotonic saline. The bottles with the reconstituted peptide and the reconstitution fluid alone (placebo) were stored at 4°C. The batch of P60.4AC-containing ototopical drops used in the phase II^a^ clinical study was 07A2273. This batch was released for clinical use by the qualified person at the Department of Clinical Pharmacy of the LUMC. The peptide-containing and the placebo ototopical drops were indistinguishable in appearance, odour, viscosity and packaging.

### Study population

Subjects were recruited from the coordinating center, i.e. the Ear Nose Throat Department of the LUMC (ENT/LUMC), and nine other Dutch Hospitals. Subjects were included in the study if they met the following criteria: age ≥18 year both males and females; legally competent; having a diagnosis of CSOM with chronic proliferative mucosal changes >6 months duration; a clear perforation of the tympanic membrane allowing proper inspection of the middle ear mucosa; antibiotic therapy resistant, defined as having received adequate treatment for CSOM for at least 2 periods of in total ≥ 6 weeks within the past year, with at least two different currently used ototopical drops and the last treatment period having occurred within the last 6 months before screening. The majority of the subjects (80%) had undergone surgery for chronic otitis media with limited success. Exclusion criteria were cholesteatoma or the presence of a radical cavity (both confirmed with CT-scan) in the ear to be treated; use of systemic immune suppressants or antibiotics, use of topical antibiotics, corticosteroids or other ototopical drops in one of the ears within 4 weeks before study start; Down’s syndrome or other congenital anomalies to the external ear canal, the middle ear or to the area of the pro-tympanum (i.e. where the Eustachian tube enters the middle ear) of the ear to be treated; presence of immune disorders; severe dizziness or severe headache, impacting on subjects’ daily activities; facial nerve disorders on the side of the ear to be treated; psychiatric history, pregnancy, the wish to become pregnant or to breastfeed during the study, or prior participation in the dose-finding study of the clinical development program. Finally, subjects could be withdrawn prematurely from the study in case of severe adverse events (SAEs) leading to hospitalization of the subject; sudden deafness or increasing sensorineural hearing loss; dizziness, facial nerve disorders or severe headache; subject’s request or non-compliance.

### Ethics

This study is listed on the ISRCTN registry with study ID ISRCTN2149720. Doi: 10.1186/ISRCTN12149720. The clinical study protocol (P02-216) and protocol amendments were approved by the Independent Ethics Committee (IEC) of the LUMC. The study was conducted in accordance with the Declaration of Helsinki. By signing the informed consent form, the subject consented to collection, handling and storage of these samples of body materials/fluids for the purposes of this study.

### Dose finding study

The dose finding was performed in 16 subjects in the ENT facilities of the LUMC. Subjects were allocated in order of inclusion to one of the four cohorts with ascending doses of P60.4Ac, i.e. 0.25, 0.5, 1.0 and 2.0 mg/ml. In short, a few drops (± 100 μl) were applied in the ear canal twice daily (in the morning and in the evening) by the subject or a person helping the subject at home for 2 weeks. The subjects were to lay down with the affected ear pointing upwards. The drops were applied in the affected ear, gently pulling the ear shell backwards and paying close attention to the bottle not touching the ear. The subjects were to remain in the same position for 3 min to allow the drops to set. The eardrop bottles containing the formulated peptide or placebo were stored at 4°C until 30 min before use. they were returned to the refrigerator immediately after use. The quantity of eardrops dispensed and returned was recorded by the LUMC pharmacist or his/her designee. The bottles were weighed before they were dispensed and after they were returned, in order to document drug use. Based on the results the compliance was excellent and all subjects completed the two-weeks treatment. Subjects were seen by the study physician in the clinic at screening (week 0) and in weeks 1, 2, 4, 8 and 12 for evaluation of the safety and efficacy of the P60.4Ac-containing ototopical drops.

### Safety and efficacy study

The randomized, double blinded, placebo-controlled, multicenter phase I/II^a^ study was prepared to run at 10 centers in the Netherlands with the coordinating center being the ENT of the LUMC. The study was blinded to the investigators and other medical personnel involved in the treatment of the subjects. Subjects were randomized using a randomization list (prepared by the statistician before study start) to be treated with ototopical drops containing the peptide (0.5 mg of P60.4Ac/ml) or consisting of vehicle only (placebo). Application of the ototopical drops and evaluation of the safety and efficacy was identical to the dose-finding study as described above. The randomization list was sent electronically to the LUMC pharmacy, responsible for the distribution of study drug. Randomization was done stratified by centre, via a balanced block design. Sample size calculations were performed with the software package East (version 3.1.0), which offers the possibility of interim analysis with an alpha and beta spending function. It was assumed that the otoscopically assessed success rate in the P60.4Ac group would be 60% and in the placebo group 20% and the difference was to be detected with a power of 90% at a significance level alpha of 5% in a two-sided test. Using the approach of the O’Brien-Fleming boundaries in the context of alpha and beta spending, the study would have the power to show both efficacy and futility, and it was calculated that the maximum number of subjects to be included was 52, i.e. two groups of 26 subjects each. According to protocol a formal interim analysis was scheduled after 26 subjects had completed the week 12 visit. For an interim analysis performed on the data of 26 subjects, it was calculated that there was a chance of 16% that the study was terminated because of ‘no difference’ between groups, while the efficacy was indeed the same in both groups. Likewise, it was calculated that there was a chance of 27% to terminate the study because of a significant difference between groups, while the difference was indeed 60% versus 20%.

The study was to be terminated in case of:

1. Safety. In ≥ 4 subjects one or more of the following events had occurred: a severe adverse event leading to hospitalization of the subject, sudden deafness or increasing perceptive hearing loss, dizziness, facial nerve disorders or severe headache.
2. Efficacy. The null hypothesis, that there is no difference between the two groups, could be rejected (efficacy), or the alternative hypothesis, that there is a difference between the two groups, could be rejected (futility).

If the formal termination criteria for efficacy were met, further continuation of the study was discontinued. The date of first enrolment was September 19^th^ 2006 and the date of last completed was July 4^th^ 2008.

### Evaluation of safety

To establish safety, the following assessments were made: recording of AEs and concomitant medication, laboratory tests on blood samples (specific antibodies directed against P60.4Ac peptide and general hematology), swabs from the middle ear and throat for bacterial culture. At screening and at weeks 1, 2, 4, 8 and 12, pure tone audiometry (PTA) and speech audiometry were performed. In addition, high-frequency pure tone audiometry (HFPTA) audiometry was done at screening and at week 12. The PTA and HFPTA were performed in the out-patient clinic by an audiometry assistant. At screening and at week 8, blood samples were drawn for the determination of specific antibodies directed against P60.4Ac at Covance Laboratories Ltd. (Harrogate, United Kingdom) and for the determination of hemoglobin, hematocrit, erythrocyte sedimentation rate and white blood cell differentials. These tests were performed locally.

### Evaluation of efficacy

The treatment was regarded a success when the mucosa of the middle ear was flat and dry (score = 0) or when an improvement of ≥2 points was observed at blinded otoscopic inspection using the following scores:

0: flat, dry mucosa of the middle ear
1: flat, discharging mucosa of the middle ear
2: thickened, polypoid, dry or discharging mucosa of the middle ear
3: thickened, polypoid middle ear mucosa with viscous mucous discharge.

In addition, the following quality of life (QoL) questionnaires were completed: the SF-36, the chronic ears survey (CES) including one additional study specific question (i.e. “Do you think the treatment was effective?”) and the Brief Illness Perception Questionnaire (IPQ-b).

### Statistical analyses

All data were listed by subject and summarized by means of descriptive statistics including means, standard deviations, medians and ranges, or frequencies and percentages as appropriate. The percentage of subjects with treatment success at end of the study was compared between groups by means of a Fisher exact test. In addition, a repeated measurement analysis of variance was performed using the outcome score as the dependent variable and time (baseline to week 12) as the independent covariate. As a very close analogue, and without the need to assume a specific covariance structure, a regression line was fitted per subject after which the slope and intercept of those lines were compared between the treatment groups.

SF-36 QoL data were analyzed by comparing both the average individual scores and the sum scores between groups. Statistical comparison was done in exactly the same way as for the primary efficacy variable: by taking the (domain) scores as the dependent variable and time as the independent covariate, and by subsequently fitting a regression line per subject over time and comparing the (average) slopes. For the IPQ-b questionnaire and the one question added to the CES, the analysis was limited to a comparison between groups of the week 12 results. The other CES data were not analyzed.

## Results

### Dose finding study

#### Patient characteristics

Sixteen subjects (9 females/7 males) were included with an average age of 52 years. All subjects suffered from a continuous ‘running ear’, despite the use of local and/or systemic antibiotics. Eight patients had an otoscopic score of 3 at baseline, 6 patients had a score of 2, and 2 patients had a score of 1. The average duration of their ear problems was 20 years. See Table 1 for demographics and baseline characteristics.

**Table 1:** Demographic data and other baseline characteristics of subjects in the dose finding study.

	(0.25 mg/ml)	(0.5 mg/ml)	(1.0 mg/ml)	(2.0 mg/ml)
**Number of subjects**	4	4	4	4
**Age** (mean years ± SD)	33 ± 15	69 ± 7	69 ± 9	38 ± 13
**Gender**				
Male n (%)	2 (50%)	2 (50%)	1 (25%)	2 (50%)
Female n (%)	2 (50%)	2 (50%)	3 (75%)	2 (50%)
**Duration of CSOM** (years ± SD)	24 ± 4	24 ± 20	13 ± 8	17 ± 9

#### Safety

The ototopical drops containing P60.4Ac appeared to be safe and well-tolerated at all four doses tested (results not shown). Adverse events were mild and no serious events were reported during the study. No negative effects on hearing levels were observed. In none of the subjects could antibodies against the peptides be detected 8 weeks after the start of the treatment. No changes in the presence of micro-organisms in the ear or throat were detected.

#### Efficacy

Overall 7 out of 16 patients had a dry ear with normal middle ear mucosa after 12 weeks. In addition, 5 subjects were slightly improved, 3 showed no change and 1 subject’s condition was slightly worsened. The perforation in the eardrum remained in all cases. The success rate (i.e. the number of patients having a dry ear) of the 2 lowest dosages at week 8 was 75% (Table 2). Ten weeks after treatment, the success rate was 50% for the 3 lowest doses. In the highest dose group the treatment was successful in only 1 subject. It should be noted that this subject used antibiotics during the follow-up period and was excluded from efficacy analysis. A linear mixed model (dependent = score; independents are dose groups and time) was used to fit the results from the 4-points scale, which was used to quantify the results from the mucosal thickness scores (S1 Fig). A quadratic model for the relation between the peptide dose and the score on the 4-points scale was chosen as starting-point. It was then determined whether the quadratic factor was significant. As this was not the case in the given area of examined peptide dose range no optimum could be estimated. However, the linear factor was significant and a clinically relevant difference in favour of the 2 lowest peptide doses compared to 1.0 and 2.0 mg/ml was found. Therefore, on clinical grounds the 0.5 mg of P60.4Ac/ml dose was selected for the double-blind, placebo-controlled phase II^a^ study of the safety and efficacy of P60.4Ac in CSOM.

**Table 2:** Individual response data of mucosal thickness during treatment and follow-up period in the dose finding study.

Subject number	Received dose P60.4Ac (mg/ml)	Mucosal thickness score during treatment and follow-up period (week)^a^ 0 1 2 4 8 12					
01	0.25	2	1	1	0	0	1 ^b^
02	0.25	2	0	1 ^b^	0	0	0
03	0.25	3	1	1	1	0	0
04	0.25	3	2	1	1	2	2
05	0.5	3	1	1	2	2	2
06	0.5	1	1	0	0	0	0
07	0.5	2	2	1	0	0	0
08	0.5	1	1	2	1	0	1
09	1.0	3	1	0	0	0	0
10	1.0	3	2	2	1	0	0
11	1.0	3	2	2	3	2	2
12	1.0	3	2	2	2	3	2
13	2.0	2	1	2	2	2	2
14	2.0	2	2	2	2	1	3
15	2.0	3	3	2	1	2	0^c^
16	2.0	2	1	1	3	2	2

^a^ Scores: 0: flat, dry mucosa; 1: flat, discharging mucosa; 2: thickened, polypoid, dry or discharging mucosa; 3: thickened polypoid tissue with viscous mucous discharge.

^b^ Subject had a cold at that time.

^c^ Subject had used an antibiotic treatment for frontal sinusitis.

### Safety and efficacy phase II^a^ study

#### Patient characteristics

**Fig 1:**
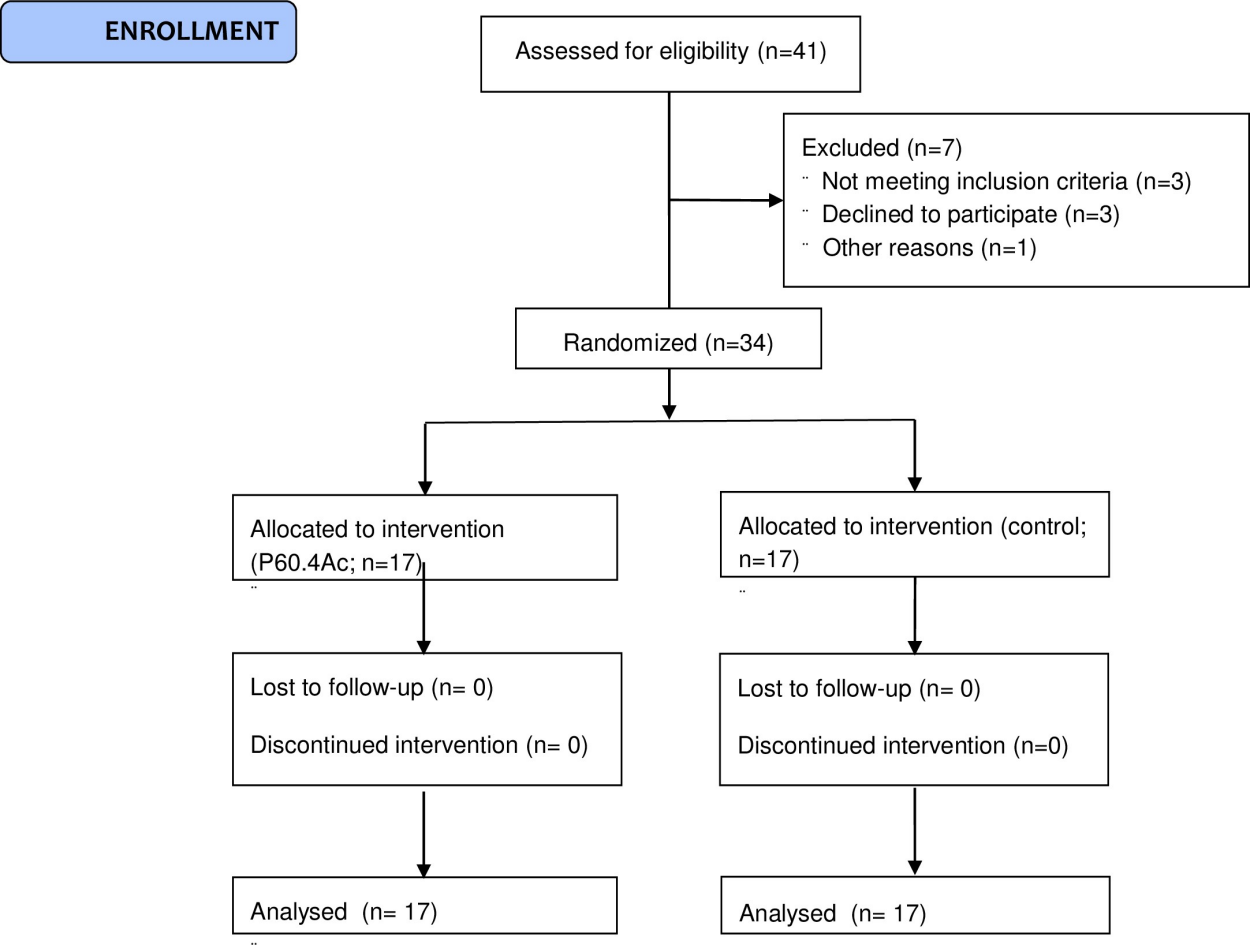
Flow diagram of the randomized, placebo-controlled study into the effects of ototopical drops containing the novel antimicrobial peptide P60.4Ac in 34 adults with chronic suppurative otitis media.

In total 41 CSOM patients were randomized in this study. Seven subjects were never included for the following reasons: Three subjects did not provide informed consent; three subjects did not meet the eligibility criteria and one subject had a radius fracture after randomization and before treatment. The event was considered an SAE because the subject was hospitalized for treatment of the fracture. Subsequently, subject did no longer meet the eligibility criteria and was not included in the study. Therefore, 34 subjects were included in this study. The demographic data are summarized in Table 3. There were no apparent differences between treatment groups with respect to age and severity of CSOM and duration of disease. The majority (71%) of the subjects entered the study with an otoscopic score of 3, 12% had a score of 2, and 17% had a score of 1. When the interim analysis was performed, the week 12 data of 30 subjects were available and, because formal termination criteria for efficacy were met on interim analysis, further participation in the study was stopped. According to protocol, the interim analysis was regarded as the final efficacy analysis. The 4 subjects, who were already in the study at the time the interim analysis was performed, completed the study. These data were included in the final safety and efficacy analyses. Together, 34 subjects were included in the safety and confirmatory efficacy analyses, 17 in each treatment group (Fig 1).

**Table 3:** Demographic data and other baseline characteristics of subjects in randomized double-blind, placebo-controlled phase II^a^ study.

	P60.4Ac (0.5 mg/ml)	Placebo	Total
**Number of subjects**	17	17	34
**Age** (mean years ± SD)	65 ± 3	65 ± 3	65 ± 2
**Gender**			
Male n (%)	9 (53%)	15 (88%)	24 (71%)
Female n (%)	8 (47%)	2 (12%)	10 (29%)
**Duration CSOM** (years ± SD)	21 ± 5	15 ± 4	18 ± 3
Range in years	1–60	1–50	1–60

#### Safety

None of the subjects discontinued the study treatment because of an (S)AE. A similar frequency of AEs was reported in the 2 treatment groups. Ten subjects of the P60.4Ac group (59%; 95% confidence intervals: 36–82%) and 16 subjects of the placebo group (81%; 95% confidence intervals: 64–100%) experienced in total 33 AEs and in 2 (12%; 95% confidence intervals: 0–27%) and 4 (24%; 95% confidence intervals: 4–44%) subjects, respectively, one of these events was considered (possibly or probably) treatment-related. Similar AEs were reported for the two treatment groups in similar frequencies (Table 4). Infections and infestations were the most frequently occurring adverse events (33% of the events) followed by headache (15%) and otalgia (9%). There were 6 study treatment-related AEs; infections and infestations affecting the middle ear, ear canal stenosis, and headache. All related AEs were reported for 1 subject each and all had occurred during weeks 1 and 2 of the study, except for ear canal stenosis which was diagnosed in week 12. All but 3 AEs were of mild to moderate intensity. In total 3 subjects experienced one serious adverse event (SAE) each, 1 subject in the P60.4Ac group (cardiac arrhythmia) and 2 subjects in the placebo group (food poisoning and transient ischemic attack). All SAEs had occurred >28 days after the last study drug administration and were considered unlikely to be related to the study drug. Furthermore, the use of concomitant medication was not considered to affect the end-points of the study.

**Table 4:** Incidence of adverse events by treatment group and total in randomized double-blind, placebo-controlled study.

System Organ Class	P60.4Ac	Placebo	Total
Preferred Term	n (%)	n (%)	n (%)
**Cardiac disorders**	**1 (6%)**	**0 (0%)**	**1 (3%)**
Arrhythmia	1 (6%)^a^	0 (0%)	1 (3%)
**Ear and labyrinth disorders**	**1 (6%)**	**4 (25%)**	**5 (15%)**
Ear canal stenosis	0 (0%)	1 (6%)^b^	1 (3%)
Otolgia (ear pain)	1 (6%)	2 (13%)	3 (9%)
Tinitis	0 (0%)	1 (6%)	1 (3%)
**Eye disorders**	**1 (6%)**	**1 (6%)**	**2 (6%)**
Conjunctivitis	0 (0%)	1 (6%)	1 (3%)
Eye inflammation	1 (6%)	0 (0%)	1 (3%)
**Gastrointestinal disorders**	**0 (0%)**	**1 (6%)**	**1 (3%)**
Food poisoning	1 (6%)	1 (6%)^a^	1 (3%)
**Infections and infestations**	**8 (47%)**	**3 (19%)**	**11 (33%)**
Bronchitis viral	1 (6%)	0 (0%)	1 (3%)
Erysipelas	0 (0%)	1 (6%)	1 (3%)
Gastroenteritis viral	1 (6%)	0 (0%)	1 (3%)
Nasopharyngitis	1 (6%)	0 (0%)	1 (3%)
Otitis externa	2 (12%)	0 (0%)	2 (6%)
Otitis media	2 (12%)^b^	1 (6%)^b^	3 (9%)
Otitis media acute	0 (0%)	1 (6%)^b^	1 (3%)
Sinusitis	1 (6%)	0 (0%)	1 (3%)
**Investigations**	**2 (12%)**	**2 (13%)**	**4 (12%)**
Biopsy	1 (6%)	0 (0%)	1 (3%)
Blood bilirubin increased	0 (0%)	1 (6%)	1 (3%)
Red blood cell sedimentation rate increased	1 (6%)	1 (6%)	2 (6%)
**Nervous system disorders**	**2 (12%)**	**3 (19%)**	**5 (15%)**
Convulsion	0 (0%)	1 (6%)	1 (3%)
Headache	2 (12%)	1 (6%)^b^	3 (9%)
Transient ischaemic attack	0 (0%)	1 (6%)^a^	1 (6%)
**Respiratory disorders**	**2 (12%)**	**0 (0%)**	**2 (6%)**
Dyspnoea	1 (6%)	0 (0%)	1 (3%)
Pharyngolaryngeal pain	1 (6%)	0 (0%)	1 (3%)
**Skin and subcutaneous disorders**	**0 (0%)**	**2 (13%)**	**2 (6%)**
Erythema	0 (0%)	1 (6%)	1 (3%)
Excessive granulation tissue	0 (0%)	1 (6%)	1 (3%)
**Total**	**17**	**16**	**33**
**Any subject with AE**	**10 (59%)**	**13 (81%)**	**23 (70%)**

^**a**^ Event reported as serious adverse event (SAE)

^b^ Event reported as treatment-related event

Totals per system organ class are presented in **bold**

Small clinically-insignificant changes from baseline were observed in haematology parameters (S1 Table). Variable decreases in mean neutrophil and lymphocyte counts were observed in the P60.4Ac treatment group. A frequency table of the microorganisms found at baseline and after 12 weeks, using middle ear swabs is presented in S2 Table. There were, however, no statistically significant differences between groups with respect to any of the safety parameters analysed. There were no other findings of clinical relevance with respect to laboratory assessments or other physical examinations, including audiometry.

#### Efficacy

**Fig 2:**
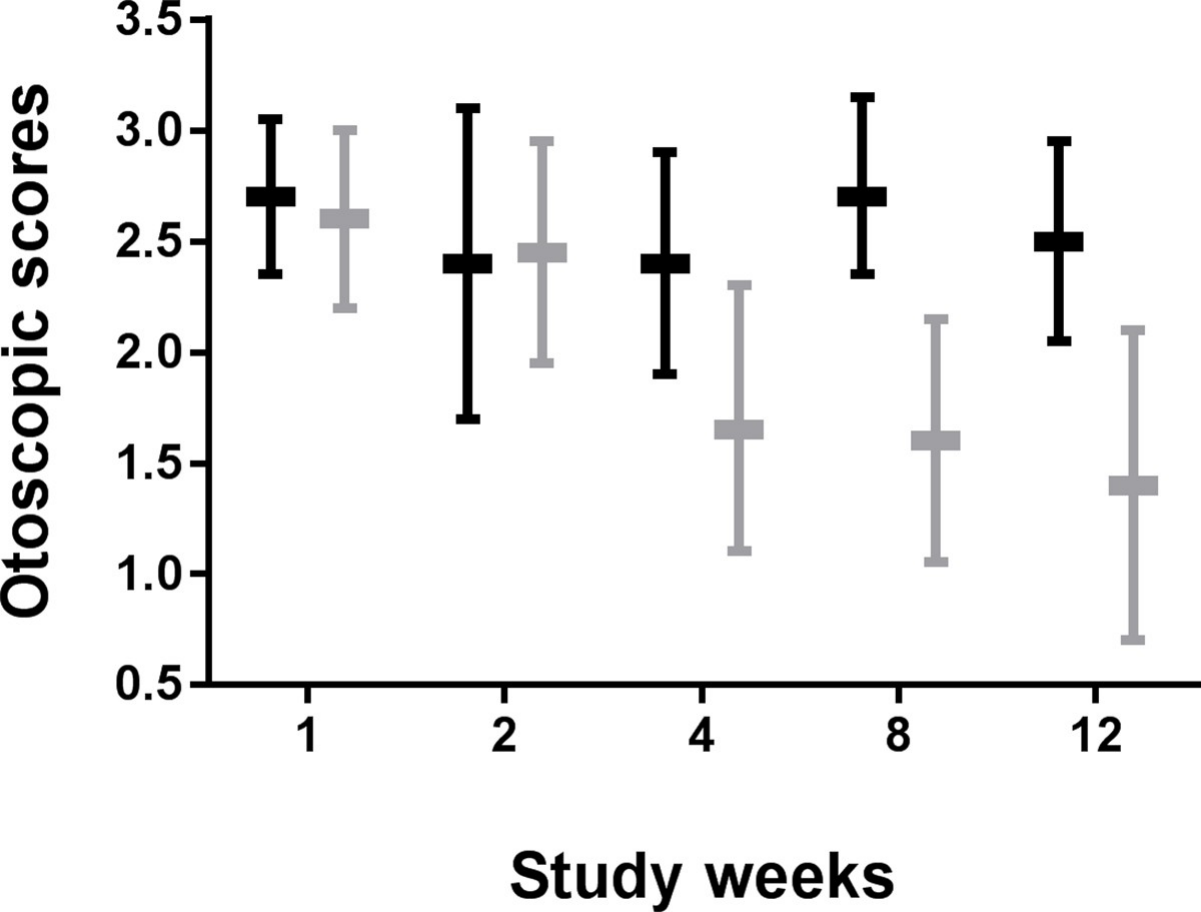
Mean otoscopic scores and 95% confidence intervals in time for placebo (gray bars) and P60.4Ac-containing (black bars) ototopical drops.

The distribution of baseline otoscopic scores was the same in the two treatment groups, both resulting in 71% of the subjects having a score of 3 and none of the subjects having a score of 0 at baseline. From week 4 onwards, a significant difference in score distribution was apparent between treatment groups, with 32% to 53% of the subjects in the P60.4Ac group having a score of 0 or 1 at week 4 and week 12, respectively, while this was the case in only 18–19% of the subjects in the placebo group at weeks 4 and 12 (Table 5). At week 12 treatment was effective in 47% of the subjects in the P60.4Ac group, while this was the case for only 6% of the subjects in the placebo group (intention to treat analysis, p = 0.017). Following a per protocol analysis, active treatment was effective in 56% of the subjects and in none of the subjects in the placebo group (p = 0.012). The mean otoscopic scores and 95% confidence intervals for both groups in time are presented in Fig 2. A statistically significant (-0.11; confidence interval -0.18 - -0.04) difference was found between the P60.4Ac and the placebo group with respect to the slope of the scores at the different visits, implying a faster response to active treatment compared to placebo.

**Table 5:** Distribution of the ototopic scores for therapy resistant CSOM patients treated with P60.4Ac-containing ototopical drops and placebo drops during treatment and follow-up.

		P60.4Ac	Placebo
Visit	Score	n (%)	n (%)
Week 0	3	12 (71)	12 (71)
	2	2 (12)	2 (12)
	1	3 (18)	3 (18)
	0	0 (0)	0 (0)
Week 4	3	3^a^ (19)^a^	10 (59)
	2	8^a^ (50)^a^	4 (24)
	1	3^a^ (19)^a^	2 (12)
	0	2^a^ (13)^a^	1 (6)
Week 8	3	3 (18)	12 (971)
	2	6 (35)	3 (18)
	1	5 (29)	2 (12)
	0	3 (18)	0 (0)
Week 12	3	4 (24)	10^a^ (63)
	2	4 (24)	3^a^ (19)
	1	3 (18)	3^a^ (19)
	0	6 (35)	0^a^ (0)

Results are otoscopic scores at visit. Each group comprised 17 patients.

^a^ Data for 1 subject is missing. Scores: 3: thickened, polypoid mucosa with viscous mucous discharge; 2: thickened, polypoid, dry or discharging mucosa; 1: flat, discharging mucosa; 0: flat, dry mucosa.

The SF-36 and IPQ-b analyses showed no relation between the various scores in QoL and the treatment groups or correlation between QoL scores and the efficacy parameters themselves. Since the study had not been powered to detect differences in QoL scores, this outcome probably suggests that the sample size was too small to evaluate effects on QoL. Only the single question added to the CES “Do you think the treatment was effective” reached statistical significance (p = 0.024) in favour of the experimental arm. In this study therapy-resistant patients were selected and therefore, no comparator has been included in the study. However, a cost-benefit analysis comparing the P60.4Ac-containing ear drops and currently used ear drops (group III steroids plus topical antibiotics) or surgery in CSOM patients could be the next step in the introduction of the peptide for CSOM.

Major protocol deviations had occurred in 8 subjects leading to the exclusion of these subjects from the per protocol (PP) analysis. Three subjects were excluded from the PP analysis because they had a major deviation from the eligibility criteria. Five subjects were excluded because they received concurrent antibiotic treatment. In total 5 subjects in the P60.4Ac group and 3 subjects in the placebo group had major protocol deviations. The mean difference between the P60.4Ac and the placebo group with respect to the slope of the scores at the different visits in the ‘intention-to-treat’ group and the PP group was identical (-0.11) and this difference was significant in both groups.

## Discussion

Results of the presently double-blinded, phase II^a^ study demonstrate that P60.4Ac-containing ototopical drops—applied twice daily in the ear canal for 2 weeks—are safe, well-tolerated and efficacious in adults with therapy-resistant CSOM. This conclusion is based on the following findings. First, similar adverse events (AEs), in the same frequencies, were found for the P60.4Ac and the placebo groups, indicating the absence of peptide-related AEs. Most AEs were disease-related and consisted of middle ear disorders or infections and infestations. Six AEs were considered study treatment-related, two AEs in the P60.4Ac group and four AEs in the placebo group. The three reported SAEs (one in the P60.4Ac group) were unlikely related to treatment and there were no SAEs within 28 days after the last drug application. No antibodies to P60.4Ac had developed 8 weeks after the last application and there were no other findings of clinical relevance with respect to laboratory assessments or physical examinations, including audiometry. Secondly, a statistically significant higher rate of treatment success was achieved in the P60.4Ac group than in the placebo group: 47% versus 6% (p = 0.017). The slopes of the otoscopic scores *versus* time were also statistically significantly different between treatment groups, i.e. the speed of improvement in otoscopic scores over time was faster in the P60.4Ac group compared to placebo. This is further exemplified by the results of the pre-specified interim analysis, which was planned to be performed as soon as 26 subjects had completed the week 12 visit. At the time of this analysis, data from 30 subjects were available. Since the formal criteria for stopping the trial due to efficacy of the treatment were met, continuation of the study was stopped in accordance with the protocol. As such, the data of these 30 subjects together with the data from 4 additional subjects on-going at the time the interim analysis was performed, were regarded as the final efficacy analysis. Furthermore, the dose-finding study indicated that ototopical drops containing 0.5 mg of P60.4Ac/ml were more effective than drops containing the two highest concentrations (1 and 2 mg of peptide/ml). This is in agreement with a recent report on the efficacy of LL-37 in enhancing wound healing in patients with venous leg ulcers showing that 0.5 mg/ml was slightly more effective than 1.6 mg/ml and much more effective than 3.2 mg/ml; all three doses of LL-37 were safe and well-tolerated [15]. Nevertheless, a few important questions remain to be answered. For example, why is treatment with P60.4Ac-containing ototopical drops successful in only 47% of the CSOM patients? It is likely that in many patients the peptide may not have reached the middle ear mucosa due to the presence of viscous mucosal discharge. At the start of the study start it was decided that manipulation of the ear should be avoided in order to reduce the risk of introducing a new infectious agent into the middle ear. For a future study it is therefore recommended to clean out the ears thoroughly before applying the ototopical drops. In addition, treatment may be repeated if not successful 6 weeks after treatment start. Another question concerns the mode of action of P60.4Ac in CSOM. Whereas it is anticipated that the antibacterial and anti-biofilm activities of the peptide [14,16,17] contributed to the effect, no major changes in microbial flora of the mucosa of the middle ear of the patients were observed. Another possibility is that the peptide neutralizes the cytotoxic and pro-inflammatory effects of bacterial products [14] such as lipopolysaccharides and lipoteichoic acid, thus reducing damage and inflammation of the membrane of the middle ear. At this stage no definitive explanation for the clinical success of P60.4Ac in ototopical drops in CSOM patients can be provided. The design of this study provided a robust approach to establishing the safety and efficacy of a novel treatment for CSOM. Another major advantage of this study is that it was performed in subjects with CSOM in whom current therapies were completely ineffective and who had a long history of otologic symptoms. It is to be noted that CSOM patients typically have a perforation of the tympanic membrane allowing direct access of the ototopical drop to the middle ear. Therefore, the safety assessment in patients was considered as more relevant than in healthy subjects with an intact tympanic membrane. The treatment groups were similar with respect to age and duration of disease. However, there were more males in the placebo group, i.e. 88% males versus 12% females, than in the treatment group, with 53% males versus 47% females. Although this finding should be kept in mind, there is at present no reason to assume that this disbalance has in any way affected the results.

The design of the present phase IIa study did not allow a further evaluation of therapy failure in individual patients. Also, it is to be noted that the blinded scoring at otoscopic inspection was performed by the investigators, and was therefore subject to between-observer variation. In addition, the scores were not validated. To circumvent these limitations, video otoscopy and centralized blinded assessment are recommended for future trials. In addition, the small numbers of subjects included in the two arms of the study (which was due to pre-planned cessation of the study based on the results of the interim analysis) is a limitation of the study in terms of building a large safety data base. As the study was underpowered to detect QoL differences between treatments with statistical significance, no meaningful conclusions with respect to the QoL scores could be drawn. The single question that was added to the CES questionnaire “Do you think the treatment was effective?” did reach statistical significance (p = 0.024) in favor of the experimental arm. However, since this is not a validated question of the CES, the significance of this finding remains to be confirmed. The primary efficacy outcome was originally planned to be analysis by means of repeated measurement analysis of variance with the zero measurement as covariate, in addition to non-parametric analysis of the rate of treatment success in the two treatment groups (Fisher Exact test). Several mixed models were fitted to compare the two treatment groups, assuming different covariance structures. Since the results from these different models were extremely close, the simpler regression approach was used, in which the outcome score was taken as the dependent variable and time as the independent covariate. In this study therapy-resistant patients were selected and therefore, no comparator has been included. However, a cost-benefit analysis comparing the P60.4Ac-containing ear drops and currently used ear drops (group III steroids plus topical antibiotics) or surgery in CSOM patients could be the next step in the introduction of the peptide for CSOM. The high medical need in CSOM, especially in treatment-resistant CSOM, implies that this innovative treatment has the potential to lead to a major improvement of patient management and a reduction in treatment costs in ENT units. Moreover, this approach has the important possibility of providing a relief in cases of recurrence. Together, the results of this proof-of-concept study clearly shows that synthetic antimicrobial peptides can be promising candidates for the development of novel agents to treat chronic otitis media. Furthermore, the data indicate that such peptides hold promise as agents to combat infections that are not effectively treatable with current antibiotics, such as infections caused by multidrug resistant bacteria and recurrent, hard-to-treat infections.

## Supporting information

S1 Checklist CONSORT 2010 checklist of information to include when reporting a randomised trial*. (PDF)

S1 Table Changes from baseline in hematology parameters at week 8. (DOCX)

S2 Table Microorganisms in bacterial culture from middle ear swabs at baseline and week 12. (DOCX)

S1 Fig (TIF)

S1 Protocol (PDF)

S2 Protocol (DOCX)
