# Supplementary material for: Ototopical drops containing a novel antibacterial synthetic peptide: Safety and efficacy in adults with chronic suppurative otitis media
Source: PLoS One. 2020 Apr 14;15(4):e0231573. doi: 10.1371/journal.pone.0231573 (PMC7156094; doi:10.1371/journal.pone.0231573)
Supplement: S1 Table — (DOCX) [file pone.0231573.s002.docx]

Supplementary Table 1. Changes from baseline in hematology parameters at Week 8.

| Parameter | Statistics | P60.4Ac | Placebo |
| --- | --- | --- | --- |
| Hemoglobin (mmol/L) | N | 17 | 17 |
|  | Mean difference ± SD | -0.60 ± 2.092 | 0.49 ± 2.131 |
|  | SEM | 0.507 | 0.517 |
| Hematocrit (fraction) | N | 17 | 17 |
|  | Mean difference ± SD | -0.27 ± 0.101 | 0.02 ± 0.101 |
|  | SEM | 0.024 | 0.025 |
| Erythrocytes (10^12^/L) | N | 12 | 13 |
|  | Mean difference ± SD | 0.85 ± 2.037 | 0.70 ± 1.747 |
|  | SEM | 0.588 | 0.485 |
| Leukocytes (10^9^/L) | N | 17 | 17 |
|  | Mean difference ± SD | -0.22 ± 2.214 | 0.22 ± 1.822 |
|  | SEM | 0.537 | 0.442 |
| Eosinophils (%) | N | 12 | 8 |
|  | Mean difference ± SD | -0.67 ± 2.022 | 0.53 ± 1.127 |
|  | SEM | 0.584 | 0.399 |
| Basophils (%) | N | 12 | 8 |
|  | Mean difference ± SD | 0.13 ± 0.733 | 0.44 ± 1.134 |
|  | SEM | 0.211 | 0.401 |
| Neutrophils (%) | N | 12 | 8 |
|  | Mean difference ± SD | -11.2 ± 22.52 | -1.15 ± 4.763 |
|  | SEM | 6.502 | 1.684 |
| Lymphocytes (%) | N | 12 | 8 |
|  | Mean difference ± SD | -11.7 ± 28.71 | -0.04 ± 4.991 |
|  | SEM | 8.287 | 1.765 |
| Monocytes (%) | N | 12 | 8 |
|  | Mean difference ± SD | -1.11 ± 2.685 | 0.51 ± 1.795 |
|  | SEM | 0.775 | 0.635 |
| BSE (mm) | N | 12 | 13 |
|  | Mean difference ± SD | -1.58 ± 6.829 | 2.85 ± 9.564 |
|  | SEM | 1.971 | 2.653 |
