# Supplementary material for: Ototopical drops containing a novel antibacterial synthetic peptide: Safety and efficacy in adults with chronic suppurative otitis media
Source: PLoS One. 2020 Apr 14;15(4):e0231573. doi: 10.1371/journal.pone.0231573 (PMC7156094; doi:10.1371/journal.pone.0231573)
Supplement: S2 Table — (DOCX) [file pone.0231573.s003.docx]

Supplementary Table 2. Microorganisms in bacterial culture from

middle ear swabs at baseline and Week 12.

|  | **P60.4Ac** | | **Placebo** | |
| --- | --- | --- | --- | --- |
| **Microorganism** | **Baseline (n=17)** | **Week 12 (n=17)** | **Baseline (n=16)** | **Week 12 (n=16)** |
| None | 6 | 3 | 3 | 1 |
| Residential skin flora | 6 | 9 | 6 | 7 |
| Residential mixed flora | 1 | 2 | 1 | 2 |
| Fungi | 1 | 0 | 0 | 0 |
| Candida | 0 | 0 | 0 | 1 |
| *Candida parapsilosis* | 0 | 1 | 0 | 0 |
| *Staphylococcus aureus* | 3 | 2 | 4 | 1 |
| Staphylococcus coagulase negative | 0 | 0 | 0 | 1 |
| *Pseudomonas aeruginosa* | 2 | 1 | 2 | 4 |
| *Pseudomonas putida* | 1 | 0 | 0 | 0 |
| *Serratia marcescens* | 1 | 1 | 0 | 1 |
| Proteus | 0 | 1 | 0 | 0 |
| *Proteus mirabilis* | 2 | 1 | 2 | 2 |
| *Enterobacter aerogenes* | 1 | 0 | 0 | 0 |
| *Enterobacter cloacae* | 0 | 1 | 0 | 0 |
| *Klebsiella pneumoniae* | 0 | 0 | 2 | 2 |
| *Klebsiella oxytoca* | 0 | 0 | 1 | 0 |
| Gram neg. rods | 0 | 2 | 0 | 0 |
| ß-haemolytic streptococcen group G | 0 | 0 | 0 | 2 |
| *Stenothrophomonas maltophilia* | 0 | 0 | 1 | 0 |
| Citrobacter | 0 | 0 | 0 | 1 |
| *Citrobacter braakii* | 0 | 0 | 1 | 0 |
| *Achromobacter xylosoxidans* | 0 | 0 | 0 | 1 |
