# Supplementary figures and images for: Ototopical drops containing a novel antibacterial synthetic peptide: Safety and efficacy in adults with chronic suppurative otitis media
Source: PLoS One. 2020 Apr 14;15(4):e0231573. doi: 10.1371/journal.pone.0231573 (PMC7156094; doi:10.1371/journal.pone.0231573)

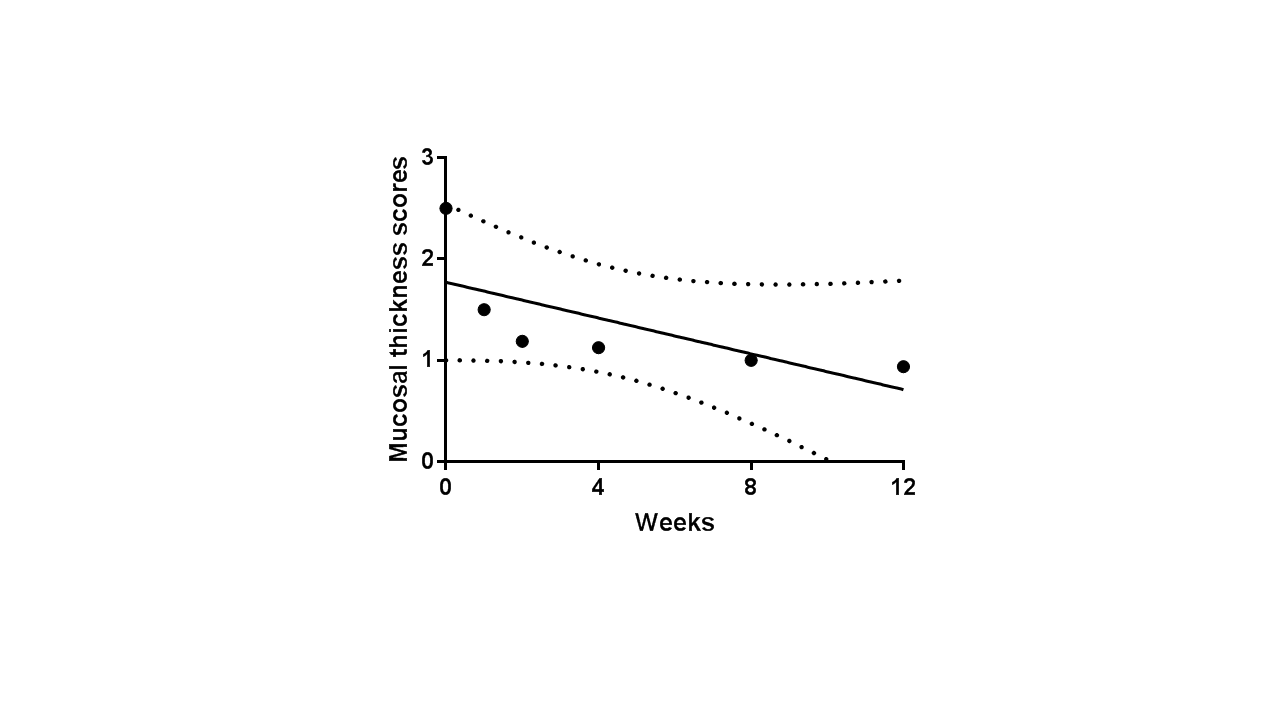

Supplement: S1 Fig — (TIF) [file pone.0231573.s004.tif]
