# Supplementary material for: Ototopical drops containing a novel antibacterial synthetic peptide: Safety and efficacy in adults with chronic suppurative otitis media
Source: PLoS One. 2020 Apr 14;15(4):e0231573. doi: 10.1371/journal.pone.0231573 (PMC7156094; doi:10.1371/journal.pone.0231573)
Supplement: S1 Protocol — (PDF) [file pone.0231573.s005.pdf]

16.0 APPENDICES

16.1 STUDY INFORMATION

16.1.1 Protocol and protocol amendments

Section 16.1.1 consists of 114 pages that are separately numbered

Titel:

Behandeling van chronische middenoorontsteking bij volwassenen

met behulp van het antimicrobieel peptide AMP60.4Ac

**Verrichter:**	Leids Universitair Medisch Centrum<br>Albinusdreef 2, 2333 ZA, Leiden
**Uitvoerder:**	Drs. F.A.W. Peek<br>Afdeling Keel- Neus- en Oorheelkunde<br>Leids Universitair Medisch Centrum
**Statisticus:**	Dr. R. Brand<br>Medische Statistiek
**Andere betrokkenen:**	Dr. J.W. Drijfhout<br>Immunohematologie en Bloedtransfusie (IHB)<br><br>Dr. P.S. Hiemstra<br>Longziekten<br><br>Drs. G. Slappendel<br>Klinische Farmacie en Toxicologie<br><br>Academisch Medisch Centrum, Amsterdam<br><br>Erasmus Universitair Medisch Centrum, Rotterdam
**Participerende Instelling:**	OctoPlus Technologies BV.<br>Zernikedreef 12<br>2333 CL Leiden
**Financiering:**	LUMC en OctoPlus Technologies BV.
**Contactpersonen:**	Drs. F.A.W. Peek<br>Tel. 071-526 2434
**Onafhankelijk arts:**	Dr. A.G.L. van der Mey (stafarts KNO, LUMC)<br>Tel. 071-526 3404

1.1 Handtekeningenblad

De hieronder genoemde betrokkenen van het LUMC en de participerende instelling verklaren hierbij accoord te gaan met dit studieprotocol.

Drs. F.A.W. Peek
Hoofdonderzoeker

Dr. J.W. Drijfhout
Immunohematologie en Bloedtransfusie (IHB)

Prof. Dr. P.S. Hiemstra
Longziekten

Drs. G. Slappendel
Apotheker

1.2 Handtekeningenblad overige deelnemende centra

De hieronder genoemde betrokkenen van de overige deelnemde centra verklaren hierbij accoord te gaan met dit studieprotocol.

Prof. Dr. W. Fokkens (AMC, Amsterdam)

Dr. W. Grolman (AMC, Amsterdam)

Dr. K. H. Pauw (Erasmus UMC, Rotterdam)

Dr. J. Van Linge (Erasmus UMC, Rotterdam)

2. Inhoudsopgave

1.1 Handtekeningenblad 2

1.2 Handtekeningenblad overige deelnemende centra 3

2. Inhoudsopgave 4

3. Lijst met afkortingen 6

4. Samenvatting 6

5. Inleiding 7

5.1 Achtergrond 7
5.2 Huidige situatie 7
5.3 Pre-klinisch onderzoek 7
5.4 Onderzoeksproduct 8
5.5 Risico's en voordelen proefpersonen 8
5.6 Inbreng participerende instelling 9

6. Hypothese / Vraagstelling 9

7. Doelstelling 9

8. Onderzoekspopulatie 9

9. Onderzoeksopzet 10

9.1 Ontwerp 10
9.2 Onderzoeksbehandeling 11
9.3 Schematisch diagram 13
9.4 Behandelschema 14
9.5 Duur en omvang van het onderzoek 14

10. Eindpunten 15

10.1 Primaire en secundaire eindpunten: 15
10.2 Meetmethoden 15

11. Methode van meten 15

12. Lichaamsmaterialen 16

13. Methode van uitvoering / Laboratoriumwerkwijze 16

14. Statistische analyses 16

14.1 Statistische methoden *16*

14.2 Statistische analyse *17*

14.3 Aantal proefpersonen *17*

15. Uitkomsten/bevindingen en hun implicaties **18**

16. Ethische overwegingen **18**

17. Controle vordering van het onderzoek **18**

17.1 Beoordeling van de veiligheid *18*

17.2 Beëindiging van het onderzoek en (serious) adverse events (SAEs) *18*

17.3 Monitoring Committee *19*

17.4 Interim-analyses *19*

17.5 Studie formulieren en gegevens *22*

17.6 Afwijkingen van het protocol *22*

18. Publicaties **23**

19. Administratieve procedures en verantwoordelijkheden **23**

20. Verzekering **23**

21. Referenties **24**

Addenda:

Investigator's Brochure

Informed consent met toestemmingsverklaring

Case Report Forms:

1. Screening formulier
2. Controle bezoek formulier
3. Completion pagina
4. Serious Adverse Event (SAE) formulier

3. Lijst met afkortingen

AMP	Antimicrobieel peptide
BPI	Bactericidal permeability increasing protein
COM	Chronische Otitis Media
CME	Commissie Medische Ethiek
CRF	Case report form
ERK	Extracellular signal-related kinase
GCP	Good Clinical Practice
GMP	Good Manufacturing Practice
GNB	Gram-negatieve bacteriën
GPB	Gram-positieve bacteriën
LPS	Lipopolysaccharide
LTA	Lipoteichoid acid
MAP	Mitogen activated phoshate
MCS	Mucociliair clearance systeem
OME	Otitis media met effusie
SADR	Serious adverse drug related
SAE	Serious adverse event

4. Samenvatting

Deze studie heeft tot doel een nieuwe behandelingsmethode voor chronische middenoorontsteking bij volwassenen te testen op veiligheid en werkzaamheid. De nieuwe behandelingsmethode bestaat uit oordruppels met een antimicrobieel peptide. Door het groeiende probleem van bacterie resistentie voor antibiotica is er behoefte aan nieuwe veilige middelen voor de behandeling van bovenste luchtweg infecties. De hypothese in deze studie is dat de nieuwe behandeling resulteert in een verbetering van het middenoorslijmvlies ten opzichte van behandeling met oordruppels zonder antimicrobieel peptide. De studie is voorafgegaan door een dose-finding studie. Hierbij is de veiligheid getest en de optimale dosering van het peptide gezocht. De dubbelblind gerandomiseerde studie heeft tot doelstelling te onderzoeken of de oordruppels met het antimicrobieel peptide effectiever zijn dan placebo oordruppels. De onderzoekspopulatie voor deze studie betreft een groep volwassenen met langdurige middenoorproblemen (chronische otitis media (COM) en een trommelvliesperforatie), die geen baat hebben gehad van eerdere behandelingen met antibiotica. Voor de dose-finding studie zijn steeds 4 deelnemers per concentratie geïncludeerd en voor de hoofdstudie zullen totaal 52 patiënten worden geïncludeerd. De deelnemende proefpersonen zullen gedurende 2 weken 2 maal daags de oordruppels gebruiken. Met behulp van oorinspectie, een CT-scan van het mastoid, een toon-audiogram, bacteriekweek van oor en keel, en bloedanalyse zal de veiligheid en de werkzaamheid vastgelegd worden. Indien mogelijk zal bij aanvang middenoorvocht worden afgenomen voor aanvullend bacteriologisch onderzoek. De patiënten worden na inclusie 12 weken gevolgd. Als eindpunt geldt een herstel van het middenoorslijmvlies zonder secretie en een droge en rustige trommelvliesperforatie. De studie zal volgens GCP- richtlijnen worden uitgevoerd.

5. Inleiding

5.1 Achtergrond

In verband met toenemende antibiotica-resistentie van bacteriën[1,2] is het van groot belang te zoeken naar nieuwe verbeterde behandelingsmogelijkheden. Het door ons ontwikkelde onderzoeksproduct biedt een mogelijk alternatief voor het toenemende gebruik van antibiotica. Met deze gerandomiseerde studie willen we daarom het onderzoeksproduct na toxiciteit- en dosis/effect onderzoek bij volwassenen met middenoorproblemen onderzoeken.

Bijna 75% van alle jonge kinderen heeft minstens één keer middenoorontsteking met ophoping van vocht (effusie) in het middenoor veroorzaakt door chronisch veranderd slijmvlies en een verdikt mucus producerend slijmvlies. Dit gaat soms gepaard met een tijdelijk gehoorsverlies. Meestal gaat de inflammatie vanzelf over, maar als de effusie aanwezig blijft krijgt ongeveer 15% van deze kinderen een chronische middenoorontsteking (OME).[3,4] Hierbij kunnen langdurige gehoorproblemen ontstaan. Tevens kan hierdoor een blijvende schade aan het trommelvlies of de gehoorbeenketen ontstaan. De aandoening kan zich ook bij volwassenen voordoen, al komt dit minder vaak voor dan bij kinderen. Bij volwassenen wordt vaker chronisch veranderd slijmvlies gezien bij otitis media chronica samen met een perforatie van het trommelvlies. Ongeveer 2% van de populatie heeft significante gezondheidsproblemen door chronische otitis media.[5]

5.2 Huidige situatie

Tot op heden worden kinderen met middenoorontsteking vooral behandeld met trommelvliesbuisjes, antibiotica al of niet in combinatie met het verwijderen van de neusamandelen.[3] Nadelen van deze behandelingsmethoden zijn o.a. voortijdig verlies van de buisjes, schade aan het trommelvlies en ontstaan van resistente bacteriën.[6] Ook worden met deze behandelingsmethoden bacterieproducten niet geneutraliseerd zodat de ontstekingsreactie door kan gaan. Veel kinderen krijgen zelfs meer dan éénmalig plaatsing van trommelvliesbuisjes met het risico van een blijvende beschadiging aan het trommelvlies. In geval van succesvolle behandeling met antibiotica komen de klachten vaak binnen 30 dagen na beëindiging van de therapie terug.[7] Volwassenen worden vaak behandeld met antibiotica, en bij trommelvliesperforatie met locale antibiotica, maar in een deel van de patiënten zonder blijvend resultaat. Zelfs chirurgie geeft niet altijd verbetering.

5.3 Pre-klinisch onderzoek

Zowel de bacterieproducten lipopolysaccharide (LPS) als lipoteichoid acid (LTA) kunnen chronische veranderingen van mucosa induceren. Het is gebleken dat LPS een belangrijke rol speelt bij de pathogenese van chronische middenoorontsteking. LPS is aangetoond in middenoorvocht en is tevens in staat gebleken inflammatoire reacties in het middenoor en in de buis van eustachius op te wekken.[8,9,10,11] Wanneer de buis van eustachius slecht functioneert of verstopt is kan overtollig vocht niet meer uit het middenoor verwijderd worden. Dit gebeurt normaal gesproken door het mucociliaire clearance systeem (MCS), wat bestaat uit slijmbekercellen en trilhaar(dragende)cellen. Door beweging van de trilharen kan het slijm met daarin eventueel aanwezige bacteriën of bacterieproducten verwijderd worden uit het middenoor. Als door infectie en/of obstructie van de buis van eustachius het MCS ontregeld raakt blijven producten zoals LPS en LTA in het middenoor aanwezig en kunnen zo de ontstekingsreactie opnieuw induceren zodat een chronische OME en COM ontstaat. Zo ontstaat er een vicieuze cirkel. Om deze te doorbreken is in preklinisch onderzoek onderzocht of het antimicrobieel peptide AMP60.4Ac LPS en LTA kan neutraliseren.

Uit voorgaande studies binnen onze afdeling is gebleken dat neutralizatie van LPS zowel *in vitro* als *in vivo* met behulp van het LPS-neutralizerende bactericidal permeability increasing (BPI) eiwit resulteerde in een herstel van het MCS.[12,13] Effectieve neutralizatie van bacterieproducten ter plaatse biedt daarom mogelijkheden voor behandeling van chronische middenoorproblemen. Het nieuwe door ons ontwikkelde synthetische peptide is effectiever in LPS neutralizatie gebleken dan BPI en is ook werkzaam in de neutralizatie van LTA.

Het groeiende probleem van bacterie resistentie voor antibiotica en de behoefte aan nieuwe antibiotica heeft de interesse gewekt voor de ontwikkeling van antimicrobiële peptiden als humane therapeutica. Verschillende van deze peptiden, afgeleid van menselijke of dierlijke antimicrobiële eiwitten, worden op dit moment voor verschillende toepassingen getest in klinische studies.[14]

5.4 Onderzoeksproduct

AMP60.4Ac is een synthetisch peptide afgeleid van het humane cathelicidin LL-37. LL-37 is een cationisch eiwit aanwezig in neutrofiele granulocyten en epitheelcellen en wordt tevens uitgescheiden door onder andere het epitheel van de luchtwegen en de huid.[15,16] Naast een directe antimicrobiële functie, fungeert LL-37 als mediator van de inflammatie en heeft effecten op epitheel- en ontstekingscellen. Hierbij beïnvloedt het processen als proliferatie, immuun inductie, wond heling, cytokine release, mestcel activatie en chemotaxis.[18,19] LL-37 is betrokken bij de afweerrespons tegen gram-negatieve (GNB) en gram-positieve bacteriën (GPB).[20] Het bindt sterk aan zowel LPS van GNB als aan LTA van GPB en neutraliseert tevens hun pro-inflammatoire activiteit. In muizen leidt deficiëntie van het muizen cathelicidin CRAMP in een verminderde capaciteit om huidinfecties te controleren.[21] Bij de mens gaat een granulocyten stoornis (morbus Kostmann), die leidt tot LL-37 deficiëntie in speeksel, veelal gepaard met peridonditis.[17] LL-37 blijkt dus belangrijk bij de controle van onstekingsreacties. Tijdens inflammatie processen is er een verhoogde LL-37 expressie aangetoond.[22,23] Het werkingsmechanisme, de immunotoxiciteit, de bereiding en opzuivering van het peptide zijn uitgebreid beschreven in de Investigator's Brochure.

5.5 Risico's en voordelen proefpersonen

AMP60.4Ac is in voorafgaand *in vitro* onderzoek in staat gebleken om LPS te neutraliseren en om de door LPS en LTA geïnduceerde cytokine productie in volbloed te remmen (zie Investigator's Brochure). Hieruit blijkt dat *in vitro* de werking van het synthetische peptide vergelijkbaar is met die van het humane LL-37. Bovendien is gebleken dat het synthetische peptide een lagere T-cell proliferatie en minder cytokine productie *in vitro* veroorzaakt dan LL-37. Het lijkt er dus op dat er geen immuunrespons van het peptide is te verwachten. Verder hebben we aangetoond dat het synthetische peptide een lagere activatie van het MAPkinase ERK1/2 (een maat van celactivatie) induceert in bronchiale epitheelcellen. In een chemotaxie bepaling is gebleken dat peptide AMP60.4Ac geen chemotaxis van neutrofiele granulocyten induceert, terwijl dit wel werd aangetoond voor twee andere door ons ontwikkelde synthetische peptiden en voor LL-37. Op basis van bovengenoemd onderzoek worden dus geen negatieve reacties van het onderzoeksproduct verwacht. Dit in tegenstelling tot oordruppels met antibiotica waarvan aangetoond is dat deze in sommige gevallen ototoxisch zouden kunnen zijn.[24,25,26,27] In een onderzoek naar mogelijke toxiciteit van AMP60.4Ac bij ratten zijn geen nadelige effecten gevonden van het peptide. De maximale getolereerde dosis getest in een dosis escalatie studie met ratten was hoger dan 8 mg/kg/dag. De hoogste dosis getest was 8 mg/kg/dag, hierbij zijn geen nadelige effecten gevonden.

Deze nieuwe behandeling kan een verbeterde genezing van chronische OME bewerkstelligen t.o.v. huidige behandelingsmethoden. Bovendien hebben vergelijkbare peptiden geen ontstaan van antibiotica resistentie laten zien, zijn veilig gebleken bij mucosale toediening in zalf (www.genaera.com) en worden momenteel

getest in diverse klinische studies.[28,14] In de fase I studie, waarbij 16 patiënten met therapieresistente chronische otitis media werden onderzocht, werden geen bijwerkingen aangetoond.

5.6 Inbreng participerende instelling

Het LUMC heeft samen met OctoPlus een Letter of Intent opgesteld waarin de samenwerking aangaande dit project is vastgelegd. OctoPlus heeft een ruime expertise op het gebied van drug development en -delivery, en kunnen daarom een belangrijke bijdrage leveren aan dit project.

6. Hypothese / Vraagstelling

Hypothese:
Deze nieuwe behandeling zal een verbeterde genezing van chronische middenoorontsteking met trommelvliesperforatie bij volwassenen bewerkstelligen.

Vraagstelling:
De vraagstelling van de dose-finding studie was om te onderzoeken of applicatie van AMP60.4Ac rechtstreeks in het middenoor van volwassenen met chronische middenoorproblemen met een trommelvliesperforatie:
1.Geen toxische bijwerkingen heeft, en
2.Welke concentratie peptide zal zorgen voor een verbetering van het middenoorslijmvlies richting een droog, plat gezond middenoorslijmvlies.

Bij de dose-finding studie zijn geen ernstige nadelige bijwerkingen gemeten en de optimale dosis is bepaald op 0.5 mg/ml. De vraagstelling die in het tweede deel van de studie onderzocht zal worden is of de nieuwe behandeling een verbetering van het middenoorslijmvlies ten opzichte van de placebo behandeling te zien geeft. In dit gedeelte zal nog steeds de veiligheid van de nieuwe behandeling gecontroleerd worden.

7. Doelstelling

De primaire doelstelling van deze studie is om te onderzoeken of AMP60.4Ac veilig toegediend kan worden op ontstoken slijmvlies van het middenoor bij volwassenen, zonder dat er nadelige bijwerkingen optreden.

De secundaire doelstelling is het onderzoeken of de nieuwe behandeling een verbetering van het middenoorslijmvlies teweegbrengt ten opzichte van een placebo behandeling.

8. Onderzoekspopulatie

De onderzoekspopulatie betreft een groep volwassen patiënten met langdurige middenoorproblemen (chronische otitis media en een trommelvliesperforatie). De beoogde groep patiënten is eerder zonder resultaat behandeld geweest met antibiotica. Tijdens de studie zullen patiënten geen antibiotica gebruiken. Alle medicatie die in het afgelopen halfjaar gebruikt is zal worden geregistreerd in de Case Report formulieren.

Recrutering:
De recrutering voor de dose-finding studie heeft plaatsgevonden uit de bestaande patiëntenpopulatie van de afdeling KNO van het LUMC door de hoofdonderzoeker (behandelend arts) van deze studie. Voor het gerandomiseerde deel van de studie zullen naast patiënten uit het LUMC tevens patiënten gerecruteerd worden in het Academisch Medisch Centrum te Amsterdam en het Erasmus Universitair Medisch Centrum van Rotterdam. De onderzoekers zullen de patiënten informeren over de

voor- en nadelen van deelname aan de studie en zorgdragen dat hun privacy gewaarborgd blijft. Voor deelname aan de studie zullen de deelnemers een informed consent (zie bijlage) ondertekenen.

Inclusie criteria:
-- volwassenen ≥ 18 jaar
-- wilsbekwaam, geen psychiatrische voorgeschiedenis
-- chronische proliferatieve slijmvliesverandering met duidelijke trommelvliesperforatie > 3 maanden
-- antibiotica therapie resistent (duur therapie >3 maanden)

Exclusie criteria:
-- cholesteatoom
-- antibiotica of corticosteroiden gebruik (locaal en/of systemisch) tot 4 weken voorafgaande aan de studie en tijdens de studie
-- gebruik van prednison of andere immunosuppressiva
-- syndroom van Down
-- afweerstoornissen, inclusief auto-immuunstoornissen
-- duizeligheidklachten, ernstige hoofdpijn of facialisafwijkingen
-- zwangerschap
-- eerder inclusie in dose finding studie

Controlegroep:
Om het effect van het vehiculum van AMP60.4Ac te onderzoeken wordt er voor de gerandomiseerde studie een controle groep ingesteld, die behandeld worden met de oordruppels zonder peptide (placebo).

Aantal proefpersonen:
Voor het dubbelblind gerandomiseerde deel van de studie is het aantal proefpersonen bepaald op 52 (zie 14.3). Deze worden onderverdeeld in 26 die placebo oordruppels krijgen en 26 die behandeld worden met AMP60.4Ac oordruppels.

9. Onderzoeksopzet

9.1 Ontwerp

De studie is een gerandomiseerd dubbelblind placebo-gecontroleerd onderzoek naar de veiligheid en de werkzaamheid van AMP60.4Ac voor de behandeling van chronische middenoorontsteking bij volwassenen. Oordruppels met het experimenteel medicament AMP60.4Ac zal versus het vehiculum voor AMP60.4Ac (zie voor omschrijving paragraaf 9.2) onderzocht worden. Momenteel is er geen goede behandelingsmethode voor chronische slijmvliesafwijkingen van het middenoor bij volwassenen. Daarom wordt de studiemedicatie vergeleken met een placebo. Er is gekozen voor een placebo-gecontroleerde studie omdat op deze manier de werking van het vehiculum ook kan worden onderzocht. Omdat het niet ethisch is het te onderzoeken medicament bij gezonde vrijwilligers, die geen trommelvliesperforatie hebben, in het middenoor te brengen voor een fase I veiligheidsstudie, is gekozen voor een gecombineerde fase I/II studie waarbij zowel de veiligheid als de werkzaamheid van AMP60.4Ac bij patiënten onderzocht wordt.

Bij aanvang van de studie zullen de volgende patiëntengegevens verzameld worden:
-- leeftijd
-- chronische slijmvliesafwijkingen met trommelvliesperforatie; links / rechts
-- aspect trommelvlies en middenoorslijmvlies
-- voorafgaande medicatie
-- co-medicatie

-- bovenste luchtweginfecties
-- gehoortest (toon/spraak-audiogram), bij aanvang en bij laatste bezoek tevens hoge tonen audiometrie
-- CT-scan van middenoor en mastoid
-- bacteriekweek van middenoor en keel
-- bloedafname voor bepaling van specifieke peptide antilichamen en algemeen onderzoek (Hb, Ht, BSE, en autom. Diff.)

Voor de gerandomiseerde dubbelblind gecontroleerde studie worden 52 patiënten geïncludeerd en vervolgens in 2 groepen verdeeld. Hiervan krijgt de eerste groep AMP60.4Ac opgelost in oordruppels, en de tweede groep krijgt als placebo oordruppels zonder AMP60.4Ac.

Randomizatie:
De deelnemers met een éénzijdige proliferatieve slijmvliesverandering met trommelvliesperforatie zullen at random door middel van loting ingedeeld worden in de behandeling- of de placebogroep. Hiertoe zullen de flesjes oordruppels vooraf worden genummerd door een onafhankelijk persoon, die de sleutel bewaart tot aan het einde van het onderzoek volgens de standaard procedures. Bij patiënten met een tweezijdige slijmvliesafwijkingen wordt slechts een van beide oren behandeld.

Blindering:
Er zal een dubbele blindering plaatsvinden, zodat de patiënten zelf en de onderzoekers niet op de hoogte zijn van de ontvangen behandeling.

Stratificatie:
De randomisatie vindt plaats gestratificeerd naar deelnemend centrum middels een gebalanceerd blokken design. De allocatie wordt door de verantwoordelijk statisticus voor de gehele studie van te voren gegenereerd en in electronische vorm aan de leverancier van de de flesjes geleverd.
Ter voorkoming van onbalans dient – gezien de groepsgrootte per centrum – elk centrum blokken geheel vol te maken, zelfs als dat een overschreiding van het totale aantal patienten in de studie ten gevolge zou hebben. Gemiddeld zal per centrum een 18 tal personen worden geincludeerd, zodat een variable blocked design met blokgroottes van 2 en/of 4 tot de mogelijkheden behoren. Als het verwachtte aantal patienten in een centrum kleiner dan 9 is, wordt met een fixed blocksize van 2 gewerkt

9.2 Onderzoeksbehandeling

Product: AMP60.4Ac is een synthetisch peptide afgeleid van het humane cathelicidin LL-37. LL-37 is een cationisch eiwit aanwezig in neutrofiele granulocyten en epitheelcellen, dat antibacteriële eigenschappen heeft. AMP60.4Ac is geproduceerd onder GMP-condities in het door de Farmaceutische Inspectie van IGZ gevisiteerde peptidelaboratorium van de Interdivisionele GMP-faciliteit van het LUMC. Het peptide wordt opgelost in oordruppels (0.02% benzalkoniumchloride, 0.1% dinatriumedetaat, 10% PEG 10.000 in isotone buffer [154 mM NaCl en 20 mM natriumfosfaat] pH=6) voor optimale afgifte door de slijmlaag heen aan de mucosa. AMP60.4Ac bestaat uit 24 aminozuren met de volgende sequentie: IGKEFKRIVERIKRFLRELVRPLR (geacetyleerd en geamideerd). Mede op basis van de resultaten beschreven in de investigator's brochure hebben we besloten om de klinische studie met peptide 60.4Ac uit te voeren. Alle aminozuren zijn hetzelfde als van peptide 60.4 maar de uiteinden zijn iets veranderd (geacetyleerd en geamineerd) met het oog op extra stabiliteit tegen enzymatische afbraak.

Dosering: enkele druppels (±100 µl) AMP60.4Ac 2x daags gedurende 2 weken via oordruppels op het trommelvlies. Patiënten dienen de oordruppels na instructie zelf

thuis toe. Door de trommelvliesperforatie ontstaat een rechtstreekse applicatie op het middenoorslijmvlies van AMP60.4Ac. In de dose-finding studie werd allereerst een optimale dosis voor de behandeling gezocht. Hiertoe werden de peptide concentraties 0.25 / 0.5 / 1.0 en 2.0 mg/ml onderzocht.

Motivering van de dosering: uit het vooronderzoek (zie Investigator's Brochure) naar de werkzaamheid is gebleken dat 1 ng/ml LPS voor 50% geneutraliseerd wordt door 2-4 μg/ml peptide en 100% neutralisatie word bereikt bij een concentratie van 5-8 μg/ml; veiligheidshalve gaan we uit van ≈ 10 μg/ml (ofwel: 10.000X overmaat). In het oor is een concentratie van gemiddeld 1.5, 18 of 96 ng/ml LPS aanwezig afhankelijk van het type middenooreffusie.[11] Als we uitgaan van een concentratie van 100 ng/ml dan zouden we met een concentratie van 1 mg/ml Peptide Solution het aanwezige LPS in het oor kunnen neutraliseren. Uit de dose-finding studie is de optimale concentratie bepaald op 0.5 mg/ml.
In de dose-finding studie is een linear mixed model gefit aan de onderzoeksgegevens waarbij de 4-punts-schaal voor het kwantificeren van het otoscopisch beeld is gebruikt. Een kwadratisch model voor de relatie tussen concentratie en het otoscopisch beeld werd als uitgangspunt gekozen en getoetst werd of de kwadratische term significant was. Dit was niet het geval zodat geconcludeerd moest worden dat binnen het gegeven meetbereik voor de concentraties (0.25, 0.5, 1 en 2) er geen optimum geschat kon worden. De lineaire term was significant, waarbij een klinisch relevant verschil ten gunste van de beide laagste doses gevonden werd. Het verschil tussen de beide laagste doseringen was:

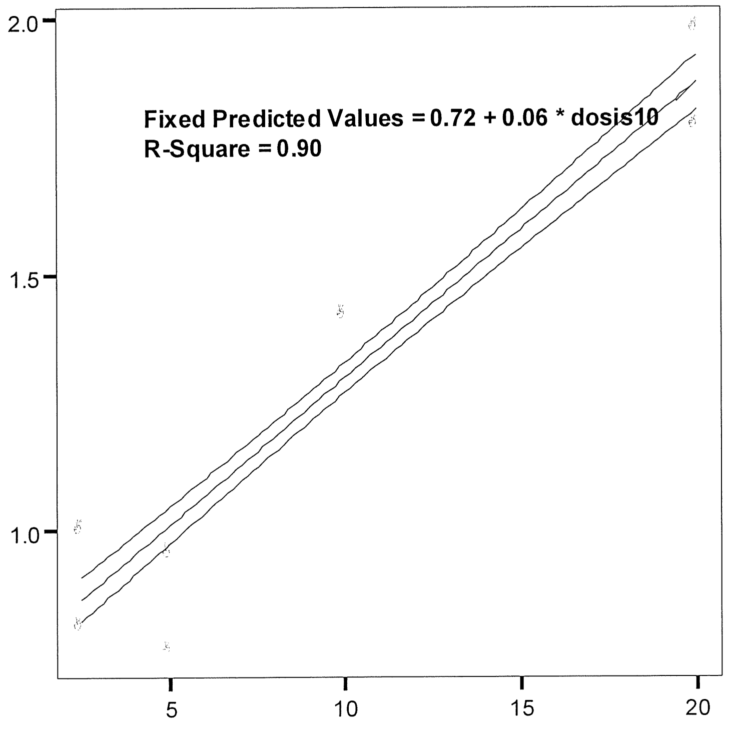

Horizontaal staat de gegeven dosis*10. Het verschil tussen de doses 0.5 en 0.25 is (geobserveerd zowel als voorspeld) minimaal zodat op klinisch-inhoudelijke gronden voor de dosis van 0.5 gekozen is.

Behandelingsperiode: Voor alle deelnemers aan zowel de dose-finding, als ook aan de gerandomiseerde studie geldt dat op week 1, 2, 4, 8 en 12 inspectie van het trommelvlies en het middenoor zal plaatsvinden De toestand van het middenoor en het mastoid wordt tevens bekeken m.b.v. een CT-scan aan het begin en het eind van de studie.

Doseringsschema: 2x daags enkele oordruppels (ca. 100 µl), gedurende 2 weken

Doseringsvorm: Vloeibaar

Verpakking, etikettering: druppelflesje, 2x daags twee druppels, gedurende 2 weken.

Toegestane / niet toegestane medicatie: geen antibiotica toegestaan, overige medicatie in overleg met behandelend arts

Duur van het onderzoek: 12 weken na eerste toediening

Instructies: Op het etiket van de oordruppels komen duidelijke instructies en ook krijgt de patiënt schriftelijke en mondelinge instructies van de behandelend arts.

Verantwoording drugaccountability: Hoeveelheid AMP60.4Ac dat zal worden gebruikt in de studie wordt gedocumenteerd en gecontroleerd door de onderzoekers en de relevante secties binnen de afdeling klinische Farmacie en Toxicologie.

9.3 Schematisch diagram

Stap 1: Bezoek van de patiënt aan de onderzoeker
Procedure: Beoordeling in- en exclusie criteria
⇓
Inclusie in de studie, randomizatie (niet bij de dose-finding)
⇓
Invullen screening formulier van het case report form (CRF)
Inspectie trommelvlies/middenoorslijmvlies
Maken toon-audiogram+hoge tonen audiometrie
Maken CT-scan mastoid
Afname van middenoor- en keelkweek
Afname bloed (1 stolbuis voor spec.antilich.bep. en 1 buis voor Hb,Ht, BSE, en diff.)
⇓
Stap 2: Behandeling
Procedure: Start medicatie AMP60.4Ac / placebo (week 0)
⇓
Stap 3: Controle bezoeken bij de onderzoeker in week 1, 2, 4, 8 en 12
Procedure: invullen controle bezoek formulier van het CRF
Inspectie trommelvlies/middenoorslijmvlies
Maken toon-audiogram
⇓
Stap 4: Einde van de studie in week 12
Procedure: Invullen completion pagina van het CRF
Inspectie trommelvlies/middenoorslijmvlies
Maken toon-audiogram+hoge tonen audiometrie
Maken CT-scan mastoid
Afname van middenoor- en keelkweek
Afname bloed (wk 8, 1 stolbuis voor spec.antilich.bep. en 1 buis voor Hb,Ht, BSE, en diff.)

9.4 Behandelschema

	Week 0<br>Visite 1	Week 1<br>Visite 2	Week 2<br>Visite 3	Week 4<br>Visite 4	Week 8<br>Visite 5	Week 12<br>Visite 6
Beoordeling In- Exclusie criteria	X					
Toestemmingsverklaring	X					
Demografische data	X					
Medische geschiedenis	X					
Gebruik co-medicatie	X	X	X	X	X	X
Lichamelijk onderzoek	X					
CT-scan	X					X
Bloedafname	X				X	
Afnemen Oorkweek	X					X
Afnemen Keelkweek	X					X
Beoordelen trommelvliesaspect	X	X	X	X	X	X
Stadium middenoor-slijmvlies	X	X	X	X	X	X
Digitale foto (optioneel)	X	X	X	X	X	X
Audiogram	X	X	X	X	X	X
Hoge tonen audiometrie	X					X

9.5 Duur en omvang van het onderzoek

Tijdschema hoofdstudie:

	2005<br>Mei	Juni	Juli	Aug	2006<br>Sept	Okt	Nov-Dec	Jan-Mrt	2007<br>April-Juni	Juli-Aug	Sep
Afronden dose-finding	XX	XX	XX	XX							
Bepalen doses				X							
Start hoofdstudie					X						
Recrutering						XXXX	XXXX	XXXX	XXXX		
Follow-up							XXXX	XXXX	XXXX	XXXX	
Invoeren gegevens							XX	XXXX	XXXX	XXXX	
Disclosure sleutel											X
Resultaten uitwerken											XXXX

Logistieke procedures:
De arts-onderzoeker/behandelaar zal de patiënten recruteren en de afspraken maken voor de geplande onderzoeken. Na eventuele insluiting zullen de proefpersonen op de geplande visites onderzocht worden door de arts-onderzoeker. Het toon-audiogram

zal door een audiometrie-assistent worden afgenomen. Voor de CT-scan zal een afspraak worden gemaakt op de betreffende afdeling.
De monitor zal toezien op de juiste invulling van de case report formulieren. De gegevens zullen worden ingevoerd in een database door de afdeling datamanagement en statistiek. Na disclosure van de sleutel zullen de resultaten door de onderzoekers worden uitgewerkt.
Een monitor zal tussentijds en aan het eind van het onderzoek een controle verrichten op het uitvoeren van de procedures door de onderzoekers.

10. Eindpunten

10.1 Primaire en secundaire eindpunten:

Het primaire eindpunt van de studie zal zijn dat er geen toxische bijwerkingen als gevolg van de behandeling worden gevonden.

Het secundaire eindpunt is het herstel van het middenoorslijmvlies en het trommelvlies.

10.2 Meetmethoden

Eventuele toxische reacties worden geëvalueerd door middel van:
-- inspectie van het oor
-- controle van het gehoor mbv een toon-audiogram

De parameters waarmee de werkzaamheid van de behandeling zal worden geëvalueerd zijn:
-- aspect trommelvlies en middenoorslijmvlies d.m.v. oorinspectie
-- CT-scan van middenoor en mastoid

11. Methode van meten

Het audiogram zal poliklinisch worden gemaakt voorafgaand aan de behandeling en op week 1, 2, 4, 8 en 12 na behandeling door de onderzoeker. De oorinspectie dient om de werkzaamheid in kaart te kunnen brengen. Het otoscopisch beeld wordt beschreven en gekwantificeerd in vier stadia:
0=plat, droog middenoorslijmvlies
1=plat, nat middenoorslijmvlies
2=verdikt polypeus, droog of nat middenoorslijmvlies
3=verdikt polypeus, middenoorslijmvlies met taai slijm

Het toon-audiogram dient als veiligheidsparameter om eventuele gehoorbeschadiging door de studiemedicatie te kunnen meten. De CT-scan zal alleen bij aanvang en op week 12 worden uitgevoerd om tekenen van ontstekingsreacties in het mastoid te kunnen detecteren. Analyse van de CT-scan wordt uitgevoerd door de arts-onderzoeker/behandelaar. Resultaten worden vastgelegd in het patiëntendossier en in het CRF.

Aanvullend onderzoek:
Indien mogelijk wordt er bij aanvang van de studie middenooreffusie afgenomen voor een bacteriekweek. Dit om inzicht te verkrijgen in de bacteriologie van otitis media chronica bij volwassenen. Vooraf en na afloop van de studie zal er naast de middenoorkweek ook een keelkweek uitgevoerd worden om een eventuele verschuiving in de populatie micro-organismen vast te stellen. Tenslotte zal er vooraf en op week 8 in de studie bloed worden afgenomen voor een algemene bloedanalyse

en een bepaling van eventuele antilichamen die zich tegen het peptide gevormd zouden kunnen hebben.

12. Lichaamsmaterialen

Alle lichaamsmaterialen zullen worden gecodeerd. De uitslag van de bacteriekweek en het bloedonderzoek kan dan na afloop van de studie gekoppeld worden aan de bijbehorende patiënt. Het materiaal is zo dus indirect herleidbaar tot de betreffende proefpersoon. De sleutel van de code is in handen van de behandelaar. Alleen deze kan de uitslag van de bepaling na afloop van de studie aan de betreffende persoon koppelen. Na afloop van het onderzoek is het materiaal 'op' of wordt het resterende materiaal vernietigd.

Over de afname van het materiaal wordt de proefpersoon zowel mondeling als schriftelijk geïnformeerd. Hierbij wordt ook vermeld op welke wijze het materiaal wordt verwerkt. Met het tekenen van de toestemmingsverklaring geeft de proefpersoon toestemming voor de afname en verwerking van het materiaal. Dit gebeurt in het kader van het onderzoek.

13. Methode van uitvoering / Laboratoriumwerkwijze

Voor nader wetenschappelijk onderzoek naar de bacteriologie van chronische otitis media met effusie bij volwassenen, zal een bacteriekweek van middenooreffusie worden uitgevoerd. Tevens zal vooraf en na afloop van de studie een middenoor- en keelkweek uitgevoerd worden voor de bepaling van eventuele verschuiving in micro-organisme populatie. Deze bacteriekweek zal volgens standaard protocol in het desbetreffende ziekenhuis bij de afdeling bacteriologie worden gedaan. Tenslotte zal vooraf en in week 8 van de studie een bloedmonster worden afgenomen voor analyse. Hierbij zal de bepaling van het algemeen bloedbeeld (Hb, Ht, differentiatie analyse) in het desbetreffende ziekenhuis worden uitgevoerd en de specifieke peptide-antilichaam bepaling zal in het LUMC gedaan worden. Het hiervoor benodigde serum zal in het desbetreffende ziekenhuis worden bewaard bij −20°C en door de monitor worden meegenomen naar het LUMC voor analyse.

14. Statistische analyses

14.1 Statistische methoden

Het otoscopisch beeld wordt beschreven en gekwantificeerd in vier stadia:
0=plat, droog middenoorslijmvlies
1=plat, nat middenoorslijmvlies
2=verdikt polypeus, droog of nat middenoorslijmvlies
3=verdikt polypeus, middenoorslijmvlies met taai slijm

Geanalyseerd zal worden hoeveel patiënten met de therapeutische behandeling een verbetering van het middenoorslijmvlies hebben na afloop van de studie vergeleken met de controle groep. Enerzijds zullen hiertoe de gemiddelde scores worden vergeleken, zowel ten tijde van de laatste meting (week 12) als het verloop in de tijd (repeated measures analysis of variance met de 0-meting als covariaat ter verhoging van de power). Daarnaast zal ook het percentage proefpersonen met een duidelijke verbetering bij week 12 worden vergeleken als binaire uitkomst maat, waarbij een duidelijke verbetering wordt gedefinieerd als ten minste 2 stappen richting normaal

plat, droog middenoorslijmvlies (derhalve 3->1, 3->0 of 2->0). Deze laatste analyse ligt ten grondslag aan de sample size berekening.

14.2 Statistische analyse

De statistische analyse zal uitgevoerd worden door de biostatisticus R. Brand van de afdeling Medische Statistiek en Advanced Data Management. De vergelijking van de gemiddelde scores op week 12 alsmede de vergelijking van het verloop van de scores gelden als analyses van de primaire uitkomst maat. Datzelfde geldt ook voor de vergelijking van het percentage personen met een "duidelijke verbetering". De eerste analyses zijn parametrische analyses, de laatste een logistische regressie-analyse waarbij de randomisatie-groep als groepsindeling, het al dan niet hebben van een "duidelijke verbetering" als uitkomst en andere factoren voor zover bepaald voorafgaande aan het moment van randomisatie als covariaten meegenomen (kunnen) worden, zulks met het doel de power van de groeps vergelijking te verhogen.

14.3 Aantal proefpersonen

Het aantal proefpersonen voor deze studie is berekend met het software pakket East (versie 3.1.0) waarin de mogelijkheid van interim-analyses met ene alpha en beta spending functie wordt geboden. De East software wordt ook gebruikt door de FDA bij de evaluatie van clinical trial resultaten en is gebaseerd op internationaal erkende biostatistische methodes.
Er is gekozen voor een sample size bereking op basis van de vergelijking van twee proporties waarbij er is uitgegaan dat een kans van slagen van de controle behandeling (placebo) van 20% en een slagingskans van de te onderzoeken behandeling van 60% te onderscheiden moeten zijn met een power van 90% bij een tweezijdige toetsing met significantie niveau alpha van 5%.

Er is gekozen voor 1 interim analyse bij een informatie-fractie van 50% (d.w.z. nadat 50% van het benodigde aantal proefpersonen de meting van week 12 heeft bereikt). Bij een zeer snelle accrual zal de interim-analyse niet tot een reductie van het benodigde aantal proefpersonen kunnen leiden (er zijn nog 12 weken accrual te gaan nadat 50% van het aantal proefpersonen is geincludeerd). Mocht de accrual langzamer gaan dan verwacht, dan ontstaat de volgende situatie:

De trial krijgt zowel power voor efficacy als futility; we gebruiken een benadering van de O'Brien-Fleming boundaries in de context van alpha en beta spending. Het maximum aantal benodigde proefpersonen is 52 (2 maal 26); onder de nul-hypothese is het verwachtte aantal 49 (waarbij de uitkomst "futility" kan worden vastgesteld; onder H1 is dit 46 (waarbij de uitkomst "efficacy" wordt vastgesteld); en halverwege H0 en H1 (dwz een verschil van 0.2 ipv 0.4) mogen we totaal 50 inclusies verwachten.

De volgende tabel geeft de kansen weer om de studie bij interim analyse reeds te kunnen beeindigen:

Information Fraction	Cumulative Accrual	Alpha Spent	Beta Spent	Stopping Probabilities		
				Under H0	Under H1	Under H1/2
0.500	26.579	0.003	0.011	0.16	0.27	0.12
1.000	53.157	0.050	0.100	0.84	0.73	0.88

Hieruit blijkt dat bij analyse na 26 evalueerbare personen, onder aanname dat beide groepen hetzelfde resultaat vertonen(H0) er 16% kans is de studie reeds wegens "geen verschil" te beeindigen. Zo is er 27% kans om de studie wegens een significant verschil te kunnen afsluiten na 26 personen als in werkelijkheid het verschil inderdaada 60 versus 20% is.

Door deze interim analyse wordt derhalve een substantiele kans op kostenbesparing ingebouwd in het design.

Indien bij interim analyse de trial gestopt wordt, gaat de follow-up van de reeds geincludeerde personen gewoon door. Echter, de 12-weeks data van de personen die niet in de interim analyse betrokken waren, kunnen NIET meer voor een eind-analyse gebruikt worden omdat de interim-analyse reeds als eindanalyse geldt en een herhaling van deze analyse ondanks het feit dat de van te voren vastgestelde stopping-boundaries zijn overschreven, een niet-acceptabele verandering van de voor-ingestelde alpha en beta geven.
Dit laat onverlet dat men wel de gegevens van de complete voor safety doeleinden mag en moet gebruiken; echter, de conclusie betreffende het efficacy aspect is reeds getrokken.

15. Uitkomsten/bevindingen en hun implicaties

De uitkomsten van het onderzoek zullen worden uitgedrukt in score van het middenoorslijmvlies, aantallen genezen patiënten, en safety. De resultaten zullen worden gepubliceerd in internationale wetenschappelijke tijdschriften.
Na afloop van de studie zullen de deelnemende proefpersonen worden geïnformeerd over de individuele en de algemene resultaten.

16. Ethische overwegingen

Deze studie zal naar verwachting leiden tot nieuwe inzichten op het gebied van de behandeling en de bacteriologie van chronische otitis media bij volwassenen. Deze nieuwe inzichten kunnen tevens belangrijk zijn voor de verdere ontwikkeling van behandelingen voor andere (bovenste) luchtweg infecties en otitis media met effusie bij kinderen. Daarom achten wij het belang van het onderzoek evenredig aan de bezwaren en risico's. De verwachtte resultaten kunnen niet verkregen worden zonder deze patiëntenstudie waarvan de leiding en uitvoering in handen zal zijn van deskundigen. Bovendien zal de persoonlijke levenssfeer beschermd worden en zal het belang van de deelnemers prevaleren boven het belang van de wetenschap of de samenleving.

17. Controle vordering van het onderzoek

17.1 Beoordeling van de veiligheid

De belangrijkste veiligheidsparameter is het toon-audiogram. Hiermee kan een eventueel opgetreden perceptief gehoorsverlies aangetoond worden. De test zal voorafgaand aan de behandeling en op week 1, 2, 4, 8 en 12 na de behandeling poliklinisch worden afgenomen door een audiometrie-assistent. Resultaten worden vastgelegd in het audiogram dat wordt opgenomen in het patiëntendossier en in het CRF. Verder zal door middel van bacteriekweek van middenoor en keel en bloedanalyse de veiligheid van het peptide extra gecontroleerd worden.

17.2 Beëindiging van het onderzoek en (serious) adverse events (SAEs)

Een ernstig ongewenst voorval (SAE) of ernstige nadelige bijwerkingen (SADR) worden gedefinieerd als elk ongewenst medisch voorval dat bij ongeacht welke dosis: de dood tot gevolg heeft, levensbedreigend is, ziekenhuisopname of verlenging van opname noodzakelijk maakt, tot blijvende of ernstige invaliditeit/arbeidsongeschiktheid leidt of een aangeboren afwijking/geboorteafwijking is.

Stopzetten van het onderzoek:
-- als er onverwachte SADRs zijn bij meer dan 6 patiënten
-- als er SAEs optreden bij meer dan 6 patiënten
-- in overleg met de CME

Criteria voor beëindiging onderzoek individuele patiënten:
-- als er een SAE of een SADR optreedt waarbij de patiënt opgenomen moet worden in het ziekenhuis.
-- als patiënt plotseling doof wordt, of er een toenemend perceptief gehoorsverlies is.
-- bij duizeligheid, facialis afwijkingen of ernstige hoofdpijn.
-- non-compliance van de patient

Rapportage van (serious) adverse events en ziekten tijdens het onderzoek zal plaatsvinden in het patiëntendossier en in het CRF. De duur en nazorg bij (serious) adverse events is in handen van de behandelend arts. De betreffende proefpersoon zal de studie beëindigen. SAE's zullen onmiddellijk gemeld worden aan de proefpersoon, de studie-coordinator en aan de CME en voorzien van commentaar van de onderzoeker.

Procedure uit het onderzoek halen van patiënten:
-- invullen de completion pagina van het CRF, indien mogelijk laatste gegevens verzamelen
-- nazorg verlenen indien noodzakelijk
-- vervanging van de proefpersoon in het onderzoek

17.3 Monitoring Committee

Vanuit de participerende instelling zal een studiemonitor worden aangewezen, die zal controleren of
-- de formulieren goed worden ingevuld
-- er goed wordt gehandeld in geval van SAEs

17.4 Interim-analyses

Op de helft van de hoofdstudie zal er een interim-analyse door de statisticusplaatsvinden. Wanneer bij interim-analyse blijkt dat er bij meer dan 4 deelnemers van de onderzoeksgroep:
-- een serious adverse event is opgetreden waarbij de patiënt opgenomen moest worden
-- plotseling doofheid optreedt, of er een toenemend perspectief gehoorsverlies is
-- duizeligheid, facialis afwijkingen of ernstige hoofdpijn ontstaan
zal het onderzoek beëindigd worden.

Zoals onder 14.3 vermeld, vindt er na 26 evalueerbare proefpersonen een formele interimanalyse ten aanzien van de primaire uitkomstmaat plaats. De trial zal dan gestopt worden indien uit deze analyse conform specificaties de nul-hypothese van gelijkheid van behandeling bij de dan geldende kritieke grens verworpen kan worden (efficay) dan wel de alternatieve hypothese van een verschil tussen de behandeling verworpen kan worden (futility).
Het volgende schema geeft deze beslisgrenzen weer: de eerste figuur geeft de kritieke grenzen op de p-waarde schaal weer; de tweede grafiek vertaalt deze in gevonden verschillen tussen de behandelingen.

p-waarden:

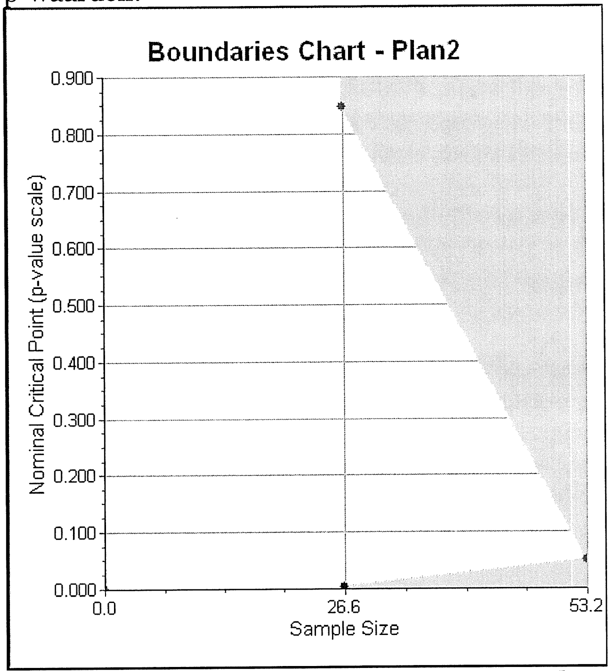

Hieruit blijkt dat indien de p-waarde van de toets welke de proporties duidelijke vooruitgang vergelijkt tussen beide groepen bij interim analyse 0.85 of hoger is, de trial gestaakt wordt wegens futility. Als de p-waarde onder de 0.003 ligt bij interim analyse, wordt geconcludeerd tot verschil van de behandelingen en de trial ook gestopt. Met deze methode blijven de overall alpha en beta op hun oorspronkelijke niveau van 5% resp 90%.

In termen van actuele verschillen zijn deze boundaries als volgt:

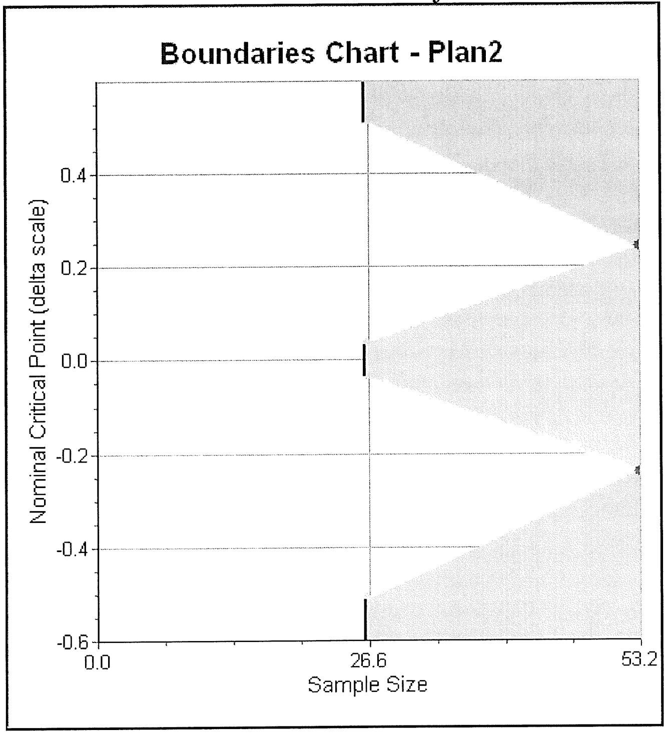

Meer dan 0.51 verschil: stoppen wegens efficacy; minder dan 0.34 verschil stoppen wegens futility (0.51=51% verschil in success percentage)

De error spending bij dit design ziet er als volgt uit:

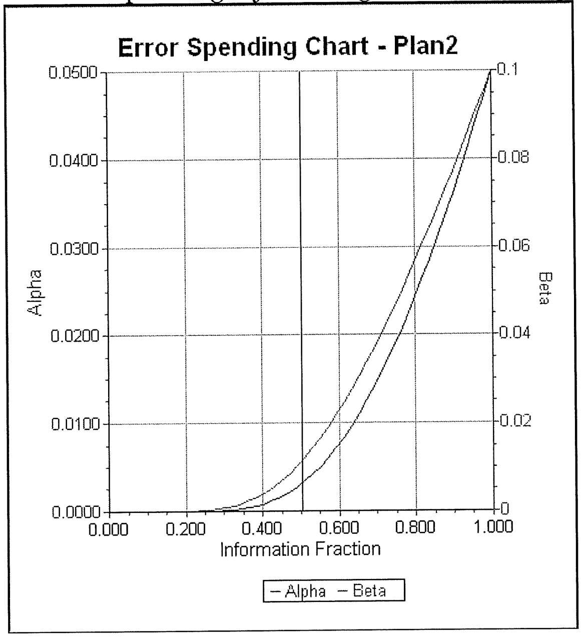

De power van de studie hangt als volgt af van de gemaakte aannames:

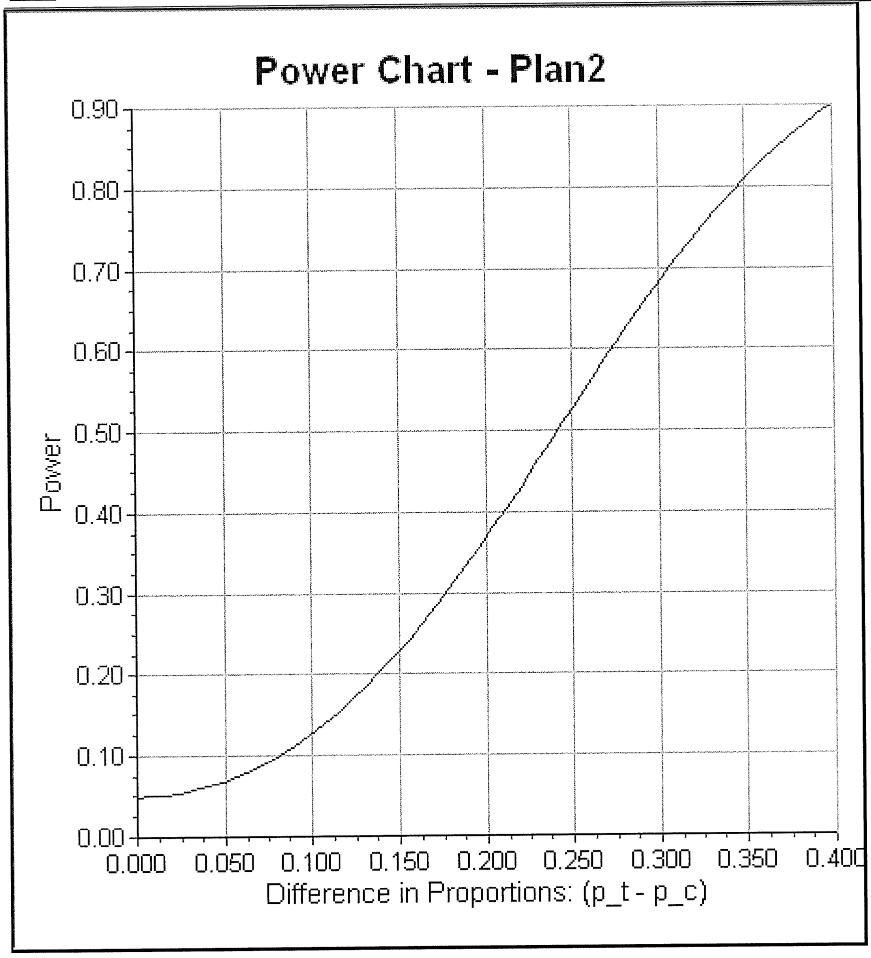

Hieruit blijkt dat als het verschil niet de gepostullerde 40% (60-20) is maar 35%, er nog steeds een power van 80% is om het verschil als significant te detecteren. Bij een verschil in effectiviteit van minder dan 30% is de power niet meer voldoende.

17.5 Studie formulieren en gegevens

De gegevens van de studie zullen worden opgenomen in het patiëntendossier en in de speciale case report formulieren, welke vertrouwelijk zullen worden behandeld. Alleen de onderzoekers, de monitor, de auditor, de ethische commissie, bevoegde autoriteit en de participerende instelling hebben hierin inzage en in de brongegevens. Op de case report formulieren zal alleen een gecodeerd patiënten nummer vermeld worden, zodat er geen directe herleiding is met de proefpersoon.

17.6 Afwijkingen van het protocol

Alle gerandomiseerde proefpersonen zullen worden meegenomen in de intention-to-treat analyse, welke als primaire analyse geldt, zowel qua safety als qua efficacy. Daarna zal een per-protocol analyse naar de efficacy worden uitgevoerd waarbij protocol-violations (ten onrechte gedane inclusies cq ernstige protocol-afwijkingen) buiten beschouwing worden gelaten. Voor de safety analyses worden derhalve nooit patienten uitgesloten.

Rapporteren van afwijkingen van het oorspronkelijke ontwerp:
Afwijkingen zullen d.m.v. een amendement aan het protocol worden toegevoegd. Wijzigingen van het oorspronkelijke protocol worden pas doorgevoerd na goedkeuring van de CME.

18. Publicaties

Na afloop zullen de studieresultaten worden gepubliceerd. Indien relevant zal publicatie van resultaten met de gebruikte studiemedicatie pas plaatsvinden als deze gepatenteerd is. De participerende instelling (OctoPlus BV) heeft daarna voorinzage gedurende een maximum van 6 maanden, maar kan de publicatie niet tegenhouden.

19. Administratieve procedures en verantwoordelijkheden

Codering van proefpersoongegevens, bewaren code lijst
In een code lijst zal worden vastgelegd welke behandeling de deelnemers op volgorde van binnenkomst krijgen.

Verbreken blindering
Na afloop van de studie zal de met behulp van de code lijst gekeken worden welke behandeling de deelnemers hebben ontvangen zodat de resultaten kunnen worden uitgewerkt.

Procedure wijzigen protocol
Een wijziging van het protocol zal na toestemming van alle betrokkenen ter goedkeuring aan de CME worden voorgelegd. Na goedkeuring zal de wijziging als addendum aan het protocol worden toegevoegd.

20. Verzekering

De verzekering van de proefpersonen zal door het betreffende academische centrum waar de patiënten gezien worden geschieden.

21. Referenties

1. Klein, J. O. 2000. Clinical implications of antibiotic resistance for management of acute otitis media. *J Lab Clin Med* 135:220-224.

2. Froom, J., L. Culpepper, M. Jacobs, R. A. DeMelker, L. A. Green, L. van Buchem, P. Grob, and T. Heeren. 1997. Antimicrobials for acute otitis media? A review from the international primary care network. *BMJ* 315:98-102.

3. Klein, J. O. 1994. State-of-the-Art Clinical Article. *Clinical Infectious Diseases* 19:823-833.

4. Faden, H., L. Duffy, and M. Boeve. 1998. Otitis media: back to basics. *Pediatr.Infect.Dis.J.* 17:1105-1113.

5. Nadol, J. B., H. Staecker, and R. E. Gliklich. 2000. Outcomes assessment for chronic otitis media: The chronic ear survey . *Laryngoscope* 110:32-35.

6. Schilder, A. G. M., G. A. Zielhuis, M. P. Haggard, and P. Broek van den. 1995. Long-term effects of otitis media with effusion: otomicroscopic findings. *Am.J.Otology* 16:365-372.

7. Klein, J. O. 1994. Lessons from recent studies on the epidemiology of otitis media. *Pediatr.Infect.Dis.J.* 13:1031-1034.

8. Nell, M. J. and J. J. Grote. 1999. Structural changes in the rat middle ear mucosa due to Endotoxin and Eustachian Tube Obstruction. *Eur.Arch.Otorhinolaryngol.* 256:167-172.

9. DeMaria, Th. F., T. Yamaguchi, and D. J. Lim. 1989. Quantitative cytologic and histologic changes in the middle ear after injection of nontypable *Hemophilus influenzae* endotoxin. *Am.J.Otolaryngol.* 10:261-266.

10. Nell, M. J., B. M. Op 't Hof, H. K. Koerten, and J. J. Grote. 1999. Effect of endotoxin on cultured human middle ear epithelium. *ORL* 61:201-205.

11. Nell, M. J. and J. J. Grote. 1999. Endotoxin and TNF-alpha in middle ear effusions: in relation with upper airway infection. *Laryngoscope* 109:1815-1819.

12. Nell, M. J., B. M. Albers-Op 't Hof, H. K. Koerten, and J. J. Grote. 2000. Inhibition of endotoxin effects on cultured human middle ear epithelium by Bactericidal/Permeability-Increasing protein. *Am.J.Otology* 21:625-630.

13. Nell, M. J., H. K. Koerten, and J. J. Grote. 1999. Bactericidal/permeability-increasing protein prevents mucosal damage in an experimental rat model of chronic otitis media with effusion. *Infection and Immunity* 68:2992-2994.

14. Zasloff, M. 2002. Antimicrobial peptides of multicellular organisms. *Nature* 415:389-395.

15. Bals, R., X. Wang, M. Zasloff, and J. M. Wilson. 1998. The peptide antibiotic LL-37/hCAP-18 is expressed in epithelial of the human lung where it has broad antimicrobial activity at the airway surface. *Proc.NatlAcad.Sci.USA* 95:9541-9546.

16. Agerberth, B., J. Grunewald, E. Castaños-Velez, B. Olsson, H. Jörnvall, H. Wigzell, A. Eklund, and G. H. Gudmundsson. 1999. Antibacterial components in bronchoalveolar

lavage fluid from healthy individuals and sarcoidosis patients. *Am.J.Respir.Crit.Care Med.* 160:283-290.

17. Pütsep, K., G. Carlsson, H. G. Boman, and M. Andersson. 2002. Deficiency of antibacterial peptides in patients with morbus Kostmann: an observation study. *The Lancet* 360:1144-1149.

18. Bals, R. 2000. Epithelial antimicrobial peptides in host defense against infection. *Respir Res* 1:141-150.

19. Yang, D., O. Chertov, and J. J. Oppenheim. 2001. Participation of mammalian defensins and cathelicidins in anti-microbial immunity: receptors and activities of human defensins and cathelicidin (LL-37) . *J.Leukocyte Biology* 69:691-697.

20. Hancock, R. E. W. and G. Diamond. 2000. The role of cationic antimicrobial peptides in innate host defences. *Trends in Microbiology* 8:402-410.

21. Nizet, V., T. Ohtake, X. Lauth, J. Trowbridge, J. Rudisill, R. A. Dorschner, V. Pestonjamasp, J. Piraino, K. Huttner, and R. L. Gallo. 2001. Innate antimicrobial peptide protects the skin from invasive bacterial infection. *Nature* 414:454-457.

22. Frohm, M., B. Agerberth, G. Ahangari, M. Ståhle-Bäckdahl, H. Wigzell, and G. H. Gudmundsson. 1997. The expression of the gene coding for the antibacterial peptide LL-37 is induced in human keratinocytes during inflammatory disorders. *J.Biol.Chemistry* 272:15258-15263.

23. Paulsen, F., T. Pufe, L. Conradi, D. Varoga, M. Tsokos, J. Papendieck, and W. Petersen. 2002. Antimicrobial peptides are expressed and produced in healthy and inflamed human synovial membranes. *J.Pathol.* 198:369-377.

24. Marais, J. and J. A. Rutka. 1998. Ototoxicity and topical eardrops. *Clin.Otolaryngol.* 23:360-367.

25. Lundy, L. B. and M. D. Graham. 1993. Ototoxicity and ototopical medications: a survey of otolaryngologists. *Am.J.Otology* 14:141-146.

26. Russell, N. J., K. E. Fox, and R. E. Brummett. 1979. Ototoxic effects of the interaction between kanamycin and ethacrynic acid. *Acta Otolaryngol.* 88:369-381.

27. Tange, R. A. and E. H. Huizing. 1980. Hearing loss and inner ear changes in a patient suffering from severe gentamicin ototoxicity. *Arch Otorhinolaryngol* 228:113-121.

28. Lehrer, R. I. and T. Ganz. 2002. Cathelicidins: a family of endogenous antimicrobial peptides. *Current Opinion in Hematology* 9:18-22.

1.1 Handtekeningenblad

De hieronder genoemde betrokkenen van het LUMC en de participerende instelling verklaren hierbij accoord te gaan met dit studieprotocol.

Drs. F.A.W. Peek
Hoofdonderzoeker

Dr. J.W. Drijfhout
Immunohematologie en Bloedtransfusie (IHB)

Prof. Dr. P.S. Hiemstra
Longziekten

Drs. G. Slappendel
Apotheker

Titel:

Behandeling van chronische middenoorontsteking bij volwassenen

met behulp van het antimicrobieel peptide AMP60.4Ac

Verrichter: Leids Universitair Medisch Centrum
Albinusdreef 2, 2333 ZA, Leiden

Uitvoerder: Drs. F.A.W. Peek
Afdeling Keel- Neus- en Oorheelkunde
Leids Universitair Medisch Centrum

Statisticus: Dr. R. Brand
Medische Statistiek

Andere betrokkenen: Dr. J.W. Drijfhout
Immunohematologie en Bloedtransfusie (IHB)

Dr. P.S. Hiemstra
Longziekten

Drs. G. Slappendel
Klinische Farmacie en Toxicologie

Erasmus Universitair Medisch Centrum, Rotterdam

Participerende Instelling: OctoPlus Technologies BV.
Zernikedreef 12
2333 CL Leiden

Financiering: LUMC en OctoPlus Technologies BV.

Contactpersonen: Drs. F.A.W. Peek
Tel. 071-526 2434

Onafhankelijk arts: Dr. J.P.R. Merkesteyn (stafarts LUMC)
Tel. 071-526 2371

1. Handtekeningenblad

De hieronder genoemde betrokkenen van het LUMC en de participerende instelling verklaren hierbij accoord te gaan met dit studieprotocol.

Drs. F.A.W. Peek
Hoofdonderzoeker

Dr. J.W. Drijfhout
Immunohematologie en Bloedtransfusie (IHB)

Prof. Dr. P.S. Hiemstra
Longziekten

Drs. G. Slappendel
Apotheker

2. Inhoudsopgave

1. Handtekeningenblad 2

2. Inhoudsopgave 3

3. Lijst met afkortingen 5

4. Samenvatting 5

5. Inleiding 6

5.1 Achtergrond 6
5.2 Huidige situatie 6
5.3 Pre-klinisch onderzoek 6
5.4 Onderzoeksproduct 7
5.5 Risico's en voordelen proefpersonen 7
5.6 Inbreng participerende instelling 8

6. Hypothese / Vraagstelling 8

7. Doelstelling 8

8. Onderzoekspopulatie 8

9. Onderzoeksopzet 9

9.1 Ontwerp 9
9.2 Onderzoeksbehandeling 11
9.3 Schematisch diagram 13
9.4 Behandelschema 14
9.5 Duur en omvang van het onderzoek 14

10. Eindpunten 15

10.1 Primaire en secundaire eindpunten: 15
10.2 Meetmethoden 15

11. Methode van meten 15

12. Lichaamsmaterialen 16

13. Methode van uitvoering / Laboratoriumwerkwijze 16

14. Statistische analyses 16

14.1 Statistische methoden 16
14.2 Statistische analyse 17

14.3 Aantal proefpersonen *17*

15. Uitkomsten/bevindingen en hun implicaties **18**

16. Ethische overwegingen **18**

17. Controle vordering van het onderzoek **18**

17.1 Beoordeling van de veiligheid *18*

17.2 Beëindiging van het onderzoek en (serious) adverse events (SAEs) *18*

17.3 Monitoring Committee *19*

17.4 Interim-analyses *19*

17.5 Studie formulieren en gegevens *22*

17.6 Afwijkingen van het protocol *22*

18. Publicaties **23**

19. Administratieve procedures en verantwoordelijkheden **23**

20. Verzekering **23**

21. Referenties **24**

Addenda:

Investigator's Brochure

Informed consent met toestemmingsverklaring

Hoge tonen audiometrie

Questionnaire

Case Report Forms:

1. Screening formulier
2. Controle bezoek formulier
3. Completion pagina
4. Serious Adverse Event (SAE) formulier

3. Lijst met afkortingen

AMP	Antimicrobieel peptide
BPI	Bactericidal permeability increasing protein
COM	Chronische Otitis Media
CME	Commissie Medische Ethiek
CRF	Case report form
ERK	Extracellular signal-related kinase
GCP	Good Clinical Practice
GMP	Good Manufacturing Practice
GNB	Gram-negatieve bacteriën
GPB	Gram-positieve bacteriën
LPS	Lipopolysaccharide
LTA	Lipoteichoid acid
MAP	Mitogen activated phoshate
MCS	Mucociliair clearance systeem
OME	Otitis media met effusie
SADR	Serious adverse drug related
SAE	Serious adverse event

4. Samenvatting

Deze studie heeft tot doel een nieuwe behandelingsmethode voor chronische middenoorontsteking bij volwassenen te testen op veiligheid en werkzaamheid. De nieuwe behandelingsmethode bestaat uit oordruppels met een antimicrobieel peptide. Door het groeiende probleem van bacterie resistentie voor antibiotica is er behoefte aan nieuwe veilige middelen voor de behandeling van bovenste luchtweg infecties. De hypothese in deze studie is dat de nieuwe behandeling resulteert in een verbetering van het middenoorslijmvlies ten opzichte van behandeling met oordruppels zonder antimicrobieel peptide. De studie is voorafgegaan door een dose-finding studie. Hierbij is de veiligheid getest en de optimale dosering van het peptide gezocht. De dubbelblind gerandomiseerde studie heeft tot doelstelling te onderzoeken of de oordruppels met het antimicrobieel peptide effectiever zijn dan placebo oordruppels. De onderzoekspopulatie voor deze studie betreft een groep volwassenen met langdurige middenoorproblemen (chronische otitis media (COM) en een trommelvliesperforatie), die geen baat hebben gehad van eerdere behandelingen met antibiotica. Voor de dose-finding studie zijn steeds 4 deelnemers per concentratie geïncludeerd en voor de hoofdstudie zullen totaal 52 patiënten worden geïncludeerd. De deelnemende proefpersonen zullen gedurende 2 weken 2 maal daags de oordruppels gebruiken. Met behulp van oorinspectie, een CT-scan van het mastoid, een toon-audiogram, bacteriekweek van oor en keel, en bloedanalyse zal de veiligheid en de werkzaamheid vastgelegd worden. Indien mogelijk zal bij aanvang middenoorvocht worden afgenomen voor aanvullend bacteriologisch onderzoek. De patiënten worden na inclusie 12 weken gevolgd. Als eindpunt geldt een herstel van het middenoorslijmvlies zonder secretie en een droge en rustige trommelvliesperforatie. De studie zal volgens GCP- richtlijnen worden uitgevoerd.

5. Inleiding

5.1 Achtergrond

In verband met toenemende antibiotica-resistentie van bacteriën[1,2] is het van groot belang te zoeken naar nieuwe verbeterde behandelingsmogelijkheden. Het door ons ontwikkelde onderzoeksproduct biedt een mogelijk alternatief voor het toenemende gebruik van antibiotica. Met deze gerandomiseerde studie willen we daarom het onderzoeksproduct na toxiciteit- en dosis/effect onderzoek bij volwassenen met middenoorproblemen onderzoeken.

Bijna 75% van alle jonge kinderen heeft minstens één keer middenoorontsteking met ophoping van vocht (effusie) in het middenoor veroorzaakt door chronisch veranderd slijmvlies en een verdikt mucus producerend slijmvlies. Dit gaat soms gepaard met een tijdelijk gehoorsverlies. Meestal gaat de inflammatie vanzelf over, maar als de effusie aanwezig blijft krijgt ongeveer 15% van deze kinderen een chronische middenoorontsteking (OME).[3,4] Hierbij kunnen langdurige gehoorproblemen ontstaan. Tevens kan hierdoor een blijvende schade aan het trommelvlies of de gehoorbeenketen ontstaan. De aandoening kan zich ook bij volwassenen voordoen, al komt dit minder vaak voor dan bij kinderen. Bij volwassenen wordt vaker chronisch veranderd slijmvlies gezien bij otitis media chronica samen met een perforatie van het trommelvlies. Ongeveer 2% van de populatie heeft significante gezondheidsproblemen door chronische otitis media.[5]

5.2 Huidige situatie

Tot op heden worden kinderen met middenoorontsteking vooral behandeld met trommelvliesbuisjes, antibiotica al of niet in combinatie met het verwijderen van de neusamandelen.[3] Nadelen van deze behandelingsmethoden zijn o.a. voortijdig verlies van de buisjes, schade aan het trommelvlies en ontstaan van resistente bacteriën.[6] Ook worden met deze behandelingsmethoden bacterieproducten niet geneutraliseerd zodat de ontstekingsreactie door kan gaan. Veel kinderen krijgen zelfs meer dan éénmalig plaatsing van trommelvliesbuisjes met het risico van een blijvende beschadiging aan het trommelvlies. In geval van succesvolle behandeling met antibiotica komen de klachten vaak binnen 30 dagen na beëindiging van de therapie terug.[7] Volwassenen worden vaak behandeld met antibiotica, en bij trommelvliesperforatie met locale antibiotica, maar in een deel van de patiënten zonder blijvend resultaat. Zelfs chirurgie geeft niet altijd verbetering.

5.3 Pre-klinisch onderzoek

Zowel de bacterieproducten lipopolysaccharide (LPS) als lipoteichoid acid (LTA) kunnen chronische veranderingen van mucosa induceren. Het is gebleken dat LPS een belangrijke rol speelt bij de pathogenese van chronische middenoorontsteking. LPS is aangetoond in middenoorvocht en is tevens in staat gebleken inflammatoire reacties in het middenoor en in de buis van eustachius op te wekken.[8,9,10,11] Wanneer de buis van eustachius slecht functioneert of verstopt is kan overtollig vocht niet meer uit het middenoor verwijderd worden. Dit gebeurt normaal gesproken door het mucociliaire clearance systeem (MCS), wat bestaat uit slijmbekercellen en trilhaar(dragende)cellen. Door beweging van de trilharen kan het slijm met daarin eventueel aanwezige bacteriën of bacterieproducten verwijderd worden uit het middenoor. Als door infectie en/of obstructie van de buis van eustachius het MCS ontregeld raakt blijven producten zoals LPS en LTA in het middenoor aanwezig en kunnen zo de ontstekingsreactie opnieuw induceren zodat een chronische OME en COM ontstaat. Zo ontstaat er een vicieuze cirkel. Om deze te doorbreken is in preklinisch onderzoek onderzocht of het antimicrobieel peptide AMP60.4Ac LPS en LTA kan neutraliseren.

Uit voorgaande studies binnen onze afdeling is gebleken dat neutralizatie van LPS zowel *in vitro* als *in vivo* met behulp van het LPS-neutralizerende bactericidal permeability increasing (BPI) eiwit resulteerde in een herstel van het MCS.[12,13] Effectieve neutralizatie van bacterieproducten ter plaatse biedt daarom mogelijkheden voor behandeling van chronische middenoorproblemen. Het nieuwe door ons ontwikkelde synthetische peptide is effectiever in LPS neutralizatie gebleken dan BPI en is ook werkzaam in de neutralizatie van LTA.

Het groeiende probleem van bacterie resistentie voor antibiotica en de behoefte aan nieuwe antibiotica heeft de interesse gewekt voor de ontwikkeling van antimicrobiële peptiden als humane therapeutica. Verschillende van deze peptiden, afgeleid van menselijke of dierlijke antimicrobiële eiwitten, worden op dit moment voor verschillende toepassingen getest in klinische studies.[14]

5.4 Onderzoeksproduct

AMP60.4Ac is een synthetisch peptide afgeleid van het humane cathelicidin LL-37. LL-37 is een cationisch eiwit aanwezig in neutrofiele granulocyten en epitheelcellen en wordt tevens uitgescheiden door onder andere het epitheel van de luchtwegen en de huid.[15,16] Naast een directe antimicrobiële functie, fungeert LL-37 als mediator van de inflammatie en heeft effecten op epitheel- en ontstekingscellen. Hierbij beïnvloedt het processen als proliferatie, immuun inductie, wond heling, cytokine release, mestcel activatie en chemotaxis.[18,19] LL-37 is betrokken bij de afweerrespons tegen gram-negatieve (GNB) en gram-positieve bacteriën (GPB).[20] Het bindt sterk aan zowel LPS van GNB als aan LTA van GPB en neutraliseert tevens hun pro-inflammatoire activiteit. In muizen leidt deficiëntie van het muizen cathelicidin CRAMP in een verminderde capaciteit om huidinfecties te controleren.[21] Bij de mens gaat een granulocyten stoornis (morbus Kostmann), die leidt tot LL-37 deficiëntie in speeksel, veelal gepaard met peridonditis.[17] LL-37 blijkt dus belangrijk bij de controle van onstekingsreacties. Tijdens inflammatie processen is er een verhoogde LL-37 expressie aangetoond.[22,23] Het werkingsmechanisme, de immunotoxiciteit, de bereiding en opzuivering van het peptide zijn uitgebreid beschreven in de Investigator's Brochure.

5.5 Risico's en voordelen proefpersonen

AMP60.4Ac is in voorafgaand *in vitro* onderzoek in staat gebleken om LPS te neutraliseren en om de door LPS en LTA geïnduceerde cytokine productie in volbloed te remmen (zie Investigator's Brochure). Hieruit blijkt dat *in vitro* de werking van het synthetische peptide vergelijkbaar is met die van het humane LL-37. Bovendien is gebleken dat het synthetische peptide een lagere T-cell proliferatie en minder cytokine productie *in vitro* veroorzaakt dan LL-37. Het lijkt er dus op dat er geen immuunrespons van het peptide is te verwachten. Verder hebben we aangetoond dat het synthetische peptide een lagere activatie van het MAPkinase ERK1/2 (een maat van celactivatie) induceert in bronchiale epitheelcellen. In een chemotaxie bepaling is gebleken dat peptide AMP60.4Ac geen chemotaxis van neutrofiele granulocyten induceert, terwijl dit wel werd aangetoond voor twee andere door ons ontwikkelde synthetische peptiden en voor LL-37. Op basis van bovengenoemd onderzoek worden dus geen negatieve reacties van het onderzoeksproduct verwacht. Dit in tegenstelling tot oordruppels met antibiotica waarvan aangetoond is dat deze in sommige gevallen ototoxisch zouden kunnen zijn.[24,25,26,27] In een onderzoek naar mogelijke toxiciteit van AMP60.4Ac bij ratten zijn geen nadelige effecten gevonden van het peptide. De maximale getolereerde dosis getest in een dosis escalatie studie met ratten was hoger dan 8 mg/kg/dag. De hoogste dosis getest was 8 mg/kg/dag, hierbij zijn geen nadelige effecten gevonden.

Deze nieuwe behandeling kan een verbeterde genezing van chronische OME bewerkstelligen t.o.v. huidige behandelingsmethoden. Bovendien hebben vergelijkbare peptiden geen ontstaan van antibiotica resistentie laten zien, zijn veilig gebleken bij mucosale toediening in zalf (www.genaera.com) en worden momenteel

getest in diverse klinische studies.[28,14] In de fase I studie, waarbij 16 patiënten met therapieresistente chronische otitis media werden onderzocht, werden geen bijwerkingen aangetoond.

5.6 Inbreng participerende instelling

Het LUMC heeft samen met OctoPlus een Letter of Intent opgesteld waarin de samenwerking aangaande dit project is vastgelegd. OctoPlus heeft een ruime expertise op het gebied van drug development en -delivery, en kunnen daarom een belangrijke bijdrage leveren aan dit project.

6. Hypothese / Vraagstelling

Hypothese:

Deze nieuwe behandeling zal een verbeterde genezing van chronische middenoorontsteking met trommelvliesperforatie bij volwassenen bewerkstelligen.

Vraagstelling:

De vraagstelling van de dose-finding studie was om te onderzoeken of applicatie van AMP60.4Ac rechtstreeks in het middenoor van volwassenen met chronische middenoorproblemen met een trommelvliesperforatie:

1. Geen toxische bijwerkingen heeft, en
2. Welke concentratie peptide zal zorgen voor een verbetering van het middenoorslijmvlies richting een droog, plat gezond middenoorslijmvlies.

Bij de dose-finding studie zijn geen ernstige nadelige bijwerkingen gemeten en de optimale dosis is bepaald op 0.5 mg/ml. De vraagstelling die in het tweede deel van de studie onderzocht zal worden is of de nieuwe behandeling een verbetering van het middenoorslijmvlies ten opzichte van de placebo behandeling te zien geeft. In dit gedeelte zal nog steeds de veiligheid van de nieuwe behandeling gecontroleerd worden.

7. Doelstelling

De primaire doelstelling van deze studie is om te onderzoeken of AMP60.4Ac veilig toegediend kan worden op ontstoken slijmvlies van het middenoor bij volwassenen, zonder dat er nadelige bijwerkingen optreden.

De secundaire doelstelling is het onderzoeken of de nieuwe behandeling een verbetering van het middenoorslijmvlies teweegbrengt ten opzichte van een placebo behandeling.

8. Onderzoekspopulatie

De onderzoekspopulatie betreft een groep volwassen patiënten met langdurige middenoorproblemen (chronische otitis media en een trommelvliesperforatie). De beoogde groep patiënten is eerder zonder resultaat behandeld geweest met antibiotica. Tijdens de studie zullen patiënten geen antibiotica gebruiken. Alle medicatie die in het afgelopen halfjaar gebruikt is zal worden geregistreerd in de Case Report formulieren.

Recrutering:

De recrutering voor de dose-finding studie heeft plaatsgevonden uit de bestaande patiëntenpopulatie van de afdeling KNO van het LUMC door de hoofdonderzoeker (behandelend arts) van deze studie. Voor het gerandomiseerde deel van de studie zullen naast patiënten uit het LUMC tevens patiënten gerecruteerd worden in het Academisch Medisch Centrum te Amsterdam en het Erasmus Universitair Medisch Centrum van Rotterdam. De onderzoekers zullen de patiënten informeren over de

voor- en nadelen van deelname aan de studie en zorgdragen dat hun privacy gewaarborgd blijft. Voor deelname aan de studie zullen de deelnemers een informed consent (zie bijlage) ondertekenen.

Inclusie criteria:
-- volwassenen ≥ 18 jaar
-- wilsbekwaam
-- chronische proliferatieve slijmvliesverandering > 6 maanden met duidelijke trommelvliesperforatie
-- adequate behandeling ontvangen voor COM waarbij minimaal 2 episoden van > 6 weken is behandeld met minimaal 2 verschillende oordruppels uit de geselecteerde lijst (antibiotica therapie resistent)

Exclusie criteria:
-- cholesteatoom in het te behandelen oor
-- het bestaan van een radicaalholte in het te behandelen oor
-- antibiotica of corticosteroiden of immunosuppresiva gebruik (locaal en/of systemisch) tot 4 weken voorafgaande aan de studie en tijdens de studie
-- syndroom van Down of andere congenitale afwijkingen aan de gehoorgang of middenoor
-- bekende afweerstoornissen, inclusief auto-immuunstoornissen
-- duizeligheidklachten, ernstige hoofdpijn of facialisafwijkingen
-- zwangerschap
-- eerder inclusie in 'dose finding' studie

Lijst van oordruppels voor behandeling van COM:

Samenstelling	Merknaam
Ofloxacine	Trafloxal
Dexamethason/chlooramfenicol/polymyxine B	Chloorampoldex
Dexamethason/framycetine/gramicidine	Sofradex
Dexamethason/tobramycine	Tobradex
Dexamethason/neomycine/polymyxine B FNA	
Flumetason/clioquinol	Locarten-Vioform
Fluocinolon/neomycine/polymyxine B	Synalar Bi-otic
Hydrocortison/oxytetracycline/polymyxine B	TerraCortril/polymyxine B
Hydrocortison/neomycine/polymyxine B	Otosporin
Hydrocortison/bacitracine/colistine	Bacicoline B
Fludrocortison/neomycine/polymyxine B	Panotile

Controlegroep:
Om het effect van het vehiculum van AMP60.4Ac te onderzoeken wordt er voor de gerandomiseerde studie een controle groep ingesteld, die behandeld worden met de oordruppels zonder peptide (placebo).

Aantal proefpersonen:
Voor het dubbelblind gerandomiseerde deel van de studie is het aantal proefpersonen bepaald op 52 (zie 14.3). Deze worden onderverdeeld in 26 die placebo oordruppels krijgen en 26 die behandeld worden met AMP60.4Ac oordruppels.

9. Onderzoeksopzet

9.1 Ontwerp

De studie is een gerandomiseerd dubbelblind placebo-gecontroleerd onderzoek naar de veiligheid en de werkzaamheid van AMP60.4Ac voor de

behandeling van chronische middenoorontsteking bij volwassenen. Oordruppels met het experimenteel medicament AMP60.4Ac zal versus het vehiculum voor AMP60.4Ac (zie voor omschrijving paragraaf 9.2) onderzocht worden. Momenteel is er geen goede behandelingsmethode voor chronische slijmvliesafwijkingen van het middenoor bij volwassenen. Daarom wordt de studiemedicatie vergeleken met een placebo. Er is gekozen voor een placebo-gecontroleerde studie omdat op deze manier de werking van het vehiculum ook kan worden onderzocht. Omdat het niet ethisch is het te onderzoeken medicament bij gezonde vrijwilligers, die geen trommelvliesperforatie hebben, in het middenoor te brengen voor een fase I veiligheidsstudie, is gekozen voor een gecombineerde fase I/II studie waarbij zowel de veiligheid als de werkzaamheid van AMP60.4Ac bij patiënten onderzocht wordt.

Bij aanvang van de studie zullen de volgende patiëntengegevens verzameld worden:
-- leeftijd
-- chronische slijmvliesafwijkingen met trommelvliesperforatie; links / rechts
-- aspect trommelvlies en middenoorslijmvlies
-- voorafgaande medicatie
-- co-medicatie
-- bovenste luchtweginfecties
-- gehoortest (toon/spraak-audiogram), bij aanvang en bij laatste bezoek tevens hoge tonen audiometrie
-- CT-scan van middenoor en mastoid
-- bacteriekweek van middenoor en keel
-- bloedafname voor bepaling van specifieke peptide antilichamen en algemeen onderzoek (Hb, Ht, BSE, en autom. Diff.)
-- een uit drie onderdelen bestaande questionnaire

Voor de gerandomiseerde dubbelblind gecontroleerde studie worden 52 patiënten geïncludeerd en vervolgens in 2 groepen verdeeld. Hiervan krijgt de eerste groep AMP60.4Ac opgelost in oordruppels, en de tweede groep krijgt als placebo oordruppels zonder AMP60.4Ac.

Randomizatie:
De deelnemers met een éénzijdige proliferatieve slijmvliesverandering met trommelvliesperforatie zullen at random door middel van loting ingedeeld worden in de behandeling- of de placebogroep. Hiertoe zullen de flesjes oordruppels vooraf worden genummerd door een onafhankelijk persoon, die de sleutel bewaart tot aan het einde van het onderzoek volgens de standaard procedures. Bij patiënten met een tweezijdige slijmvliesafwijkingen wordt slechts een van beide oren behandeld.

Blindering:
Er zal een dubbele blindering plaatsvinden, zodat de patiënten zelf en de onderzoekers niet op de hoogte zijn van de ontvangen behandeling.

Stratificatie:
De randomisatie vindt plaats gestratificeerd naar deelnemend centrum middels een gebalanceerd blokken design. De allocatie wordt door de verantwoordelijk statisticus voor de gehele studie van te voren gegenereerd en in electronische vorm aan de leverancier van de de flesjes geleverd.
Ter voorkoming van onbalans dient – gezien de groepsgroottes per centrum – elk centrum blokken geheel vol te maken, zelfs als dat een overschreiding van het totale aantal patienten in de studie ten gevolge zou hebben. Gemiddeld zal per centrum een 18 tal personen worden geincludeerd, zodat een variable blocked design met blokgroottes van 2 en/of 4 tot de mogelijkheden behoren. Als het verwachtte aantal patienten in een centrum kleiner dan 9 is, wordt met een fixed blocksize van 2 gewerkt

9.2 Onderzoeksbehandeling

Product: AMP60.4Ac is een synthetisch peptide afgeleid van het humane cathelicidin LL-37. LL-37 is een cationisch eiwit aanwezig in neutrofiele granulocyten en epitheelcellen, dat antibacteriële eigenschappen heeft. AMP60.4Ac is geproduceerd onder GMP-condities in het door de Farmaceutische Inspectie van IGZ gevisiteerde peptidelaboratorium van de Interdivisionele GMP-faciliteit van het LUMC. Het peptide wordt opgelost in oordruppels (0.02% benzalkoniumchloride, 0.1% dinatriumedetaat, 10% PEG 10.000 in isotone buffer [154 mM NaCl en 20 mM acetaat] pH=5.7) voor optimale afgifte door de slijmlaag heen aan de mucosa. AMP60.4Ac bestaat uit 24 aminozuren met de volgende sequentie: IGKEFKRIVERIKRFLRELVRPLR (geacetyleerd en geamideerd). Mede op basis van de resultaten beschreven in de investigator's brochure hebben we besloten om de klinische studie met peptide 60.4Ac uit te voeren. Alle aminozuren zijn hetzelfde als van peptide 60.4 maar de uiteinden zijn iets veranderd (geacetyleerd en geamineerd) met het oog op extra stabiliteit tegen enzymatische afbraak.

Dosering: enkele druppels (±100 μl) AMP60.4Ac 2x daags gedurende 2 weken via oordruppels op het trommelvlies. Patiënten dienen de oordruppels na instructie zelf thuis toe. Door de trommelvliesperforatie ontstaat een rechtstreekse applicatie op het middenoorslijmvlies van AMP60.4Ac. In de dose-finding studie werd allereerst een optimale dosis voor de behandeling gezocht. Hiertoe werden de peptide concentraties 0.25 / 0.5 / 1.0 en 2.0 mg/ml onderzocht.

Motivering van de dosering: uit het vooronderzoek (zie Investigator's Brochure) naar de werkzaamheid is gebleken dat 1 ng/ml LPS voor 50% geneutraliseerd wordt door 2-4 μg/ml peptide en 100% neutralisatie word bereikt bij een concentratie van 5-8 μg/ml; veiligheidshalve gaan we uit van ≈ 10 μg/ml (ofwel: 10.000X overmaat). In het oor is een concentratie van gemiddeld 1.5, 18 of 96 ng/ml LPS aanwezig afhankelijk van het type middenooreffusie.[11] Als we uitgaan van een concentratie van 100 ng/ml dan zouden we met een concentratie van 1 mg/ml Peptide Solution het aanwezige LPS in het oor kunnen neutraliseren. Uit de dose-finding studie is de optimale concentratie bepaald op 0.5 mg/ml.
In de dose-finding studie is een linear mixed model gefit aan de onderzoeksgegevens waarbij de 4-punts-schaal voor het kwantificeren van het otoscopisch beeld is gebruikt. Een kwadratisch model voor de relatie tussen concentratie en het otoscopisch beeld werd als uitgangspunt gekozen en getoetst werd of de kwadratische term significant was. Dit was niet het geval zodat geconcludeerd moest worden dat binnen het gegeven meetbereik voor de concentraties (0.25, 0.5, 1 en 2) er geen optimum geschat kon worden. De lineaire term was significant, waarbij een klinisch relevant verschil ten gunste van de beide laagste doses gevonden werd. Het verschil tussen de beide laagste doseringen was:

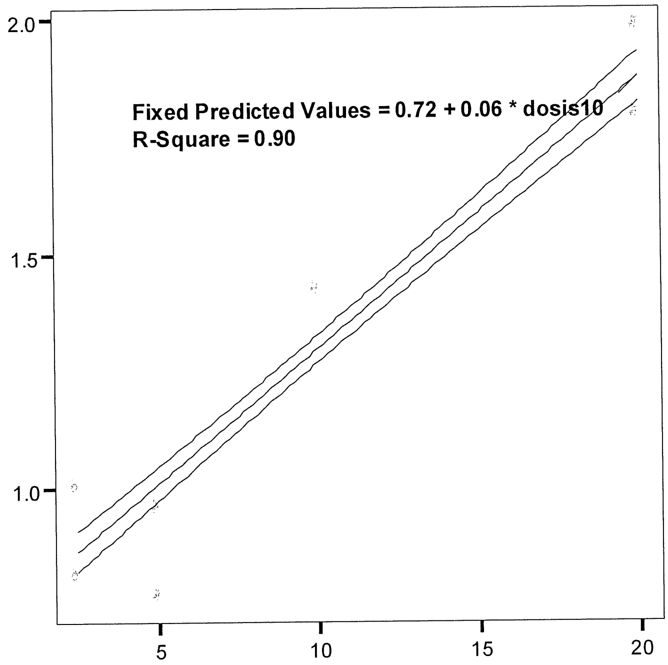

Horizontaal staat de gegeven dosis*10. Het verschil tussen de doses 0.5 en 0.25 is (geobserveerd zowel als voorspeld) minimaal zodat op klinisch-inhoudelijke gronden voor de dosis van 0.5 gekozen is.

Behandelingsperiode: Voor alle deelnemers aan zowel de dose-finding, als ook aan de gerandomiseerde studie geldt dat op week 1, 2, 4, 8 en 12 inspectie van het trommelvlies en het middenoor zal plaatsvinden De toestand van het middenoor en het mastoid wordt tevens bekeken m.b.v. een CT-scan aan het begin en het eind van de studie.

Doseringsschema: 2x daags enkele oordruppels (ca. 100 µl), gedurende 2 weken

Doseringsvorm: Vloeibaar

Verpakking, etikettering: druppelflesje, 2x daags twee druppels, gedurende 2 weken.

Toegestane / niet toegestane medicatie: geen antibiotica toegestaan, overige medicatie in overleg met behandelend arts

Duur van het onderzoek: 12 weken na eerste toediening

Instructies: Op het etiket van de oordruppels komen duidelijke instructies en ook krijgt de patiënt schriftelijke en mondelinge instructies van de behandelend arts.

Verantwoording drugaccountability: Hoeveelheid AMP60.4Ac dat zal worden gebruikt in de studie wordt gedocumenteerd en gecontroleerd door de onderzoekers en de relevante secties binnen de afdeling klinische Farmacie en Toxicologie.

9.3 Schematisch diagram

Stap 1: Bezoek van de patiënt aan de onderzoeker
Procedure: Beoordeling in- en exclusie criteria
⇓
Inclusie in de studie, randomizatie (niet bij de dose-finding)
⇓
Invullen screening formulier van het case report form (CRF)
Inspectie trommelvlies/middenoorslijmvlies
Maken toon-audiogram+hoge tonen audiometrie
Maken CT-scan mastoid
Afname van middenoor- en keelkweek
Afname bloed (1 stolbuis voor spec.antilich.bep. en 1 buis voor Hb,Ht, BSE, en diff.)
⇓
Stap 2: Behandeling
Procedure: Start medicatie AMP60.4Ac / placebo (week 0)
⇓
Stap 3: Controle bezoeken bij de onderzoeker in week 1, 2, 4, 8 en 12
Procedure: invullen controle bezoek formulier van het CRF
Inspectie trommelvlies/middenoorslijmvlies
Maken toon-audiogram
⇓
Stap 4: Einde van de studie in week 12
Procedure: Invullen completion pagina van het CRF
Inspectie trommelvlies/middenoorslijmvlies
Maken toon-audiogram+hoge tonen audiometrie
Maken CT-scan mastoid
Afname van middenoor- en keelkweek
Afname bloed (wk 8, 1 stolbuis voor spec.antilich.bep. en 1 buis voor Hb,Ht, BSE, en diff.)

9.4 Behandelschema

	Week 0 Visite 1	Week 1 Visite 2	Week 2 Visite 3	Week 4 Visite 4	Week 8 Visite 5	Week 12 Visite 6
Beoordeling In- Exclusie criteria	X					
Toestemmingsverklaring	X					
Demografische data	X					
Medische geschiedenis	X					
Gebruik co-medicatie	X	X	X	X	X	X
Lichamelijk onderzoek	X					
CT-scan	X					
Bloedafname	X				X	
Afnemen Oorkweek	X					X
Afnemen Keelkweek	X					X
Beoordelen trommelvliesaspect	X	X	X	X	X	X
Stadium middenoor-slijmvlies	X	X	X	X	X	X
Digitale foto (optioneel)	X	X	X	X	X	X
Audiogram	X	X	X	X	X	X
Hoge tonen audiometrie	X					X
Invullen questionnaires	X	X	X	X	X	X

9.5 Duur en omvang van het onderzoek

Tijdschema hoofdstudie:

	2005 Mei	Juni	Juli	Aug-Dec	2007 Jan-Maart	April	Mei	Juni	2007 Juli-Aug	Sep	Dec
Afronden dose-finding	XX	XX	XX	XX							
Bepalen doses				X							
Start hoofdstudie					X						
Recrutering						XXXX	XXXX	XXXX	XXXX		
Follow-up							XXXX	XXXX	XXXX	XXXX	
Interim-analyse										XXXX	
Invoeren gegevens							XX	XXXX	XXXX	XXXX	
Disclosure sleutel											X
Table and listings											XXXX

Logistieke procedures:
De arts-onderzoeker/behandelaar zal de patiënten recruteren en de afspraken maken voor de geplande onderzoeken. Na eventuele insluiting zullen de proefpersonen op de

geplande visites onderzocht worden door de arts-onderzoeker. Het toon-audiogram zal door een audiometrie-assistent worden afgenomen. Voor de CT-scan zal een afspraak worden gemaakt op de betreffende afdeling.
De monitor zal toezien op de juiste invulling van de case report formulieren. De gegevens zullen worden ingevoerd in een database door de afdeling datamanagement en statistiek. Na disclosure van de sleutel zullen de resultaten door de onderzoekers worden uitgewerkt.
Een monitor zal tussentijds en aan het eind van het onderzoek een controle verrichten op het uitvoeren van de procedures door de onderzoekers.

10. Eindpunten

10.1 Primaire en secundaire eindpunten:

Het primaire eindpunt van de studie zal zijn dat er geen toxische bijwerkingen als gevolg van de behandeling worden gevonden.

Het secundaire eindpunt is het herstel van het middenoorslijmvlies en het trommelvlies.

10.2 Meetmethoden

Veiligheidsaspecten worden geëvalueerd door middel van:
-- inspectie van het oor
-- controle van het gehoor mbv een toon-audiogram en hoge tonen audiometrie
-- bacteriekweek van oor- en keel swabs.
-- bloed analyse
-- detectie van eventuele peptide specifieke antilichamen

De parameters waarmee de werkzaamheid van de behandeling zal worden geëvalueerd zijn:
-- aspect trommelvlies en middenoorslijmvlies d.m.v. oorinspectie
-- subjectieve verbeteringen gemeten mbv questionnaire (Quality of Life).

11. Methode van meten

Het audiogram zal poliklinisch worden gemaakt voorafgaand aan de behandeling en op week 1, 2, 4, 8 en 12 na behandeling door de onderzoeker. De oorinspectie dient om de werkzaamheid in kaart te kunnen brengen. Het otoscopisch beeld wordt beschreven en gekwantificeerd in vier stadia:
0=plat, droog middenoorslijmvlies
1=plat, nat middenoorslijmvlies
2=verdikt polypeus, droog of nat middenoorslijmvlies
3=verdikt polypeus, middenoorslijmvlies met taai slijm

Het toon-audiogram en de hoge tonen audiometrie dient als veiligheidsparameter om eventuele gehoorbeschadiging door de studiemedicatie te kunnen meten. De CT-scan zal alleen bij aanvang worden uitgevoerd om tekenen van ontstekingsreacties in het mastoid te kunnen detecteren. Zodat er zekerheid ontstaat wat betreft in- en exclusie criteria. Resultaten worden vastgelegd in het patiëntendossier en in het CRF.

Aanvullend onderzoek:
Indien mogelijk wordt er bij aanvang van de studie middenooreffusie afgenomen voor een bacteriekweek. Dit om inzicht te verkrijgen in de bacteriologie van otitis media chronica bij volwassenen. Vooraf en na afloop van de studie zal er naast de middenoorkweek ook een keelkweek uitgevoerd worden om een eventuele

verschuiving in de populatie micro-organismen vast te stellen. Tenslotte zal er vooraf en op week 8 in de studie bloed worden afgenomen voor een algemene bloedanalyse en een bepaling van eventuele antilichamen die zich tegen het peptide gevormd zouden kunnen hebben.

12. Lichaamsmaterialen

Alle lichaamsmaterialen zullen worden gecodeerd. De uitslag van de bacteriekweek en het bloedonderzoek kan dan na afloop van de studie gekoppeld worden aan de bijbehorende patiënt. Het materiaal is zo dus indirect herleidbaar tot de betreffende proefpersoon. De sleutel van de code is in handen van de behandelaar. Alleen deze kan de uitslag van de bepaling na afloop van de studie aan de betreffende persoon koppelen. Na afloop van het onderzoek is het materiaal 'op' of wordt het resterende materiaal vernietigd.

Over de afname van het materiaal wordt de proefpersoon zowel mondeling als schriftelijk geïnformeerd. Hierbij wordt ook vermeld op welke wijze het materiaal wordt verwerkt. Met het tekenen van de toestemmingsverklaring geeft de proefpersoon toestemming voor de afname en verwerking van het materiaal. Dit gebeurt in het kader van het onderzoek.

13. Methode van uitvoering / Laboratoriumwerkwijze

Voor nader wetenschappelijk onderzoek naar de bacteriologie van chronische otitis media met effusie bij volwassenen, zal een bacteriekweek van middenooreffusie worden uitgevoerd. Tevens zal vooraf en na afloop van de studie een middenoor- en keelkweek uitgevoerd worden voor de bepaling van eventuele verschuiving in micro-organisme populatie. Deze bacteriekweek zal volgens standaard protocol in het desbetreffende ziekenhuis bij de afdeling bacteriologie worden gedaan. Tenslotte zal vooraf en in week 8 van de studie een bloedmonster worden afgenomen voor analyse. Hierbij zal de bepaling van het algemeen bloedbeeld (Hb, Ht, differentiatie analyse) in het desbetreffende ziekenhuis worden uitgevoerd en de specifieke peptide-antilichaam bepaling zal in het LUMC gedaan worden. Het hiervoor benodigde serum zal in het desbetreffende ziekenhuis worden bewaard bij −20°C en door de monitor worden meegenomen naar het LUMC voor analyse.

14. Statistische analyses

14.1 Statistische methoden

Het otoscopisch beeld wordt beschreven en gekwantificeerd in vier stadia:
0=plat, droog middenoorslijmvlies
1=plat, nat middenoorslijmvlies
2=verdikt polypeus, droog of nat middenoorslijmvlies
3=verdikt polypeus, middenoorslijmvlies met taai slijm

Geanalyseerd zal worden hoeveel patiënten met de therapeutische behandeling een verbetering van het middenoorslijmvlies hebben na afloop van de studie vergeleken met de controle groep. Enerzijds zullen hiertoe de gemiddelde scores worden vergeleken, zowel ten tijde van de laatste meting (week 12) als het verloop in de tijd (repeated measures analysis of variance met de 0-meting als covariaat ter verhoging van de power). Daarnaast zal ook het percentage proefpersonen met een duidelijke verbetering bij week 12 worden vergeleken als binaire uitkomst maat, waarbij een duidelijke verbetering wordt gedefinieerd als ten minste 2 stappen richting normaal plat, droog middenoorslijmvlies (derhalve 3->1, 3->0 of 2->0). Deze laatste analyse ligt ten grondslag aan de sample size berekening.

14.2 Statistische analyse

De statistische analyse zal uitgevoerd worden door de biostatisticus R. Brand van de afdeling Medische Statistiek en Advanced Data Management. De vergelijking van de gemiddelde scores op week 12 alsmede de vergelijking van het verloop van de scores gelden als analyses van de primaire uitkomst maat. Datzelfde geldt ook voor de vergelijking van het percentage personen met een "duidelijke verbetering". De eerste analyses zijn parametrische analyses, de laatste een logistische regressie-analyse waarbij de randomisatie-groep als groepsindeling, het al dan niet hebben van een "duidelijke verbetering" als uitkomst en andere factoren voor zover bepaald voorafgaande aan het moment van randomisatie als covariaten meegenomen (kunnen) worden, zulks met het doel de power van de groeps vergelijking te verhogen.
De kwaliteit van leven vragenlijsten zullen worden geanalyseerd volgens, indien aanwezig, internationale gestandaardiseerde specificaties, door het resultaat van de scores te vergelijken tussen de twee gerandomizeerde groepen. Aandacht zal worden besteed aan de verschillen in gemiddelde scores en maar ook aan de verdeling van de individuele scores.

14.3 Aantal proefpersonen

Het aantal proefpersonen voor deze studie is berekend met het software pakket East (versie 3.1.0) waarin de mogelijkheid van interim-analyses met ene alpha en beta spending functie wordt geboden. De East software wordt ook gebruikt door de FDA bij de evaluatie van clinical trial resultaten en is gebaseerd op internationaal erkende biostatistische methodes.
Er is gekozen voor een sample size bereking op basis van de vergelijking van twee proporties waarbij er is uitgegaan dat een kans van slagen van de controle behandeling (placebo) van 20% en een slagingskans van de te onderzoeken behandeling van 60% te onderscheiden moeten zijn met een power van 90% bij een tweezijdige toetsing met significantie niveau alpha van 5%.

Er is gekozen voor 1 interim analyse bij een informatie-fractie van 50% (d.w.z. nadat 50% van het benodigde aantal proefpersonen de meting van week 12 heeft bereikt). Bij een zeer snelle accrual zal de interim-analyse niet tot een reductie van het benodigde aantal proefpersonen kunnen leiden (er zijn nog 12 weken accrual te gaan nadat 50% van het aantal proefpersonen is geincludeerd). Mocht de accrual langzamer gaan dan verwacht, dan ontstaat de volgende situatie:

De trial krijgt zowel power voor efficacy als futility; we gebruiken een benadering van de O'Brien-Fleming boundaries in de context van alpha en beta spending. Het maximum aantal benodigde proefpersonen is 52 (2 maal 26); onder de nul-hypothese is het verwachtte aantal 49 (waarbij de uitkomst "futility" kan worden vastgesteld; onder H1 is dit 46 (waarbij de uitkomst "efficacy" wordt vastgesteld); en halverwege H0 en H1 (dwz een verschil van 0.2 ipv 0.4) mogen we totaal 50 inclusies verwachten.

De volgende tabel geeft de kansen weer om de studie bij interim analyse reeds te kunnen beeindigen:

Information Fraction	Cumulative Accrual	Alpha Spent	Beta Spent	Stopping Probabilities		
				Under H0	Under H1	Under H1/2
0.500	26.579	0.003	0.011	0.16	0.27	0.12
1.000	53.157	0.050	0.100	0.84	0.73	0.88

Hieruit blijkt dat bij analyse na 26 evalueerbare personen, onder aanname dat beide groepen hetzelfde resultaat vertonen(H0) er 16% kans is de studie reeds wegens "geen verschil" te beeindigen. Zo is er 27% kans om de studie wegens een significant

verschil te kunnen afsluiten na 26 personen als in werkelijkheid het verschil inderdaada 60 versus 20% is.

Door deze interim analyse wordt derhalve een substantiele kans op kostenbesparing ingebouwd in het design.

Indien bij interim analyse de trial gestopt wordt, gaat de follow-up van de reeds geincludeerde personen gewoon door. Echter, de 12-weeks data van de personen die niet in de interim analyse betrokken waren, kunnen NIET meer voor een eind-analyse gebruikt worden omdat de interim-analyse reeds als eindanalyse geldt en een herhaling van deze analyse ondanks het feit dat de van te voren vastgestelde stopping-boundaries zijn overschreven, een niet-acceptabele verandering van de voor-ingestelde alpha en beta geven.
Dit laat onverlet dat men wel de gegevens van de complete voor safety doeleinden mag en moet gebruiken; echter, de conclusie betreffende het efficacy aspect is reeds getrokken.

15. Uitkomsten/bevindingen en hun implicaties

De uitkomsten van het onderzoek zullen worden uitgedrukt in score van het middenoorslijmvlies, aantallen genezen patiënten, Quality of Life en safety. De resultaten zullen worden gepubliceerd in internationale wetenschappelijke tijdschriften.
Na afloop van de studie zullen de deelnemende proefpersonen worden geïnformeerd over de individuele en de algemene resultaten.

16. Ethische overwegingen

Deze studie zal naar verwachting leiden tot nieuwe inzichten op het gebied van de behandeling en de bacteriologie van chronische otitis media bij volwassenen. Deze nieuwe inzichten kunnen tevens belangrijk zijn voor de verdere ontwikkeling van behandelingen voor andere (bovenste) luchtweg infecties en otitis media met effusie bij kinderen. Daarom achten wij het belang van het onderzoek evenredig aan de bezwaren en risico's. De verwachtte resultaten kunnen niet verkregen worden zonder deze patiëntenstudie waarvan de leiding en uitvoering in handen zal zijn van deskundigen. Bovendien zal de persoonlijke levenssfeer beschermd worden en zal het belang van de deelnemers prevaleren boven het belang van de wetenschap of de samenleving.

17. Controle vordering van het onderzoek

17.1 Beoordeling van de veiligheid

De belangrijkste veiligheidsparameter is het toon-audiogram. Hiermee kan een eventueel opgetreden perceptief gehoorsverlies aangetoond worden. De test zal voorafgaand aan de behandeling en op week 1, 2, 4, 8 en 12 na de behandeling poliklinisch worden afgenomen door een audiometrie-assistent. Resultaten worden vastgelegd in het audiogram dat wordt opgenomen in het patiëntendossier en in het CRF. Verder zal door middel van bacteriekweek van middenoor en keel en bloedanalyse de veiligheid van het peptide extra gecontroleerd worden.

17.2 Beëindiging van het onderzoek en (serious) adverse events (SAEs)

Een ernstig ongewenst voorval (SAE) of ernstige nadelige bijwerkingen (SADR) worden gedefinieerd als elk ongewenst medisch voorval dat bij ongeacht welke dosis:

de dood tot gevolg heeft, levensbedreigend is, ziekenhuisopname of verlenging van opname noodzakelijk maakt, tot blijvende of ernstige invaliditeit en / of tot arbeidsongeschiktheid leidt of een aangeboren afwijking/geboorteafwijking is.

Stopzetten van het onderzoek:
-- als er onverwachte SADRs zijn bij meer dan 6 patiënten
-- als er SAEs optreden bij meer dan 6 patiënten
-- in overleg met de CME

Criteria voor beëindiging onderzoek individuele patiënten:
-- als er een SAE of een SADR optreedt waarbij de patiënt opgenomen moet worden in het ziekenhuis.
-- als patiënt plotseling doof wordt, of er een toenemend perceptief gehoorsverlies is.
-- bij duizeligheid, facialis afwijkingen of ernstige hoofdpijn.
-- non-compliance van de patient

Rapportage van (serious) adverse events en ziekten tijdens het onderzoek zal plaatsvinden in het patiëntendossier en in het CRF. De duur en nazorg bij (serious) adverse events is in handen van de behandelend arts. De betreffende proefpersoon zal de studie beëindigen. SAE's zullen onmiddellijk gemeld worden aan de proefpersoon, de studie-coordinator en aan de CME en voorzien van commentaar van de onderzoeker.

Procedure uit het onderzoek halen van patiënten:
-- invullen de completion pagina van het CRF, indien mogelijk laatste gegevens verzamelen
-- nazorg verlenen indien noodzakelijk
-- vervanging van de proefpersoon in het onderzoek

17.3 Monitoring Committee

Vanuit de participerende instelling zal een studiemonitor worden aangewezen, die zal controleren of
-- de formulieren goed worden ingevuld
-- er goed wordt gehandeld in geval van SAEs

17.4 Interim-analyses

Op de helft van de hoofdstudie zal er een interim-analyse door de statisticusplaatsvinden. Wanneer bij interim-analyse blijkt dat er bij meer dan 4 deelnemers van de onderzoeksgroep:
-- een serious adverse event is opgetreden waarbij de patiënt opgenomen moest worden
-- plotseling doofheid optreedt, of er een toenemend perspectief gehoorsverlies is
-- duizeligheid, facialis afwijkingen of ernstige hoofdpijn ontstaan
zal het onderzoek beëindigd worden.

Zoals onder 14.3 vermeld, vindt er na 26 evalueerbare proefpersonen een formele interimanalyse ten aanzien van de primaire uitkomstmaat plaats. De trial zal dan gestopt worden indien uit deze analyse conform specificaties de nul-hypothese van gelijkheid van behandeling bij de dan geldende kritieke grens verworpen kan worden (efficacy) dan wel de alternatieve hypothese van een verschil tussen de behandeling verworpen kan worden (futility).
Het volgende schema geeft deze beslisgrenzen weer: de eerste figuur geeft de kritieke grenzen op de p-waarde schaal weer; de tweede grafiek vertaalt deze in gevonden verschillen tussen de behandelingen.

p-waarden:

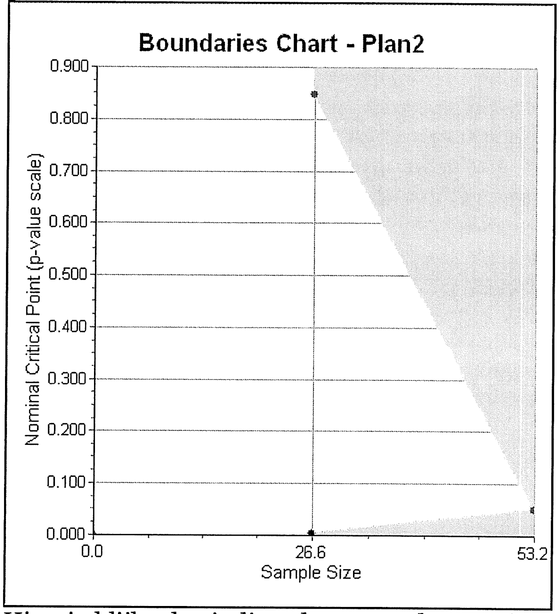

Hieruit blijkt dat indien de p-waarde van de toets welke de proporties duidelijke vooruitgang vergelijkt tussen beide groepen bij interim analyse 0.85 of hoger is, de trial gestaakt wordt wegens futility. Als de p-waarde onder de 0.003 ligt bij interim analyse, wordt geconcludeerd tot verschil van de behandelingen en de trial ook gestopt. Met deze methode blijven de overall alpha en beta op hun oorspronkelijke niveau van 5% resp 90%.

In termen van actuele verschillen zijn deze boundaries als volgt:

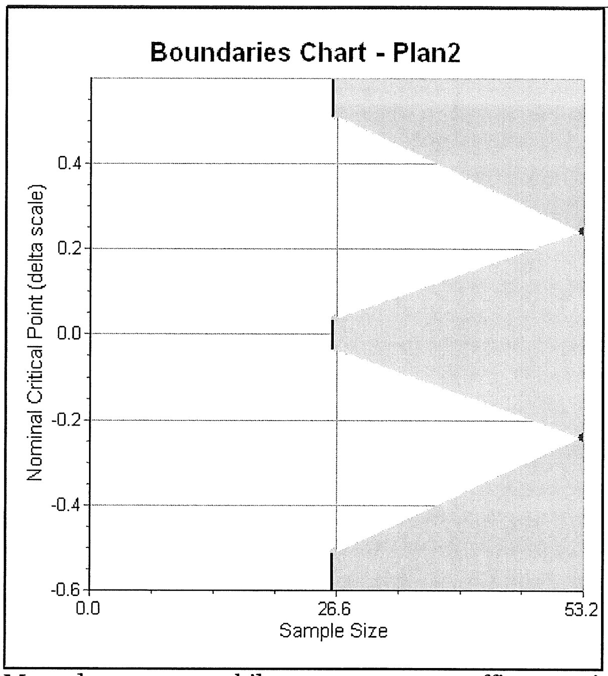

Meer dan 0.51 verschil: stoppen wegens efficacy; minder dan 0.34 verschil stoppen wegens futility (0.51=51% verschil in success percentage)

De error spending bij dit design ziet er als volgt uit:

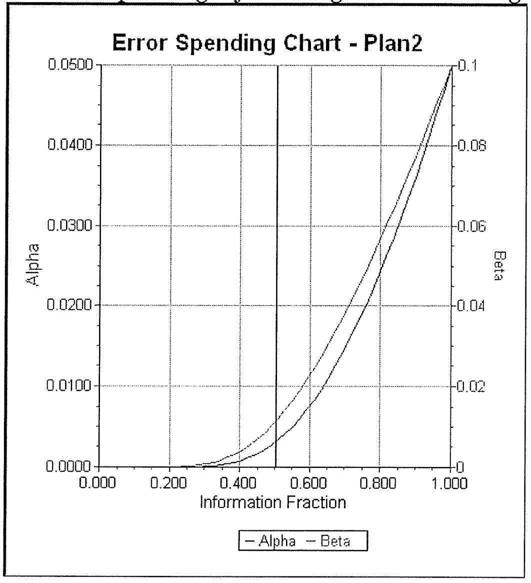

De power van de studie hangt als volgt af van de gemaakte aannames:

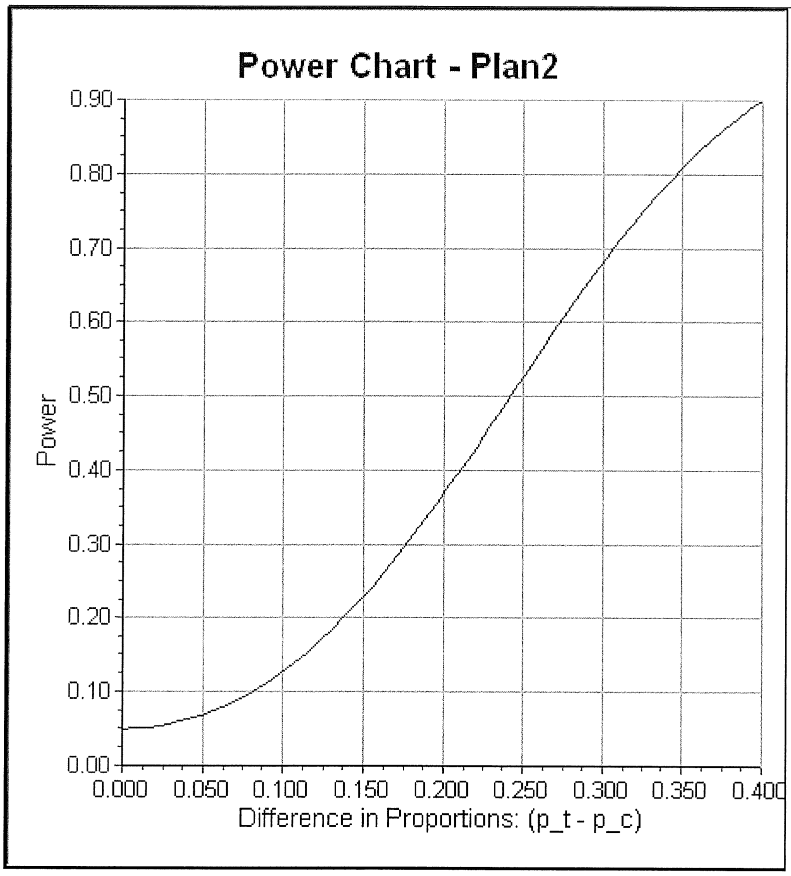

Hieruit blijkt dat als het verschil niet de gepostullerde 40% (60-20) is maar 35%, er nog steeds een power van 80% is om het verschil als significant te detecteren. Bij een verschil in effectiviteit van minder dan 30% is de power niet meer voldoende.

17.5 Studie formulieren en gegevens

De gegevens van de studie zullen worden opgenomen in het patiëntendossier en in de speciale case report formulieren, welke vertrouwelijk zullen worden behandeld. Alleen de onderzoekers, de monitor, de auditor, de ethische commissie, bevoegde autoriteit en de participerende instelling hebben hierin inzage en in de brongegevens. Op de case report formulieren zal alleen een gecodeerd patiënten nummer vermeld worden, zodat er geen directe herleiding is met de proefpersoon.

17.6 Afwijkingen van het protocol

Alle gerandomiseerde proefpersonen zullen worden meegenomen in de intention-to-treat analyse, welke als primaire analyse geldt, zowel qua safety als qua efficacy. Daarna zal een per-protocol analyse naar de efficacy worden uitgevoerd waarbij protocol-violations (ten onrechte gedane inclusies cq ernstige protocol-afwijkingen) buiten beschouwing worden gelaten. Voor de safety analyses worden derhalve nooit patienten uitgesloten.

Rapporteren van afwijkingen van het oorspronkelijke ontwerp:
Afwijkingen zullen d.m.v. een amendement aan het protocol worden toegevoegd. Wijzigingen van het oorspronkelijke protocol worden pas doorgevoerd na goedkeuring van de CME.

18. Publicaties

Na afloop zullen de studieresultaten worden gepubliceerd. Indien relevant zal publicatie van resultaten met de gebruikte studiemedicatie pas plaatsvinden als deze gepatenteerd is. De participerende instelling (OctoPlus NV) heeft daarna voorinzage gedurende een maximum van 6 maanden, maar kan de publicatie niet tegenhouden.

19. Administratieve procedures en verantwoordelijkheden

Codering van proefpersoongegevens, bewaren code lijst
In een code lijst zal worden vastgelegd welke behandeling de deelnemers op volgorde van binnenkomst krijgen.

Verbreken blindering
Na afloop van de studie zal de met behulp van de code lijst gekeken worden welke behandeling de deelnemers hebben ontvangen zodat de resultaten kunnen worden uitgewerkt.

Procedure wijzigen protocol
Een wijziging van het protocol zal na toestemming van alle betrokkenen ter goedkeuring aan de CME worden voorgelegd. Na goedkeuring zal de wijziging als addendum aan het protocol worden toegevoegd.

20. Verzekering

De verzekering van de proefpersonen zal door de Sponsor geschieden.

21. Referenties

1. Klein, J. O. 2000. Clinical implications of antibiotic resistance for management of acute otitis media. *J Lab Clin Med* 135:220-224.

2. Froom, J., L. Culpepper, M. Jacobs, R. A. DeMelker, L. A. Green, L. van Buchem, P. Grob, and T. Heeren. 1997. Antimicrobials for acute otitis media? A review from the international primary care network. *BMJ* 315:98-102.

3. Klein, J. O. 1994. State-of-the-Art Clinical Article. *Clinical Infectious Diseases* 19:823-833.

4. Faden, H., L. Duffy, and M. Boeve. 1998. Otitis media: back to basics. *Pediatr.Infect.Dis.J.* 17:1105-1113.

5. Nadol, J. B., H. Staecker, and R. E. Gliklich. 2000. Outcomes assessment for chronic otitis media: The chronic ear survey . *Laryngoscope* 110:32-35.

6. Schilder, A. G. M., G. A. Zielhuis, M. P. Haggard, and P. Broek van den. 1995. Long-term effects of otitis media with effusion: otomicroscopic findings. *Am.J.Otology* 16:365-372.

7. Klein, J. O. 1994. Lessons from recent studies on the epidemiology of otitis media. *Pediatr.Infect.Dis.J.* 13:1031-1034.

8. Nell, M. J. and J. J. Grote. 1999. Structural changes in the rat middle ear mucosa due to Endotoxin and Eustachian Tube Obstruction. *Eur.Arch.Otorhinolaryngol.* 256:167-172.

9. DeMaria, Th. F., T. Yamaguchi, and D. J. Lim. 1989. Quantitative cytologic and histologic changes in the middle ear after injection of nontypable *Hemophilus influenzae* endotoxin. *Am.J.Otolaryngol.* 10:261-266.

10. Nell, M. J., B. M. Op 't Hof, H. K. Koerten, and J. J. Grote. 1999. Effect of endotoxin on cultured human middle ear epithelium. *ORL* 61:201-205.

11. Nell, M. J. and J. J. Grote. 1999. Endotoxin and TNF-alpha in middle ear effusions: in relation with upper airway infection. *Laryngoscope* 109:1815-1819.

12. Nell, M. J., B. M. Albers-Op 't Hof, H. K. Koerten, and J. J. Grote. 2000. Inhibition of endotoxin effects on cultured human middle ear epithelium by Bactericidal/Permeability-Increasing protein. *Am.J.Otology* 21:625-630.

13. Nell, M. J., H. K. Koerten, and J. J. Grote. 1999. Bactericidal/permeability-increasing protein prevents mucosal damage in an experimental rat model of chronic otitis media with effusion. *Infection and Immunity* 68:2992-2994.

14. Zasloff, M. 2002. Antimicrobial peptides of multicellular organisms. *Nature* 415:389-395.

15. Bals, R., X. Wang, M. Zasloff, and J. M. Wilson. 1998. The peptide antibiotic LL-37/hCAP-18 is expressed in epithelial of the human lung where it has broad antimicrobial activity at the airway surface. *Proc.Natl.Acad.Sci.USA* 95:9541-9546.

16. Agerberth, B., J. Grunewald, E. Castaños-Velez, B. Olsson, H. Jörnvall, H. Wigzell, A. Eklund, and G. H. Gudmundsson. 1999. Antibacterial components in bronchoalveolar

lavage fluid from healthy individuals and sarcoidosis patients. *Am.J.Respir.Crit.Care Med.* 160:283-290.

17. Pütsep, K., G. Carlsson, H. G. Boman, and M. Andersson. 2002. Deficiency of antibacterial peptides in patients with morbus Kostmann: an observation study. *The Lancet* 360:1144-1149.

18. Bals, R. 2000. Epithelial antimicrobial peptides in host defense against infection. *Respir Res* 1:141-150.

19. Yang, D., O. Chertov, and J. J. Oppenheim. 2001. Participation of mammalian defensins and cathelicidins in anti-microbial immunity: receptors and activities of human defensins and cathelicidin (LL-37) . *J.Leukocyte Biology* 69:691-697.

20. Hancock, R. E. W. and G. Diamond. 2000. The role of cationic antimicrobial peptides in innate host defences. *Trends in Microbiology* 8:402-410.

21. Nizet, V., T. Ohtake, X. Lauth, J. Trowbridge, J. Rudisill, R. A. Dorschner, V. Pestonjamasp, J. Piraino, K. Huttner, and R. L. Gallo. 2001. Innate antimicrobial peptide protects the skin from invasive bacterial infection. *Nature* 414:454-457.

22. Frohm, M., B. Agerberth, G. Ahangari, M. Ståhle-Bäckdahl, H. Wigzell, and G. H. Gudmundsson. 1997. The expression of the gene coding for the antibacterial peptide LL-37 is induced in human keratinocytes during inflammatory disorders. *J.Biol.Chemistry* 272:15258-15263.

23. Paulsen, F., T. Pufe, L. Conradi, D. Varoga, M. Tsokos, J. Papendieck, and W. Petersen. 2002. Antimicrobial peptides are expressed and produced in healthy and inflamed human synovial membranes. *J.Pathol.* 198:369-377.

24. Marais, J. and J. A. Rutka. 1998. Ototoxicity and topical eardrops. *Clin.Otolaryngol.* 23:360-367.

25. Lundy, L. B. and M. D. Graham. 1993. Ototoxicity and ototopical medications: a survey of otolaryngologists. *Am.J.Otology* 14:141-146.

26. Russell, N. J., K. E. Fox, and R. E. Brummett. 1979. Ototoxic effects of the interaction between kanamycin and ethacrynic acid. *Acta Otolaryngol.* 88:369-381.

27. Tange, R. A. and E. H. Huizing. 1980. Hearing loss and inner ear changes in a patient suffering from severe gentamicin ototoxicity. *Arch Otorhinolaryngol* 228:113-121.

28. Lehrer, R. I. and T. Ganz. 2002. Cathelicidins: a family of endogenous antimicrobial peptides. *Current Opinion in Hematology* 9:18-22.

LUMC

1. Handtekeningenblad

De hieronder genoemde betrokkenen van het LUMC en de participerende instelling verklaren hierbij akkoord te gaan met dit studieprotocol.

Drs. F.A.W. Peek
Hoofdonderzoeker

15/8/7

Dr. J.W. Drijfhout
Immunohematologie en Bloedtransfusie (IHB)

Prof. Dr. P.S. Hiemstra
Longziekten

Drs. G. Slappendel
Apotheker

15/08/2007

E. van Hoogdalem, PhD, RPh
Chief Medical Officer, Octoplus Technologies B.V.

Investigator: N.A.
Site: N.A.
