# Supplementary material for: Ototopical drops containing a novel antibacterial synthetic peptide: Safety and efficacy in adults with chronic suppurative otitis media
Source: PLoS One. 2020 Apr 14;15(4):e0231573. doi: 10.1371/journal.pone.0231573 (PMC7156094; doi:10.1371/journal.pone.0231573)
Supplement: S2 Protocol — (DOCX) [file pone.0231573.s006.docx]

1. **ETHICS**
   1. **INDEPENDENT ETHICS COMMITTEE**

The clinical study protocol and protocol amendments were approved by the Independent Ethics Committee (IEC) of the Leiden University Medical Center (LUMC).

- 1. **ETHICAL CONDUCT OF THE STUDY**

The study was conducted in accordance with the ethical principles that have their origins in the Declaration of Helsinki.

- 1. **SUBJECT INFORMATION AND CONSENT**

Before enrolment in the study, the subjects were given an explanation of the nature, purpose, possible risks and benefits of the study and they were informed that their privacy would always be guaranteed. Subjects were also informed on the nature of the tests to be performed and the fact that body material/fluids would be collected. By signing the informed consent form, the subject consented to collection, handling and storage of these samples for the purposes of the study.

All subjects signed an informed consent form before any study specific procedures were performed.

1. **INVESTIGATORS AND STUDY ADMINISTRATIVE STRUCTURE**
   1. **INVESTIGATORS**

The study was coordinated by the LUMC, Albinusdreef 2, 2333 ZA Leiden, The Netherlands.

The investigators of the principle site were:

- Drs. F.A.W. Peek (principal investigator (PI)) and Drs. J.P.R. van Merkesteyn (Independent Physician)
  LUMC, Ear Nose Throat (ENT) Department
  Tel.: +31 24 361 0353

There were nine other Dutch centers that participated in the study. These were:

- Drs. A. van Linge (PI) and Drs. R.M. Metselaar
  Erasmus Medical Center (MC), Rotterdam
  Tel.: +31 10 703 3230
- Dr. P. Merkus (PI), Drs. N. de Boer and Drs. D.E.L. Mutsears
  Free University MC, Amsterdam
  Tel.: +31 20 444 3690
- Drs. R. van Haastert (PI) and Drs. M.E.C. Raming
  WestFriesGasthuis Hospital, Hoorn
  Tel.: +31 229 257 222
- Dr. G.G. Kingma
  MC Alkmaar, Alkmaar
  Tel.: +31 72 548 3138
- Dr. A. Kropveld
  St. Elisabeth Hospital, Tilburg
  Tel.: +31 13 539 2458
- Dr. A.F. Holm
  Wilhelmina Hospital, Assen
  Tel.: +31 592 325 225

A list of all participating investigators with brief curricula vitae (CV) is presented in Appendix 16.1.4.

- 1. **STUDY SPONSOR**

The study was sponsored by OctoPlus Technologies BV, Zernikedreef 12, 2333 CL Leiden, The Netherlands.

The sponsor’s contact person was:

- M. Nell, PhD, MBA (until August 2008)
  Clinical Leader
- E-J van Hoogdalem, RPh, PhD (from August 2008)
  Chief Medical Officer
  Tel.: +31-71-5241082
  1. **INVESTIGATIONAL PRODUCT**

The eardrops medication was prepared at the LUMC, Albinusdreef 2, 2333 ZA Leiden, The Netherlands. The LUMC study pharmacist was:

- Drs. G. Slappendel (September 2006 ‑ September 2007)
  Department of Clinical Pharmacology and Toxicology
- Drs. J. Pander (September 2007 – July 2008)
  Department of Clinical Pharmacology and Toxicology
  1. **CONTRACT RESEARCH ORGANIZATION**

The contract research organization (CRO) involved in the study was IATEC BV, Pietersbergweg 9, 1105 BM Amsterdam, The Netherlands. The CRO was responsible for (the coordination of):

- Study monitoring
- Resolution of data queries
- MedDRA (Medical dictionary of Drug Regulatory Activities) coding of adverse events (AEs)
- WHO coding of prior and concomitant medication
- Report writing

The contact person of IATEC BV was:

- Marcel Bootsma, MSc (until November 2008)
  Project Manager
- Esther Broer, MSc (from December 2008)
  CRA Manager
  Tel.: +31 20 3149357
  1. **STUDY MONITORING, DATA MANAGEMENT AND STATISTICS**

**Monitoring**

OctoPlus Technologies BV had assigned IATEC BV to monitor the study. The tasks of the monitor were to check whether the CRFs were completed correctly and whether the correct procedures were adhered to, including the reporting of serious adverse events (SAEs).

**Data Safety Monitoring Board**

An independent Data Safety Monitoring Board (DSMB) was installed to evaluate the results of the interim analysis. For this purpose, a DSMB charter was written as part of Amendment 5, dated 07 May 2008, presented in Appendix 16.1.1.

The DSMB consisted of three voting members and four non-voting / open session attendees. The voting members were individuals who were impartial and independent of the Investigator(s), Sponsor and CRO, and who had no financial, scientific, or other conflict of interest with the study.

The DSMB voting members for this study included experts or representatives in the field of:

1. Chair Name/Credentials: Prof. Dr. Peter van Brummelen/ Expert in Clinical Drug Development

Address: C. van Renneslaan 19, 1217 CW Hilversum, The Netherlands

Tel.: +31 35 6219268

1. Member Name/Credentials: Prof. Dr. Theo Stijnen/ Professor of Biostatistics

Address: Room S-05-34, Einthovenweg 20, 2333 ZC Leiden, The Netherlands

Tel.: +31 71 5269701

1. Member Name/Credentials: Dr. Rolien Free/ ENT Specialist

Address: ENT Dept., Groningen University Medical Centre, Hanzeplein 1, 9700 RB Groningen

Tel.: +31 50 3612730

The non-voting / open session attendees were**:**

1. The PI of the study: Drs. F.A.W. Peek
2. The Biostatistician: Dr. Ronald Brand
3. The sponsor representative: Dr. Ewoud Jan van Hoogdalem
4. The CRO representative: Drs. Marcel Bootsma

**Data management**

The data were entered in a data base by the department of Medical Statistics & BioInformatics of the LUMC.

The data management contact person was:

- Thekla Jansen-Werkhoven, PhD
  Tel.: +31-71-5269725

Coding of the AE and medication data was done by IATEC BV, see Section 6.4.

**Statistics**

The statistical analysis was done by the department of Medical Statistics & BioInformatics of the LUMC. The biostatistician who performed the statistical analysis was:

- Ronald Brand, PhD
  Tel.: +31-71-5269702

1. **INTRODUCTION**
   1. **CHRONIC SUPPURATIVE OTITIS MEDIA**

Upper respiratory tract infections, in particular otitis media and sinusitis, are highly prevalent among infants and young children, and to a lesser extent in adolescents and adults.^1^  Infections in the middle ear and the sinuses can lead to a vicious cycle of inflammatory reactions: incomplete recovery of the inflamed mucosa may lead to recurrent and persistent infections. During these infections, bacterial toxins like lipopolysaccharide (LPS) from gram-negative bacteria (GNB) and lipoteichoic acid (LTA) from gram-positive bacteria (GPB), play a role in continuing the vicious process. These products can induce an inflammatory reaction in the middle ear or in the sinuses that can induce injury to the mucosa of the upper airway epithelia.^2,3^ Due to this mucosal damage, the major defense system of the upper airways, the mucociliary clearance system, is disturbed and unable to restore the healthy equilibrium.
Children may suffer from long-term negative effects with respect to their development of speech and cognitive abilities because of the hearing loss that accompanies otitis media.^2^ Otitis media can also occur in adults, although less frequently than in children. In adults, a chronic alteration of the mucosa is more often present, together with perforation of the tympanic membrane. Approximately 2% of the population has significant health problems due to chronic suppurative otitis media (CSOM).^5^

For most patients with upper airway infections the current trend is to treat the infection with antibiotics. However, when treatment with antibiotics is temporarily successful, the symptoms often return within 30 days after the end of treatment.^6^ Adults are often treated with systemic antibiotics, and, in case of perforation of the tympanic membrane, with local antibiotics, but in a substantial part of the patients without persistent effect. For these reasons, and because of the growing resistance of bacteria to antibiotics, it is becoming increasingly important to develop alternative and more efficient strategies in the battle against chronic upper airway infections.

- 1. **OP-145**

OP-145 is a synthetic peptide, derived from the human cathelicidin LL-37. LL-37 is a cationic protein, produced by neutrophils and epithelial cells and is also secreted by, among others, the epithelium of the airways and the skin.^7,8^ Apart form having a direct antimicrobial role, LL-37 also acts as mediator in the inflammatory response and has an impact on processes like proliferation, immune induction, wound healing, cytokine release and chemotaxis.^9,10^ LL-37 is involved in the immune response against GNB and GPB.^11^ It strongly binds to both LPS of GNB as to LTA of GPB and neutralizes their pro-inflammatory activity. Hence, LL-37 seems to be of importance in controlling inflammatory reactions; during inflammatory processes, an increased expression of LL-37 has been shown.^12,13^

Recruitment of inflammatory cells to sites of microbial invasion, as induced by the chemotactic properties of LL-37, may be beneficial during acute infection. For the treatment of chronic infections, however, it is more favorable to dampen the immune response and not to attract more inflammatory mediators. Based on this, researchers from LUMC and OctoPlus have developed a novel synthetic peptide that is derived from the human antimicrobial peptide LL-37 with comparable LPS- and LTA neutralizing activity as LL-37 but with a very low chemotactic activity towards neutrophils as compared to LL-37.^14^

- 1. **RATIONALE**

In view of the above, a phase 2 program was carried out to investigate whether the direct application of OP-145 in the middle ear of adults with CSOM and tympanic perforation was safe and potentially effective with respect to inducing improvement in the mucosa of the middle ear. In the dose-finding study of the program, the results of which have been reported separately, the lowest potentially effective concentration was determined in 16 subjects, treated in cohorts of four, with doses of 0.25, 0.5, 1.0 and 2.0 mg/ml OP‑145. The product appeared to be safe and well tolerated at all four doses tested. Based on the results, the 0.5 mg/ml dose was selected for the double-blind placebo-controlled study of the program, the results of which are presented in the current report.

1. **STUDY OBJECTIVES**

**Primary objective**

The primary objective of the study was to investigate whether OP-145 could be safely applied directly on the inflamed mucosa of the middle ear of adults with CSOM (without cholesteatoma) and with perforation of the tympanic membrane.

**Secondary objective**

The secondary objective of the study was to obtain proof of efficacy for OP-145 in inducing improvement in the mucosa of the middle ear of adults with CSOM as compared to placebo.

# INVESTIGATIONAL PLAN

## OVERALL STUDY DESIGN AND PLAN – DESCRIPTION

This was a randomized, double blind, placebo-controlled, multicenter phase 2 study, to investigate the safety and efficacy of OP-145 in adults with CSOM, without cholesteatoma. (In the remainder of the text, the term CSOM is used for CSOM without cholesteatoma.) Subjects were randomized to be treated with eardrops containing OP-145 (active treatment) or consisting of vehicle only (placebo). The eardrops were to be applied twice a day for 2 weeks. Subjects attended the clinic at screening (Week 0) and in Weeks 1, 2, 4, 8 and 12.
The following tests and assessments were performed during the study: medical history (screening), physical examination, otoscopy, audiometry, CT scan of the middle ear and mastoid (within 6 months of study start), swabs from the middle ear and throat for bacterial culture, recording of prior and concomitant medication, AEs, laboratory tests (specific peptide antibodies and general hematology) and completion of the following quality of life (QoL) questionnaires: the SF-36, the chronic ear survey (CES) and the Brief Illness Perception Questionnaire (IPQ-b).

The treatment was regarded a success if an improvement of ≥ 2 points was observed at one or more otoscopic inspections in the following score:

0: flat, dry mucosa of the middle ear
1: flat, discharging mucosa of the middle ear
2: thickened, polypoid, dry or discharging mucosa of the middle ear
3: thickened, polypoid middle ear mucosa with viscous mucosal
 discharge

A maximum of 52 subjects was planned to be included in the study, 26 in each treatment group. A planned interim analysis was scheduled to be executed after 26 subjects had completed the Week 12 visit. The flexible design used an alpha and beta spending function and hence the specific time of analysis was allowed to depend on logistical feasibility. At the time this analysis was actually performed, the Week 12 data were available of 30 subjects (cut-off date 28 May 2008) and, because the formal stopping rule was met – with a large difference in efficacy observed between treatment groups - irrespective of the efficacy measure used or the statistical model employed, further accrual in the study was stopped. As a consequence, the interim analysis was to be regarded as the final efficacy analysis. The present report describes the safety and confirmatory efficacy results of all subjects treated in the study.

The study was originally designed as a phase 2 program of 2 parts. After completion of the dose-finding study in the program, the results of which have been reported separately, the protocol was amended (Amendment 2, dated November 18, 2005) to allow continuation as a randomized phase 2 study with multiple participating centers. This Amendment 2 to protocol P02.216 was the basis for the present report.

## DISCUSSION OF STUDY DESIGN, INCLUDING THE CHOICE OF CONTROL GROUPS

To date, there is no adequate treatment for CSOM in adults. Therefore, in the present study, the results of active treatment were compared to placebo. By using a placebo, any potential effects of the treatment with eardrop vehicle could also be investigated.

Because of the potential benefit and expected tolerability of direct application of OP‑145 to the middle ear mucosa of subjects with CSOM it was decided to perform a phase 1-2 study in subjects with CSOM , rather than executing this study as a safety study in healthy subjects. In addition, since CSOM subjects typically have a perforation of the tympanic membrane allowing direct access of the eardrop to the middle ear, the safety assessment was considered as more relevant in this population, rather than in healthy subjects who have an intact tympanic membrane.

## SELECTION OF STUDY POPULATION

Subjects were recruited from the coordinating center LUMC in Leiden and from nine other Dutch Hospitals. Subjects could be included in the study if they met the following eligibility criteria.

### Inclusion criteria

1. Adults ≥ 18 year, male and female;
2. Legally competent, no psychiatric history;
3. Diagnosis of CSOM with chronic proliferative mucosal changes > 6 months;
4. A clear perforation of the tympanic membrane to allow proper inspection of the middle ear mucosa;
5. Antibiotic therapy resistant, defined as having received adequate treatment for CSOM for at least 2 periods of in total ≥ 6 weeks within the past year with at least two different eardrops from the list presented in Section 9.4.7 and the last treatment period having occurred within the last 6 months before screening.

### Exclusion criteria

1. Cholesteatoma in the ear to be treated (i.e. CSOM with cholesteatoma);
2. Presence of a radical cavity in the ear to be treated;
3. Use of systemic immune suppressants or antibiotics, use of topical antibiotics, corticosteroids or other eardrops in one of the ears until 4 weeks before study start;
4. Down’s syndrome or other congenital anomalies to the external or middle ear or to the area of the “Eustachian tube – middle ear” of the ear to be treated;
5. Presence of immune disorders, e.g. primary immune deficiency, immune proliferative disorders, Multiple Sclerosis, Crohn’s Disease, rheumatoid arthritis or Primary Ciliary Dyskinesia;
6. Severe dizziness or severe headache, impacting on subjects’ daily life activities;
7. Facialis nerve disorders on the side of the ear to be treated;
8. Pregnancy, the wish to become pregnant or to breastfeed during the study, or, in case of a male subject, the wish to make his partner pregnant during the study;
9. Prior participation in the dose-finding study of the program.

### Removal of subjects from therapy or assessment

Subjects could be withdrawn prematurely from the study in case of:

1. SAE leading to hospitalization of the subject;
2. Sudden deafness or increasing sensorineural hearing loss;
3. Dizziness, facialis nerve disorders or severe headache;
4. Subject’s request or non-compliance.

The End of Study page of the CRF was to be completed for subjects who were withdrawn prematurely from the study and, if possible, the end-of-study (Visit 6, Week 12) data were to be collected. In case of (S)AE, the appropriate care was to be given.

All prematurely withdrawn subjects were to be replaced.

## TREATMENTS

### Treatments administered

Subjects were instructed to apply, at home, a few eardrops (± 100 μl) on the tympanic membrane, twice daily for 2 weeks (14 days).

If possible, they were to ask somebody else to apply the eardrops. The subjects were to lay down, with the affected ear pointing upwards. The person helping was to drop two eardrops in the affected ear, gently pulling the ear shell backwards and paying close attention to the flask not touching the ear. The subjects were to remain in the same position for 3 minutes, to allow the eardrops to set.

### Identity of investigational product

OP-145 is a synthetic peptide, derived from the human cathelicidin LL-37. LL-37 is a cationic protein with antibacterial properties, present in neutrophils and epithelial cells. OP-145 consists of 24 amino acids, contains an acetylated N-terminus and an amidated C-terminus, and can be represented by the following sequence: Ac‑IGKEFKRIVERIKRFLRELVRPLR-NH_2_.
OP-145 was produced according to GMP standards in the peptide laboratory of the Interdivisional GMP facility of the LUMC.

The formulation for reconstitution was prepared by the LUMC pharmacy in 10 ml HDPE (high-density polyethylene) eardrop bottles. The reconstitution fluid consisted of:

- 50 mM Sodium acetate buffer pH 5.7
- 1 mg/ml EDTA
- 0.2% benzalkonium chloride
- 7% PEG 10000
- NaCl to isotonicity

An overview of the eardrops used in the study is presented below.

**Active drug**:

Active compound: OP-145

Manufacturer: Interdivisional GMP facility of LUMC

Presentation: 10 ml HDPE eardrop bottles

Excipients: Sodium acetate, EDTA, benzalkonium chloride, PEG 10000, NaCl

Dosage form: Fluid for topical application, eardrops

Strength: 0.5 mg/ml

Manufacturer: LUMC pharmacy

Batch used: 03D040002

Storage conditions: In refrigerator at 2-8^0^C

**Placebo**:

Active compound: None

Manufacturer: Not applicable

Presentation: 10 ml HDPE eardrop bottles

Excipients: Sodium acetate, EDTA, benzalkonium chloride, PEG 10000, NaCl

Dosage form: Fluid for topical application, eardrops

Strength: 0.0 mg/ml

Manufacturer: LUMC pharmacy

Batch used: 07A2273

Storage conditions: In refrigerator at 2-8^0^C

### Method of assigning subjects to treatment groups

A randomization list was prepared by the statistician before study start and sent electronically to the LUMC pharmacy, responsible for the distribution of study drug. Randomization was done stratified by center, via a balanced block design. To prevent disbalance, centers were to complete entire blocks, even if this would result in the accrual of more subjects than anticipated. It was assumed that per center, 6 subjects would be enrolled. Therefore, a variable block design with block sizes of 2 and/or 4 subjects were possible. If the expected number of subjects to be accrued was smaller than 9 in a specific center, a fixed block size of 2 was to be used.

All subjects meeting the eligibility criteria were randomized after signing the informed consent form. Subjects with one-sided proliferative changes in the middle ear mucosa were randomized to receive either OP-145 or placebo. In subjects with proliferative changes in the middle ear mucosa of both ears, only one of the two ears was treated.

A detailed description of the randomisation method, including how it was executed, is provided in Appendix 16.1.7.

### Selection of doses in the study

*In vitro* tests have shown that OP‑145 is capable of neutralizing 1 ng/ml LPS for 50% at a concentration of 2-4 μg/ml and for 100% at a concentration of 5-8 μg/ml.^14^ Depending on the type of effusion, a mean concentration of 1.5, 18 or 96 ng/ml LPS is present in the middle ear.^15^ Assuming an LPS concentration of 100 ng/ml, then a OP-145 concentration of 1 mg/ml would neutralize the LPS present in the middle ear. In the dose-finding study of the development program, an OP-145 concentration of 0.5 mg/ml appeared to be optimal, as detailed in section 9.2 of the study protocol (Appendix 16.1.1).

### Selection and timing of dose for each subject

The subjects were instructed to apply two eardrops in the affected ear twice daily, in the morning and in the evening. They were to store the eardrops in the refrigerator until 30 minutes before use. After use, they were to place the eardrops back in the refrigerator immediately.

### Blinding

The randomization list was sent by the statistician to an independent person of the LUMC pharmacy, who numbered the blinded eardrop bottles to be dispensed to the participating centers and subjects according to this list. The list with the treatment codes was kept by this independent person at the LUMC pharmacy. At the end of the study, this list with treatment codes was used to identify the subjects who had received OP-145 and those who had received placebo.

### Prior and concomitant therapy

Subjects were to have received prior antibiotic therapy for CSOM for at least 2 periods of in total ≥ 6 weeks within the past year with at least two different eardrops from the list presented below (see also Inclusion Criteria in Section 9.3.1).

Table 9‑1: List of eardrops for the prior treatment of CSOM

| **Active substance(s)** | **Registered name** |
| --- | --- |
| Ofloxacine | Trafloxal |
| Dexamethason/chlooramfenicol/polymyxine B | Chloorampoldex |
| Dexamethason/framycetine/gramicidine | Sofradex |
| Dexamethason/tobramycine | Tobradex |
| Dexamethason/neomycine/polymyxine B FNA Flumetason/clioquinol | Locarten-Vioform |
| Fluocinolon/neomycine/polymyxine B | Synalar Bi-otic |
| Hydrocortison/oxytetracycline/polymyxine B | TerraCortril/polymyxine B |
| Hydrocortison/neomycine/polymyxine B | Otosporin |
| Hydrocortison/bacitracine/colistine | Bacicoline B |
| Fludrocortison/neomycine/polymyxine B | Panotile |

All medication used during the 6 months before study entry was to be recorded in the CRF.

During the study, the following medication was not allowed:

- Systemic immune suppressants
- Systemic antibiotics
- Topical antibiotics, corticosteroids or other eardrops

Other medication was only allowed in consultation with the investigator. All medication used during the study entry was to be recorded in the CRF.

### Treatment compliance

Subjects were instructed verbally and in writing on when and how to self administer the treatment.

The amount of eardrops dispensed and returned was recorded by the pharmacist or his/her designee. The bottles were weighed before they were dispensed and after they were returned to document drug use.

## EFFICACY AND SAFETY VARIABLES

### Efficacy and safety measurements assessed and flow chart

The parameters that were evaluated during the study are presented in Table 9‑2.

Table 9‑2: Study flow chart

| **Week:** | **W0** | **W1** | **W2** | **W4** | **W8** | **W12** |
| --- | --- | --- | --- | --- | --- | --- |
| **Assessment/Procedure Visit:** | **V1** | **V2** | **V3** | **V4** | **V5** | **V6** |
| Informed consent | X |  |  |  |  |  |
| Eligibility criteria check | X |  |  |  |  |  |
| Randomization | X |  |  |  |  |  |
| Demographics and medical history | X |  |  |  |  |  |
| Physical exam | X |  |  |  |  |  |
| CT scan^1^ | X |  |  |  |  |  |
| Swabs from middle ear and throat^2^ | X |  |  |  |  | X |
| Treatment^3^ |  | X | X |  |  |  |
| Otoscopy^4^ | X | X | X | X | X | X |
| Audiometry | X | X | X | X | X | X |
| High pitch audiometry | X |  |  |  |  | X |
| AEs | X | X | X | X | X | X |
| Prior and concomitant medication | X | X | X | X | X | X |
| Blood sample draw^5^ | X |  |  |  | X |  |
| QoL questionnaires | X | X | X | X | X | X |

CT scan of the middle ear and mastoid, made within 6 months of study start.

Swabs from middle ear and throat for bacterial culture; for Visit 1 this was within 2 weeks of study start.

Twice daily application of a few eardrops (± 100 μl) on the tympanic membrane, for 2 weeks (14 days) at home.

Inspection of the tympanic membrane and mucosa of the middle ear.

Within 2 weeks of study start and at the Week 8 visit (Visit 5), for the determination of specific peptide antibodies and for general hematology tests (Hb, Ht, ESR and differentials).

**Screening assessments (Week 0, Visit 1)**

The following information was collected and the following assessments/procedures were performed at screening:

- Age, gender, medical history, physical examination;
- Disease history;
- CT scan of the middle ear and mastoid (made within 6 months of study start) (see Section 9.5.5.3.2);
- Swabs from the middle ear and throat for bacterial culture (within 2 weeks of study start) (see Section 9.5.5.2.3);
- Otoscopic inspection of the tympanic membrane and middle ear mucosa (see Section 9.5.3.1);
- Audiometry and high-pitch audiometry (see Section 9.5.5.3.3);
- Recording of AEs (see Section 9.5.5.1);
- Recording of prior and concomitant medication (see Section 9.4.7);
- Blood samples for the determination of specific peptide antibodies and for general hematology tests (Hb, Ht, ESR and differentials) (within 2 weeks of study start) (see Section 9.5.5.2);
- Completion of QoL questionnaires (see Section 9.5.3.2).

**On-study assessments (Week 1-8, Visits 2, 3 4 and 5)**

The following assessments/procedures were performed on-study:

- Otoscopic inspection of the tympanic membrane and middle ear mucosa:
  at each visit (see Section 9.5.3.1);
- Audiometry: at each visit (see Section 9.5.5.3.3);
- Recording of AEs (see Section 9.5.5.1);
- Recording of concomitant medication: at each visit (see Section 9.4.7);
- Blood samples for the determination of specific peptide antibodies and for general hematology tests (Hb, Ht, ESR and differentials): only at Visit 5 (Week 8) (see Section 9.5.5.2);
- Completion of QoL questionnaires: at each visit (see Section 9.5.3.2).

**Final assessments (Week 12, Visit 6)**

The following assessments/procedures were performed at Visit 6 (Week 12):

- Swabs from the middle ear and throat for bacterial culture (see Section 9.5.5.2.3);
- Otoscopic inspection of the tympanic membrane and middle ear mucosa:
  at each visit (see Section 9.5.3.1);
- Audiometry and high-pitch audiometry (see Section 9.5.5.3.3);
- Recording of AEs (see Section 9.5.5.1);
- Recording of concomitant medication: at each visit (see Section 9.4.7);
- Completion of QoL questionnaires (see Section 9.5.3.2).

### Appropriateness of measurements

Methods used in this study for assessment of safety and efficacy are viewed as standard clinical procedures, i.e. widely-used for this indication and generally recognized as reliable, accurate and relevant.

### Efficacy variables

#### Primary efficacy variable

The primary efficacy variable was recovery of the middle ear mucosa and tympanic membrane. The treatment was regarded a success if an improvement of ≥ 2 points was observed at one or more otoscopic inspections in the following score:

0: flat, dry mucosa of the middle ear
1: flat, discharging mucosa of the middle ear
2: thickened, polypoid, dry or discharging mucosa of the middle ear
3: thickened, polypoid middle ear mucosa with viscous mucosal
 discharge

Otoscopy was performed at screening and at Weeks 1, 2, 4, 8 and 12.

#### Secondary efficacy variable

The secondary efficacy variable was improvement in QoL, as assessed by completion of the following three QoL questionnaires:

- The SF-36.
  The SF-36 is a multi-purpose, short-form health survey with 36 questions. It yields an 8-scale profile of vitality, physical functioning, bodily pain, general health perceptions, physical role functioning, emotional role functioning, social role functioning and mental health.
  It is a generic measure, as opposed to one that targets a specific age, disease, or treatment group. Accordingly, the SF-36 has proven useful in surveys of general and specific populations, comparing the relative burden of diseases, and in differentiating the health benefits produced by a wide range of different treatments.^16,17^
- The CES.
  This is the only validated disease-specific questionnaire for CSOM.^18^ One additional question was added to this questionnaire: “Do you think the treatment was effective” (“Heeft u het idee dat de behandeling gewerkt heeft”).
- The IPQ-b (in Dutch referred to as the “IPQ-K”).
  This is an abbreviated version of a questionnaire representing the emotional and cognitive status.^19^

The QoL questionnaires were completed at screening and at Weeks 1, 2, 4, 8 and 12. Dutch versions of these questionnaires were used.

### Drug concentration measurements

No drug concentration measurements were performed.

### Safety variables

Safety was the primary endpoint of the study. To assess safety, the following assessments were performed: AEs were recorded, blood samples were taken for the determination of specific peptide antibodies and general hematology laboratory tests, swabs were taken from the middle ear and throat for bacterial culture, audiometry was performed.
All resulting data were to be recorded in the subject’s medical file and in the CRF, with the exception of the anti-OP-145 antibody analyses that had no therapeutic consequence and were recorded in the study data base only.

#### Adverse events

All AEs and SAEs that occurred during the study were to be recorded in the subjects’ medical file and in the CRF. Subjects were to receive the proper care and, in case of an SAE, subject was to discontinue study treatment.

SAEs were to be reported immediately as per ICH guidelines to the Sponsor, the study coordinator and the IEC, with comments of the investigator.

An SAE was defined as any untoward medical occurrence that at any dose resulted in death, was life-threatening, required hospitalization or prolongation of existing hospitalization, resulted in persistent or significant disability/incapacity or was a congenital anomaly/birth defect.

#### Laboratory tests

All samples and swaps taken from the subjects were coded. The investigator kept a list of the codes, identifying which samples and swaps were taken from each subject. At the end of the study, the codes were broken and the results of the examinations were linked to the subject in question. Any unused material remaining at the end of the study was to be destroyed.

##### Determination of specific peptide antibodies

At screening and at Week 8, blood samples were drawn for the determination of specific peptide antibodies. The serum prepared from this sample was to be stored at ‑20°C and was to be transferred by the monitor to Covance for central analysis.

Anti-OP-145 antibody detection was done at Covance Laboratories Ltd., Otley Road, Harrogate, North Yorkshire, HG3 1PY, United Kingdom. The Covance study director was Ross Bamford, Department of Protein and Immunochemistry, Division of Biotechnology, Tel.: +44-1423-848836.

##### General hematology tests

At screening and at Week 8, blood samples were drawn for the determination of hemoglobin (Hb), hematocrit (Ht), erythrocyte sedimentation rate (ESR) and white blood cell differentials. These tests were performed locally.

##### Bacterial cultures

At screening and end at Week 12, swabs were taken from the middle ear and throat to investigate the type of bacteria present at study start and to evaluate whether a change had occurred in the type of microorganisms present.
The bacterial cultures were performed locally according to standard procedures.

#### Physical examination

##### General physical examination

At screening, a general physical examination was performed.

##### CT scan

At screening, a CT scan was made of the middle ear and mastoid to exclude the presence of a radical cavity, suspicion of cholesteatoma and other disorders requiring exclusion of the subject.

##### Audiometry and high-pitch audiometry

At screening and at Weeks 1, 2, 4, 8 and 12, audiometry was performed. In addition, high-pitch audiometry was done at screening and at Week 12. The tests were performed in the out-patient clinic by an audiometry assistant.

These tests were used to determine the eventual occurrence of sensorineural hearing loss.

## DATA QUALITY ASSURANCE

The monitor, assigned to the study by the Sponsor, checked during and at the end of the study whether all procedures were adhered to correctly. The monitor also checked whether the CRFs were completed correctly.

All data were entered in a data base of the department of Medical Statistics & BioInformatics of the LUMC. AE and concomitant medication data were transferred electronically to IATEC for coding into MedDRA.

## STATISTICAL METHODS PLANNED IN THE PROTOCOL AND DETERMINATION OF SAMPLE SIZE

### Statistical and analytical plans

The reports for the interim and confirmatory efficacy analyses are presented in Appendix 16.1.9. The data were analyzed using a Statistical Package for the Social Sciences (SPSS).

All data were listed by subject and summarized by means of descriptive statistics including means, standard deviations, medians and ranges, or frequencies and percentages as appropriate.

#### Analysis populations

Two populations were distinguished:

1. The intention-to-treat (ITT) population, consisting of all randomised subjects who had received at least one dose of study medication;
2. The per-protocol (PP) population consisting of all randomised subjects who had received at least one dose of study medication and had no major protocol violations.

The confirmatory efficacy analysis was performed on the ITT and PP populations; the safety analysis was performed on the ITT population.

#### Efficacy analysis

The treatment was regarded a success if an improvement of ≥ 2 points was observed at one or more otoscopic inspections in the following score:

0: flat, dry mucosa of the middle ear
1: flat, discharging mucosa of the middle ear
2: thickened, polypoid, dry or discharging mucosa of the middle ear
3: thickened, polypoid middle ear mucosa with viscous mucosal
 discharge

The percentage of subjects with treatment success at end of the study was compared between groups by means of a Fisher Exact test.
In addition, a repeated measures analysis of variance was performed with the outcome score as the dependent variable and time (baseline to Week 12) as the independent covariate, assuming various covariance structures. As a very close approximation which has the benefit of not depending on the choice of covariance structure, also a regression line was fitted per subject after which the slope and intercept of those lines were compared between the randomization groups. Since the results of this (simplified) approach were almost identical to the results of the repeated measurements analyses of variance, this approach was then used in the interim and final report.

SF-36 QoL data were analyzed by comparing both the average individual scores and the sum scores between groups. Statistical comparison was done in exactly the same way as for the primary efficacy variable: by fitting a regression line per subject over time and comparing the (average) slopes.
For the IPQ-b questionnaire and the one question added to the CES, the analysis was limited to a comparison between groups of the Week 12 results. The other CES data were not analyzed.

#### Safety analysis

Before the AE data were listed and summarized, they were coded using MedDRA version 9.

#### Interim analysis

An interim analysis using a flexible design and alpha and beta spending function, was pre-specified in the protocol after 26 subjects would have completed the Week 12 visit. The study was to be terminated in case of:

- Safety:
  In ≥ 4 subjects one or more of the following events had occurred: an SAE leading to hospitalization of the subject, sudden deafness or increasing sensorineural hearing loss, dizziness, facialis nerve disorders or severe headache.
- Efficacy:
  The null-hypothesis, there is no difference between treatment groups, could be rejected (efficacy), or the alternative hypothesis, there is a difference between treatment groups, could be rejected (futility).

The alpha and beta spending functions were fully specified in the protocol. If the study was to be terminated because of the results of the interim efficacy analysis, the data of the subjects enrolled in the study during the execution of the interim analysis, were to be collected until the last (Week 12) visit. The efficacy data of these subjects, however, were to be used for confirmatory purposes only, because the results of the interim efficacy analysis were to be regarded as the final efficacy analysis. The safety data of these subjects were included in the final safety analysis.

### Determination of sample size

Sample size calculations were performed with the software package East (version 3.1.0), which offers the possibility of interim analysis with an alpha and beta spending function.

It was assumed that the otoscopically assessed success rate in the OP-145 group would be 60% and in the placebo group 20% and the difference was to be detected with a power of 90% at a significance level alpha of 5% in a two-sided test. Using the approach of the O’Brien-Fleming boundaries in the context of alpha and beta spending, the study would have the power to show both efficacy and futility, and it was calculated that the maximum number of subjects to be included was 52, i.e. two groups of 26 subjects.

For an interim analysis performed on the data of 26 subjects, it was calculated that there was a chance of 16% that the study was terminated because of ‘no difference’ between groups (H0), while the efficacy was indeed the same in both groups. Likewise, it was calculated that there was a chance of 27% to terminate the study because of a significant difference between groups (H1), while the difference was indeed 60% versus 20%.

## CHANGES IN THE CONDUCT OF THE STUDY OR PLANNED ANALYSES

### Changes in the conduct of the study

The present study was originally designed as the double-blind placebo-controlled study part of a phase 2 clinical program in one single centre. After completion of the preceding dose-finding study, the results of which have been reported separately, the protocol was amended (Amendment 2, dated November 18, 2005) to allow continuation as a randomized phase 2 study with multiple participating centers. This Amendment 2 to protocol P02.216 was the basis for the present report.

After activation of the phase 2 part of the study, the protocol was amended three times.

Amendment 3, dated 10 May 2007, was prepared to implement the following changes:

- Only a pre-study CT scan needed to be made, instead of one pre-study and one at the end of follow up;
- The inclusion criterion of presence of pre-existing CSOM for 3 months was extended to 6 months;
- The exclusion criterion of pre-existing radical cavity was added;
- The exclusion criterion of ‘pre-existing immune disorder’ was extended to ‘pre-existing known immune disorders’;
- The exclusion criterion ‘presence of Down’s Syndrome’ was extended to ‘Down’s syndrome or other congenital anomalies to the external or middle ear’;
- The following questionnaires were added to the assessments; SF-36, CES and IPQ-b.

Amendment 4, dated 07 January 2008, was prepared to implement the following changes:

- Change in personnel: the pharmacist of the LUMC was replaced;
- Extension of the number of participating centers;
- Decrease of the average number of subjects to be included per center to six, because of the participation of more centers;
- Adjustment of the time schedule;
- Further specification of the in- and exclusion criteria.

The rationale for this amendment was the fact that further specification of the eligibility criteria was warranted in view of the multiple centers participating in the study, and that certain protocol details required adjustment.

Amendment 5, dated 07 May 2008, was prepared to implement the following changes related to the interim analysis to be performed:

- Originally, an interim analysis was planned after the data of 26 subjects were available (see Section 9.7.1.4). By means of Amendment 5, the interim analysis was planned as soon as the data of the first 24 subjects were available, allowing a balanced analysis within each center. The boundaries for efficacy and futility were to be adjusted to guarantee the overall power of the study. In doing so, it was calculated that with an interim analysis on 24 subjects there was a chance of 8% that the study was terminated because of ‘no difference’ between groups (H0), while the efficacy was indeed the same in both groups. Likewise, it was calculated that there was a chance of 18% to terminate the study because of a significant difference between groups (H1), while the difference was indeed 60% versus 20%.
  In reality, the interim analysis was performed on the data of 30 subjects.
- The results of the interim analysis were evaluated by an independent DSMB, to assure participant safety and study integrity, and to estimate the overall benefit/risk balance of study continuation. The primary responsibilities of the DSMB were:
- Initially, to familiarize themselves with the research protocol, informed consent documents, and the DSMB Charter (presented in Appendix 16.1.1).
- To evaluate the progress of the trial, which included:
  - AE incidence, participant dropouts, compliance or any complaints
  - Data quality for completeness, timeliness and accuracy
  - Participant recruitment, accrual and retention
  - Looking at risks vs. benefits
  - Reviewing any other factors that might have affected study outcome.
- To consider factors external to the study when relevant information became available, such as scientific or therapeutic developments that might have had an impact on the safety of the participants or the ethics of the trial.
- To ensure confidentiality of trial data and results of monitoring.
- To ensure that blinding of the study was maintained outside of the DSMB, including to PI, Sponsor, CRO and study sites.
- To make recommendations to the PI and Sponsor regarding study continuation, termination or modifications based on review of the safety and efficacy data.

The changes implemented by means of these amendments are incorporated in the present report without further specification. Copies of the amendments and the DSMB charter are provided in Appendix 16.1.1.

### Changes in the planned analyses

The primary efficacy outcome was originally planned to be analysis by means of repeated measurement analysis of variance with the zero measurement as covariate, in addition to non-parametric analysis of the rate of treatment success in the two treatment groups (Fisher Exact test). Several mixed models were fitted to compare the two treatment groups, assuming different covariance structures. Since the results from these different models were extremely close, the simpler regression approach described in Section 9.7.1.2 was used, in which the outcome score was taken as the dependent variable and time as the independent covariate, where there was no need to specify a covariance structure.

The analysis of the QoL data was not planned for in detail, nor was the study specifically powered to detect clinically relevant changes in QoL scores with statistical significance. The SF-36 QoL data were analyzed in exactly the same way as the primary efficacy variable: by taking the (domain) score as the dependent variable and time as the independent covariate and by subsequently fitting a regression line per subject over time and comparing the (average) slopes.
For the IPQ-b questionnaire and the one question added to the CES, the analysis was limited to a comparison between groups of the Week 12 results. The other CES data were not analyzed.

# STUDY SUBJECTS

## DISPOSITION OF SUBJECTS

In total 41 subjects were randomized in the study between 19 September 2006 and 15 April 2008. Seven subjects were never treated for the following reasons:

1. Three subjects (102-03, 106-04, and 110-02) did not provide informed consent.
2. Three subjects (103-02, 108-01 and 109-01) did not meet the eligibility criteria.
3. One subject (104-02) had a radius fracture after randomization and before treatment. The event was considered an SAE because the subject was hospitalized for treatment of the fracture. Subsequently, subject did no longer meet the eligibility criteria and was not treated in the study.

In total 34 subjects had received study treatment, 17 in each treatment group. An individual listing of study dates is presented by subject in Appendix 16.2.1.1. An overview of the accrual is presented by center and overall in Table 10‑1.

Table 10‑1: Overview of number of subjects treated by center; ITT population (N=34)

|  | **OP-145** | | **Placebo** | | **Overall** | |
| --- | --- | --- | --- | --- | --- | --- |
| **Code: Study centre** | **n** | **(%)** | **n** | **(%)** | **n** | **(%)** |
| 101: LUMC, Leiden | 9 | (53) | 9 | (53) | 18 | (53) |
| 102: Erasmus MC, Rotterdam | 1 | (6) | 1 | (6) | 2 | (6) |
| 103: Free University MC, Amsterdam | 2 | (12) |  | (0) | 2 | (6) |
| 104: WestFriesGasthuis Hospital, Hoorn | 0 | (0) | 1 | (6) | 1 | (3) |
| 105: MC Alkmaar, Alkmaar | 3 | (18) | 2 | (12) | 5 | (15) |
| 106: St. Elisabeth Hospital, Tilburg | 1 | (6) | 2 | (12) | 3 | (9) |
| 110: Wilhelmina Hospital, Assen | 1 | (6) | 2 | (12) | 3 | (9) |
| **Total** | 17 | (100) | 17 | (100) | 34 | (100) |

Data are derived from the final statistical analysis report (finalanalysis.pdf) presented in Appendix 16.1.9.

See also Appendix 16.2.1.1.

In Appendix 16.2.1.2, the dates and reasons for end-of study are listed. For the 3 subjects who did not provide informed consent, this field was left empty. The data show that all treated subjects came off treatment because they had completed the study. The last subject had completed the study on 04 July 2008.

## PROTOCOL DEVIATIONS

In 8 subjects major protocol deviations had occurred leading to exclusion of these subjects from the PP analysis. An individual listing of these deviations is presented in Appendix 16.2.1.4. Individual data on adherence to the in- and exclusion criteria is presented by subject in Appendix 16.2.1.5. An overview of the major deviations from protocol is presented in Table 10‑2.

Table 10‑2: Overview of major protocol deviations; ITT population (N=34)

|  | **Subjects** | |
| --- | --- | --- |
| **Protocol deviation** | **OP-145** | **Placebo** |
| Subject received concomitant medication within 4 weeks of study start that was not allowed per protocol | 101-14 | 106-01 |
| Subject had a tympanostomy tube (grommet) | 101-06 |  |
| Subject received concomitant medication during the study that was not allowed per protocol | 101-03, 101‑16 102‑01 | 101-02, 104‑01 |

Data are derived from Appendices 16.2.1.4 and 16.2.1.5.

Note 1: the concomitant medication subject 101-11 received within 4 weeks of study start was an ointment applied on the skin and could not reach the middle ear; this subject was therefore included in all analyses even though this was recorded by the investigator as violation of the exclusion criteria (see Appendices 16.2.1.5 and 16.2.2.3).

Note 2: subject 106-01 did receive concomitant medication within 4 weeks of study start that was not allowed per protocol, but this was not recorded by the investigator as violation of the exclusion criteria (see Appendix 16.2.1.5).

Three subjects were excluded form the PP analysis because they had a major deviation from the eligibility criteria. Five subjects were excluded because they received concurrent antibiotic treatment. In total 5 subjects in the OP-145 group and 3 subjects in the placebo group had major protocol deviations.
